# Digging into boring bryozoans: new characters and new species of Immergentiidae

**DOI:** 10.1007/s13127-024-00645-y

**Published:** 2024-06-27

**Authors:** Mildred J. Johnson, Ahmed J. Saadi, Piotr Kuklinski, Abigail M. Smith, Juan López-Gappa, Thomas Schwaha

**Affiliations:** 1 Department of Evolutionary Biology, University of Vienna, Djerassiplatz 1, 1030 Vienna, Austria; 2 Institute of Oceanology, Polish Academy of Sciences, 81-712 Sopot, Poland; 3 Department of Marine Science, University of Otago, P.O. Box 56, Dunedin, 9054 New Zealand; 4 Consejo Nacional de Investigaciones Científicas y Técnicas (CONICET), Buenos Aires, Argentina; 5 Museo Argentino de Ciencias Naturales, C1405DJR Ciudad Autónoma de Buenos Aires, Buenos Aires, Argentina

**Keywords:** Ctenostome, *Immergentia stephanieae* sp. nov., *Immergentia pohowskii* sp. nov., Cardiac constrictor, Cystid appendage, Mitogenome

## Abstract

*Immergentia* is an endolithic genus of ctenostome bryozoans and the sole member of the Immergentiidae. Etchings of their typical spindled-shaped and sometimes enantiomorphic borehole aperture in calcium carbonate substrates are accomplished by chemical dissolution. The tentacle crown of the bryozoan is essentially the only body part that extends beyond the shell surface when protruded. Previously, species were mainly described using external colony and zooidal characteristics or whole mounts, with partial histological sections conducted on a single species in 1947. Modern approaches, however, are hitherto missing. We examined the soft body morphology of *Immergentia* from different locations with confocal laser scanning microscopy and the production of 3D reconstructions. In addition, zooidal characteristics such as tentacle number, size, tubulets, and interzooidal distances were used to distinguish and describe species. The combination of conventional and modern methods revealed the presence of a cardiac constrictor and intercalary kenozooids that can interpose between the cystid appendages, something not previously reported in immergentiids, thus necessitating an amendment of the family diagnosis. The polypide typically has eight to ten tentacles, and the anus is positioned in the low or mid-lophophoral area. In addition, sequence data, including the mitogenome and the nuclear ribosomal genes (18S and 28S) of four species from five locations, are presented for the first time. Based on molecular and morphological data, a novel intertidal immergentiid from France, *Immergentia stephanieae* sp. nov., and a subtidal species from New Zealand, *I. pohowskii* sp. nov., are described. This work supplements the rather sparse existing knowledge on Immergentiidae and proposes additional characteristics to complement existing descriptions in order to enhance future species identification.

**Supplementary Information:**

The online version contains supplementary material available at 10.1007/s13127-024-00645-y.

## Introduction

Bryozoans are mostly colonial, suspension-feeding invertebrates divided into two clades: Phylactolaemata, with strictly freshwater species, and its sister taxon Myolaemata (Schwaha et al., 2020; Taylor & Waeschenbach, 2015). The latter comprises the strictly marine Stenolaemata and Gymnolaemata, which are predominantly marine (Taylor & Waeschenbach, 2015). With the exception of few solitary forms, gymnolaemate bryozoans are colonial, formed of genetically-identical asexually-formed zooids. A zooid capable of feeding itself (an autozooid) is generally comprised of a cystid (protective body wall) and a retractable polypide (lophophore, a U-shaped digestive tract and associated neural and muscular tissue) (Schwaha et al., 2020). In myolaemates, the polypide of an autozooid can undergo cycles of degeneration and regeneration within its cystid.

Bryozoans are an integral part of the benthic environment, playing a role in reef formation (Cuffey, 1977, 2006; Dutka et al., 2022), biogenic skeletal carbonate build-up (see Senowbari-Daryan et al., 1993; Sharples et al., 2014), sediment formation, binding and stabilization (Cuffey, 1977; Taylor & Ernst, 2004), and providing habitat for benthic communities (Cocito, 2004; Taylor & Ernst, 2004; Wood et al., 2012).

Gymnolaemate bryozoans are comprised of two clades, the paraphyletic Ctenostomata (non-calcified), and monophyletic Cheilostomata (calcified), that originated from a ctenostome-like ancestor (Taylor & Larwood, 1990). Endolithic bryozoans, also commonly known as boring bryozoans, are ctenostomes. These organisms live embedded within calcium carbonate substrates, which they colonise by chemical dissolution. When protruded, the tentacle crown essentially extends beyond the surface of the housing shell, while the rest of the bryozoans’ body remains embedded within the substrate. Boring ctenostomes are known since the early Ordovician (Mayoral, 1991; Taylor & Rozhnov, 1996), but reports are still rare (see Pohowsky, 1975, 1978,) owing mainly to the non-calcified skeleton, which is often not preserved. Nonetheless, boring species occur in most oceans, the polar regions being the exception, which indicates a near-global distribution. However, the extent of global diversity is still poorly known.

To date, there are four families comprised of boring bryozoans: Immergentiidae Silén, 1946, Penetrantiidae Silén, 1946, Spathiporidae Pohowsky, 1978 and Terebriporidae d'Orbigny, 1847, each consisting of a single genus. All families of boring bryozoans have either true kenozooid stolons or pseudostolons interconnecting autozooids. The latter include cystid appendages, which are extensions of the cystid wall either at the apertural or mid-zooidal area of a zooid, so the terms primary and secondary cystid appendages are used to refer to these respective structures. Zooids of species assigned to Penetrantiidae and Spathiporidae are pedunculate, i.e. zooids are attached to the primary stolon by a stalk-like process. *Penetrantia* Silén, 1946 is broadly characterised by kidney-shaped apertures, the peduncle attaches to the distal end and zooids are vertically submerged (Decker et al., 2023; Silén, 1946) in the substrate. *Spathipora* Fischer, 1866 typically forms comma-shaped apertures, the peduncle attaches along mid-length, and zooids lay at an angle to the substrate (Pohowsky, 1978). *Terebripora* d'Orbigny, 1847 form more circular apertures, are non-pedunculate with stolons along mid-length of the zooid, and zooids lay parallel to the primary stolon (d’Orbigny, 1847; Pohowsky, 1978).

At present, 17 species are included in the genus *Immergentia*, 11 of which are recent (see https://www.bryozoa.net/ctenostomata/immergentiidae/immergentia.html). Family Immergentiidae is currently assigned to the Superfamily Arachnidioidea Hincks, 1880 owing primarily to the presence of mostly anastomosing cystid appendages which characterize families that belong to this group: the Aethozoidae d’Hondt, 1983, Arachnidiidae Hincks, 1880 and Nolellidae Harmer, 1915 (Schwaha & De Blauwe, 2020; Schwaha et al., 2019; Schwaha, 2020a).

Immergentiidae was erected by Silén (1946) with the description of two species, the type species of the genus *Immergentia* Silén, 1946, *I. californica* Silén, 1946 from Pacific Grove, California (USA), and *I. zelandica* Silén, 1946 from Slipper Island (New Zealand). Later, Silén (1947) provided anatomical details and also described a third species *I. suecica* Silén, 1947 from Gullmar Fjord (Sweden). Generally, immergentiids form spindle-shaped apertures, zooids extend from the primary cystid appendage and are vertical in the substrate with a slightly tilted basal tip. They are distinct from other endolithic ctenostomes in the following: (1) autozooids lack a gizzard in the digestive tract; (2) zooids are non-pedunculate and arranged vertically or oblique in the substrate; (3) they lack ‘true’ stolons; instead, they have cystid appendages that extend from the autozooid and interconnect with cystid appendages from other zooids in the colony (Silén, 1947). In other stolonate forms, the stolons are kenozooidal polymorphs that interconnect autozooids in a colony (Schwaha, 2020a). This characteristic also serves as evidence that an endolithic lifestyle evolved at least twice in recent boring species (Jebram, 1973; Schwaha, 2020a).

Following Silén’s work, Soule (1950) described *I. philippinensis* Soule, 1950 and *I. zelandica minuta* Soule, 1950 from the Philippine Islands and later *I. angulata* Soule & Soule, 1969 from the Hawaiian Islands (Soule & Soule, 1969). Pohowsky (1978) revised the taxonomy of fossil and recent boring bryozoans (in addition to other bryozoans/forms), including the description and characterization of novel and known genera and species. In the same work, Pohowsky (1978) described *I. patagoniana* Pohowsky, 1978 from Patagonia (Argentina) and *I. subangulata* Pohowsky, 1978 from the Bay of Santos (Brazil). Moreover, his analysis further revealed that a syntype of *Terebripora orbignyana* Fischer, 1866 from Arcachon (France) is actually an immergentiid subsequently named *I. orbignyana*. Afterwards, López-Gappa (1981) described *I. zelandica patagonica* López-Gappa, 1981 from Santa Cruz (Argentina). Then, d’Hondt (1983) created a tabular identification key for *Immergentia* species known at that time. Almost four decades later, Seo et al. (2018) described *I. cheongpodensis* Seo et al. (2018) from the Korean West Coast.

Since the publications of Silén and Pohowsky there have been few accounts of *Immergentia* in the seas of Western Europe. Both Prenant and Bobin (1956) and Hayward (1985) included the description of *I. suecica* (including the works of Silén (1946, 1947), Soule (1950), and Pohowsky (1978)) in their Faunas from France and the British waters respectively, but elucidating that to date no species of the genus had been found in both areas. However, in retrospect *I. orbignyana* (previously *T. orbignyana*) from France had been described in 1866. Pohowsky (1978) revealed fossil immergentiids from across Europe and almost a dozen unidentified specimen from the Miocene and one recent from France. Reverter et al. (1995) reported another recent representative of the genus *Immergentia*, which was found in the subtidal zone near Château du Taureau in Roscoff. This, in part, prompted our curiosity to search for and further investigate boring bryozoans in Roscoff as one of the primary research sites. In recent years, De Blauwe (2009) reported *I. suecica* on the Flemish Banks, and Reverter-Gil et al. (2016) reported abundant borings and colonies of *Immergentia* from the North-West Iberian coast. *Immergentia orbignyana* was included in species lists of bryozoans from the Mediterranean Sea (see Rosso, 2003 (previously *T. orbignyana*) and Rosso & Di Martino, 2016) based material by Fischer (1866), but its location and presence require verification (E. Di Martino, personal communication 08/12/2023).

The majority of *Immergentia* descriptions were based on either one or a combination of the following methods: traces of aperture shapes on shells, characterization of casts, colony structures/development, and whole mounts (see Silén, 1946, 1947; Soule & Soule, 1975; Pohowsky, 1978). These descriptions, photomicrographs, and drawings were quite valuable in detailing the characteristics of different species, with some species presented better than others. Unfortunately, Silén’s histological analysis of *I. californica* represents the only detailed drawing of the soft-body morphology of an immergentiid (Silén, 1947). The lack of any modern and more holistic analyses and little knowledge on the diversity of this family, called for a new approach to tackle this difficult group of bryozoans. Consequently, we sampled *Immergentia* species from different localities to (1) provide new soft-body morphological data revealed with immunocytochemical staining, histology and 3D-reconstructions, (2) provide the first sequence data of immergentiids, and (3) review all available data of the family to amend its diagnosis based on our broad morphological comparative data.

## Methodology

### Sampling

Substrates bearing boring bryozoans were collected from twelve locations in four different regions (Table S1). French samples (study site) were collected from the intertidal and subtidal zones (by dredging) with the research vessel *Neomysis* in and around Roscoff. Burdwood Bank, New Zealand, and Norway were locations of opportunity. Briefly, samples from Argentina were collected by cruises of the R/V Puerto Deseado from Burdwood Bank in the Magellan region, southwest Atlantic Ocean (see López-Gappa & Zelaya, 2021). Samples from New Zealand were collected during the PB Otago Shelf Cruise (Smith et al., 2023). Samples from Norway were collected from Trondheim Fjord.

All samples from locations of opportunity were examined, and those bearing immergentiid borings were selected for further analysis. Informed by preliminary sampling in Roscoff, gastropods from the intertidal zone were screened for living colonies of *Immergentia*. From the subtidal samples, shells or shell fragments from dead molluscs ranging from not eroded to slightly eroded were collected, especially if tissue was visible in the borehole apertures. Heavily eroded, bored, or colonized shells were not considered for analysis but for substrate identification if in a satisfactory state.

Type material from Lars Silén were obtained from the Swedish Museum of Natural History (SMNH) and examined. Type material from France has been deposited at the Muséum national d'Histoire naturelle, Paris, France (MNHN). Type material from New Zealand is deposited at the National Institute of Water & Atmospheric Research Ltd (NIWA). All other material collected for the purpose of this study is in the possession of the first author and currently stored at the University of Vienna Biology Building (UBB), Department of Evolutionary Biology.

### General sample preparation and decalcification

Samples were fixed in different solutions depending on the final analysis. Specimens for immunochemical staining were fixed in 4% paraformaldehyde for 2 h at room temperature and then refrigerated overnight. Specimens for histology were fixed in 2.5% glutaraldehyde for 24 h. After the fixation all samples were washed three times with a 0.1 M phosphate buffer PB for 20 min and stored in the same solution. For long term preservation samples were stored in 0.1 M PB with 0.1% sodium azide (NaN_3_) at 4 °C. Samples intended for DNA extraction were fixed in 96% or absolute ethanol.

Images of *Immergentia* colonies in substrate were taken with a Nikon Z6 camera attached to a Nikon SMZ800 stereomicroscope or with a Nikon SMZ25 stereomicroscope equipped with a DsRi2 camera (Nikon, Tokyo, Japan). To ease extraction of zooids, shells containing boring bryozoans were decalcified in 20% EDTA (pH 8.3). Duration of decalcifying depends on the shell lasting between 1 and 2 weeks. For longer decalcification, the solution was changed every 3–4 days. After decalcification samples were washed three times in PB (pH 7.3) at 15-min intervals between washes. Zooids from a colony were removed from the extracellular matrix of the shell and stored, as mentioned earlier. Where possible whole mounts of zooids were made on microscope slides with glycerol as the mounting medium. Coverslips were sealed with a varnish.

### Histology and 3D reconstruction

Methodology for creating serial sections and 3D reconstruction was based on Ruthensteiner (2008). Specimens were embedded into Agar Low Viscosity Resin (LVR, Agar Scientific Ltd., Stansted, UK). Semithin sections of 1 µm thickness were created with a Histo-Jumbo diamond knife (Diatome AG, Biel, Switzerland) mounted on a Leica UC6 ultramicrotome (Leica Microsystems GmbH, Wetzlar, Germany). Ribbons of semithin sections were transferred onto microscope slides and stained with 0.1% toluidine blue for 10 s at 60 °C. These were sealed with coverslips using LVR and incubated in an oven overnight at 60 °C. Subsequently, images of the semithin section series were attained with a Nikon NiU compound microscope equipped with a DsRi2. Pre-processing of images, such as conversion into grayscale, removing artefacts, and sorting were done with FIJI (Schindelin et al., 2012) and/or Adobe Photoshop (Adobe Inc.). Images were further processed in the 3D- reconstruction software Amira 6.3 (Thermo Fischer Scientific). Series of images were aligned with *Align Slices*, then structures of interest were labelled and segmented semi-manually in *Segmentation Editor* using the interpolate function in certain sections. Labelled structures were visualized with *Generating Surface* and adjusting the properties of the volume rendering *Volren* modules. A series of smoothing steps where used to improve the reconstructed surfaces with *Smooth Surfaces*. Images were obtained by taking snapshots of generated surfaces.

### Immunocytochemical staining and confocal laser scanning microscopy

The permeability of sample tissues was enhanced in a solution consisting of 2% Triton X-100 and 2% DMSO in phosphate buffer (PBT). They were placed on a rocking platform overnight at room temperature. Primary antibodies against acetylated alpha-tubulin raised in mice (Sigma Aldrich, St. Louis, MO, USA) were diluted in PBT (concentration of 1:800) and incubated for 24 h at room temperature. The next day, samples were washed in PB three times for 15 min each. Subsequently, samples were placed in a secondary antibody, raised in goat against mouse (AlexaFluor 568, Invitrogen, Carlsbad, CA, USA) with the fluorochrome in PBT (concentration of 1:300) for 24 h. Samples were washed in phosphate buffer three times for 15 min each. F-actin-labelling was achieved using the fluorescent stain AlexaFluor 488 phalloidin (Invitrogen, Carlsbad, CA, USA) diluted in PBT (concentration of 1:100). For staining cell nuclei, DAPI was added at a concentration of 1:100 in PBT. The samples in a combined solution of the secondary antibody, phalloidin, and DAPI were incubated in a darkroom overnight at room temperature. Thereafter, samples were rinsed three times for 20 min each in PB before they were mounted on microscope slides with Flouromount G (Southern Biotec, Birmingham, LA, USA). The slides were stored at 4 °C prior to investigation. Scanned images of samples were produced with a Leica SP5II confocal laser scanning microscope (Leica Microsystems, Wetzlar, Germany). Scanned images were visualized and analysed with the *Volume Rendering* module Amira version 6.3 (FEI, Oregon, USA). Images were obtained with the snapshot function.

### Casts

After images of shells with borings were taken, organic debris from shells was removed by submerging in household bleach for 48 h. Shells were rinsed with distilled water and ultrasonicated for 5 min, followed by drying in a fume hood for 24 h then in an oven for 2 h. Shells were embedded in Smooth-On Smooth-Cast 321™ Resin, preparation of the mixture was done according to manufacturer instructions. Shells were covered in a thin layer of the resin mixture. To ensure that the resin seeped into the bored holes, the treated shells were cured in a Heraeus vacuum oven (Heraeus Holding GmbH, Hanau, Germany) at 300 mbar for 40 min, then in an oven for 6 h at 60 °C. After the resin cast completely set it was placed in a 5% solution of hydrochloric acid to dissolve the calcium carbonate components of the shell. The casts were thoroughly rinsed with distilled water and allowed to dry in a fume hood for 24 h. Images of the cast colonies were produced with a Hirox-RH 2000 digital microscope system (Hirox Co., Ltd, Tokyo. Japan).

### Zooidal measurements

The size of the borehole aperture was determined by measuring its diameter at the widest point. The length of an autozooid was determined by measurements from the frontal to the basal end, along the longitudinal axis. The width was measured in the mid-zooidal region. Measurements were done with FIJI (Schindelin et al., 2012) and NIS Elements software (Nikon, Tokyo, Japan).

### DNA extraction

DNA of five samples was extracted. Prior to extraction zooids (between 2 and 20) were homogenized in a solution of lysis buffer and proteinase K and incubated overnight in a thermomixer at 56 °C, set to mix at 5-min intervals for 5 s at 300 rpm. To extract DNA, a QIAamp DNA Micro Kit (Qiagen) was used following the manufacturer’s protocol, sometimes with carrier RNA added to boost extraction as per protocol guidelines. The purity and amount of DNA extracted were determined using a NanoDrop™ 2000 spectrophotometer (Thermo Fisher Scientific, Wilmington, Delaware, USA). The mitochondrial Cytochrome c oxidase subunit 1 gene (COI) was amplified with PCRs in 30 µl reaction volumes consisting of 15 µl Biozym Red HS Taq Master Mix (Biozym Scientific GmbH, Hessisch Oldendorf, Germany), 0.5 µl forward and 0.5 µl reverse standard primers or primers specifically designed for boring bryozoans (Table S2), 14 µl MilliQ water and 2-3 µl DNA. PCR products were cleaned using an enzymatic clean-up reagent A’SAP (ArcticZymes Technologies ASA, Tromsø, Norway) and sent to Microsynth Austria GmbH for sequencing.

### Mitogenomes, sequencing, assembly, and annotation

Genomic DNA libraries were constructed using NEBNext^®^ Ultra™ II FS DNA Library Prep Kit for Illumina, with Imputs > 100 ng (# E7805) and NEBNext Multiplex Oligos for Illumina (Dual Index Primers, NEB #E7600) and sequenced on an Illumina NextSeq 550 platform using the 300 Cycle Mid Output mode. The library preparation and sequencing were conducted by the Next Generation Sequencing Facility at Vienna BioCenter core Facilities (VBCF), a member of the Vienna BioCenter (VBC).

Raw Illumina reads were first quality-checked with FastQC v0.11.8 (www.bioinformatics.babraham.ac.uk/projects/fastqc; last accessed March 16, 2023) and then were trimmed using Trim Galore v0.6.5 (https://github.com/FelixKrueger/TrimGalore; last accessed March 16, 2023) with default setting to remove adapters and low-quality sequences.

These reads were *de novo* assembled with SPAdes v3.15.3 (Bankevich et al., 2012) using k-mers of 21, 33, 55, 77, 99 and 127. The mitogenome of each sample was then identified using BLASTN (Altschul et al., 1990) and annotated with MITOS2 web server (Donath et al., 2019) using a metazoan reference (RefSeq 63) and the invertebrate genetic code. The annotated genes include two rRNA [mitochondrial large-subunit (16S) ribosomal RNA (rrnL) and mitochondrial small-subunit (12S) ribosomal RNA (rrnS)] and 13 protein coding genes (PCG) (*atp6*, *atp8*, *cox1*, *cox2, cox3*, *cob*, *nad1*, *nad2*, *nad3*, *nad4*, *nad4l*, *nad5*, and *nad6*).

Manual curation for these genes was performed using previously published mitogenomes of bryozoans available at NCBI as references. Two nuclear ribosomal operon genes (18S and 28S) were also identified and annotated using RNAamer (Lagesen et al., 2007). When the complete mitogenome was not recovered using this pipeline, exonerate (Slater & Birney, 2005) with affine:local model and maximum intron length set to 40 kb was used to scan the whole genome assemblies to identify the missing mitochondrial genes.

### Sequence processing and phylogenetic analyses

The phylogenetic relationships among immergentiids were inferred based on a concatenated dataset of 16 genes including twelve PCG (*atp6*, *cox1*, *cox2, cox3*, *cob*, *nad1*, *nad2*, *nad3*, *nad4*, *nad4l*, *nad5* and *nad6*), two ribosomal rRNA genes (12S and 16S) and two nuclear ribosomal operon genes (18S and 28S). In order to construct this dataset, MAFFT v7.490 (Katoh & Standley, 2013) with the following parameters: auto, localpair, maxiterate 1000, was first used to align these genes, and then the alignment of each gene was trimmed to remove ambiguous characters using BMGE v. 1.12.2 (Criscuolo & Gribaldo, 2010) for PCG and trimAl v1.4. rev15 (Capella-Gutiérrez et al., 2009) with gt 0.6 and some manual adjustments for the ribosomal genes (12S, 16S, 18S, and 28S). The single gene alignments were concatenated to a supermatrix using AMAS (Borowiec, 2016).

Phylogenetic trees were inferred using Bayesian inference (BI) and maximum likelihood (ML) methods based on a partitioned data matrix. ModelTest-NG (Darriba et al., 2020) was used to estimate the best-fitting evolutionary model for each locus based on the corrected Akaike Information Criterion. The general time reversible model (GTR+I+G4) was selected as the best model for the ribosomal genes, and (MTZOA+G4+FC) was the best model for PCG. The ML tree was constructed using RAxML-NG v. 1. 0. 2 (Kozlov et al., 2019) with a best-fitting model for each locus as determined by ModelTest-NG. Topological support was assessed with 1000 bootstrapping. BI analysis was performed using mrbayes5D 3.2.6, a modified version of MrBayes 3.1.2 (Huelsenbeck & Ronquist, 2001) incorporating the MtZoa evolutionary model, with two separate runs of four chains of a Markov chain Monte Carlo (MCMC) algorithm running for five million generations and tree sampling occurring every 100 generations. The best-fitting model for each locus was used as determined by ModelTest-NG above. Convergence of the Bayesian runs was assessed by checking the average deviation of split frequencies (ADSF) (the runs had to have values of ASDF approaching zero) and by inspection of the tracefile outputs in Tracer (Nascimento et al., 2017). The first 25% of samples were discarded as burn-in, and the remaining trees were used to calculate posterior probability values and to build the consensus tree. Figtree v1.4.4 (http://tree.bio.ed.ac.uk/software/figtree/) was used to visualize the final ML and BI trees.

### Genetic distance

ML-corrected substitutions per site were calculated in MEGA 7 using the maximum composite likelihood parameter with a gamma parameter of 1.0 (Kumar et al., 2008; Tamura et al., 2004, 2021).

### Terminology

To ensure harmony for terms related to zooidal body axis, ‘frontal’ refers to the apertural area, and ‘basal’ refers to the opposite tip (see Schwaha, 2020a; Decker et al., 2023). Similarly, the terms ‘distal’ and ‘proximal’ are used to describe orientation within the polypide. Besides, after assessing characteristics of zooids, it became clear that different authors used various terms to refer to the same structures, which could cause confusion in interpretation. In this study, we consider cystid appendages as extensions of the cystid wall. Here, cystid appendages that are typically positioned at the apertural area of a zooid are called primary cystid appendages (pcy), and those at the mid-zooidal area of a zooid are called secondary cystid appendages (scy). A list of terms (and their translations where applicable) that were commonly used to refer to the primary and secondary cystid appendages as they relate to recent immergentiid morphology was compiled (Table S3). The terms used for *I. philippinensis* are omitted here (see Discussion). The term intercalary kenozooid is introduced and defined as a kenozooidal tube derived from the cystid wall and inserts between neighbouring cystid appendages.

## Results

### Systematics, emended diagnoses, and species details

Class Gymnolaemata Allman, 1856

Order Ctenostomata Busk, 1852

Superfamily Arachnidioidea Hincks, 1880

Family Immergentiidae, Silén, 1946

Genus *Immergentia*, Silén, 1946

**Emended diagnosis:** Non-pedunculate boring bryozoans, living in shells of molluscs. Colony typically enantiomorph, with alternating left-right symmetries of zooids. Autozooids vase shaped, slightly asymmetrical, with a tapered or rounded basal tip slightly bent in direction to shell surface. Zooids vertically or obliquely oriented in shell. Primary cystid appendages (pcy) from pointed both ends of zooidal aperture. Secondary cystid appendages (scy) in mid-region of zooids. Additional cystid anastomoses present at various locations of autozooid. Polypide with 8–10 tentacles, gut with distinct cardiac constrictor, and bulbous caecum. Low- to mid-lophophoral anus. Intercalary kenozooids connecting primary or secondary cystid appendages. Sac zooids bulb- or vase-shaped and typically 1/3 to 2/3 the size of an autozooid. Single embryos brooded, developing in degenerated zooid.

**Remarks:** Silén (1946) described the aperture as regularly quadrangular with a thickened cuticle at distal end. Though borehole apertures are typically spindle-shaped, resembling a bulged ‘S’ shape, they can also appear as oval or circular. Cystid appendages refer to thin extensions of the zooid itself, referred to as connecting prolongations in the original diagnosis of the genus (Silén, 1946) and subsequently stolo-like threads (Silén, 1947).

Amendments to the genus now include the presence of two types of kenozooids found in a colony. First, intercalary kenozooids that connect primary or secondary cystid appendages. The second type of kenozooids is sac zooids separated from a connecting stolon or cystid appendage by a septum.

Though Silén (1947) first reported brooding in immergentiids, this was not included in the initial diagnosis of the genus. Soule (1950) then added this to his generic description. The development of an embryo within the tentacle sheath of a degenerated zooid is confirmed here.

Based on samples examined and literature, a minimum of eight and a maximum of ten tentacles have been reported. Additionally, analysis of the soft-body morphology revealed the presence of a cardiac constrictor in the gut not reported before. The anus is typically located in the low- to mid-lophophoral position of the polypide.

### Analysed species

***Immergentia stephanieae*** Johnson & Schwaha sp. nov.

Figures 1, 2, 3d, f, 4, 5, 6, 7 and 8i

**Fig. 1:**
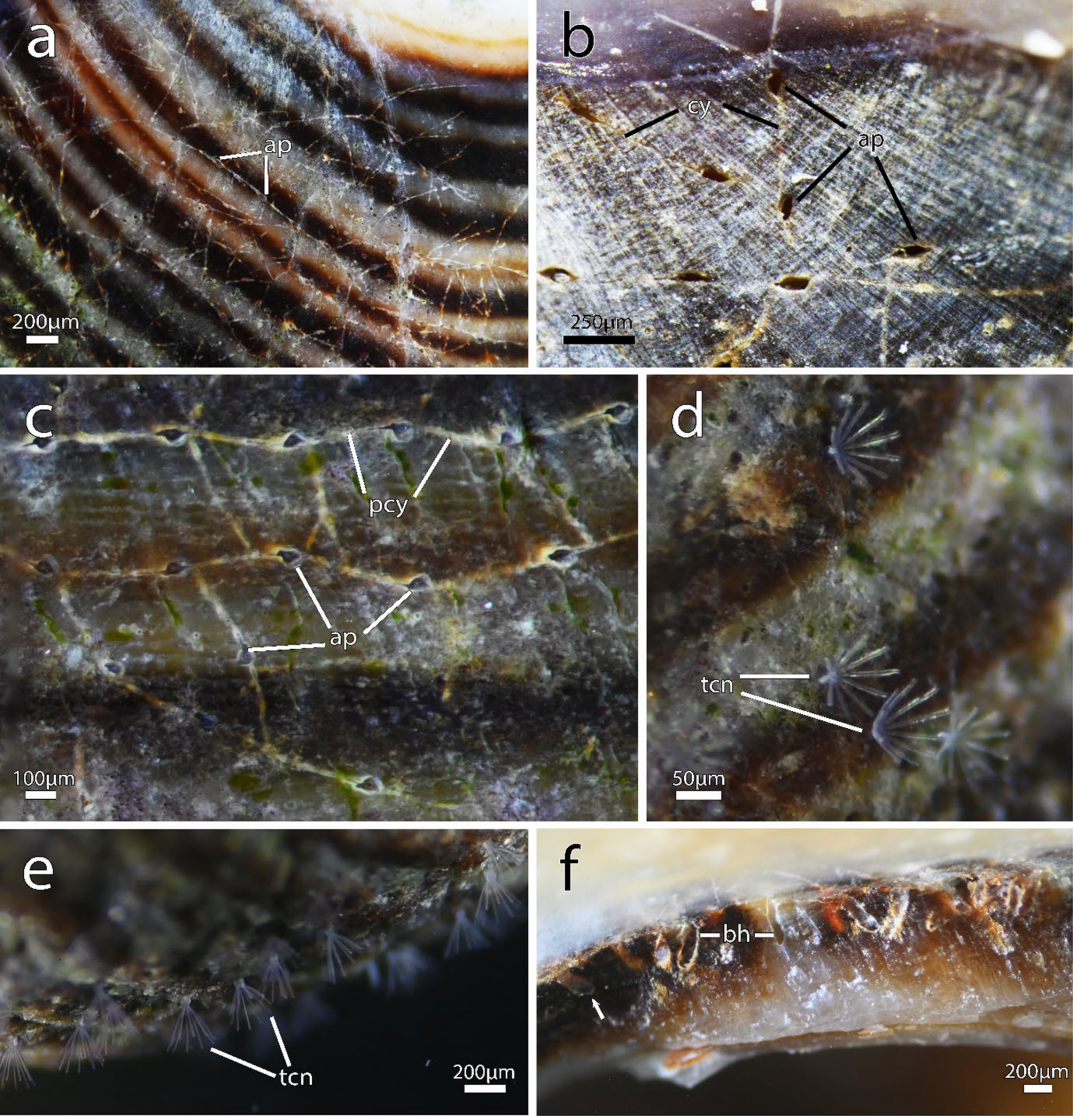
*Immergentia stephanieae* sp. nov (Type locality: Roscoff, France; MNHN-IB-2017-721). **a** Living gastropod shell heavly bored by the immergentiid. Regular arragement of colony with interconnecting cystid appendages. **b** Close up of the borehole apertures and cystid appendages connecting neighboring zooids. **c** Typical branching pattern of regularly spaced colony. Enantiomorphic colony structure. **d** Tentacle crowns of autozooids. **e** Tentacle crowns extended in a dense colony. **f** Lateral view of a broken shell showing elongated boreholes. Remnants of the soft tissue (whitish) are visible in some boreholes (arrow). Abbreviations: ap – aperture, bh – borehole, pcy – primary cystid appendage, tcn – tentacle crown

**Fig. 2:**
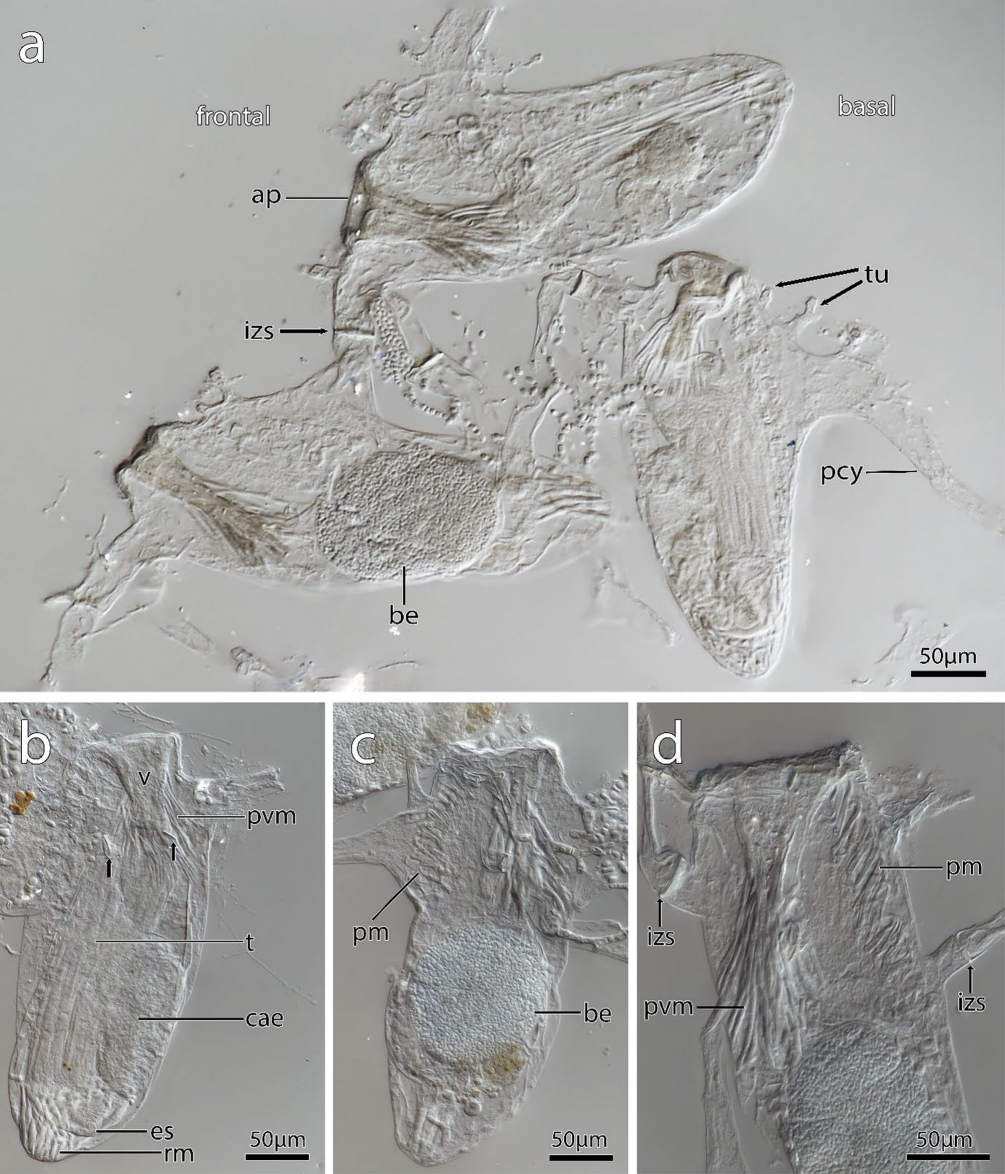
Zooidal plasticity of *Immergentia stephanieae* sp. nov**.** (Type locality: Roscoff, France; MNHN-IB-2017-721) Zooids after shell decalcification.** a** Degenerated zooid (top) with putative developing embryo. Reproductive zooid with brooded embryo on the left and functional autozooid on the right. Tubulets extend to the substrate surface in the frontal area. **b** Typical vase shape of zooids. Autozooid with lophophore, digestive tract, body wall and apertural muscles. Interzooidal septa extend frontally in different plane (arrows). **c** Reproductive zooid with brooded embryo. **d** Distal apertural and mid-zooidal area. Parieto-vestibular muscles in the apertural area. Parietal muscles that attach to the body wall. Cystid appendages with emphasis on interzooidal septum. Abbreviations: ap – aperture, be – brooded embryo, cae – caecum, es – oesophagus, izs – interzooidal septum, pcy – primary cystid appendage, pm – parietal muscles, pvm – parieto-vestibular muscles, rm – retractor muscle, t – tentacles, tu – tubulets, v – vestibulum

**Fig. 3:**
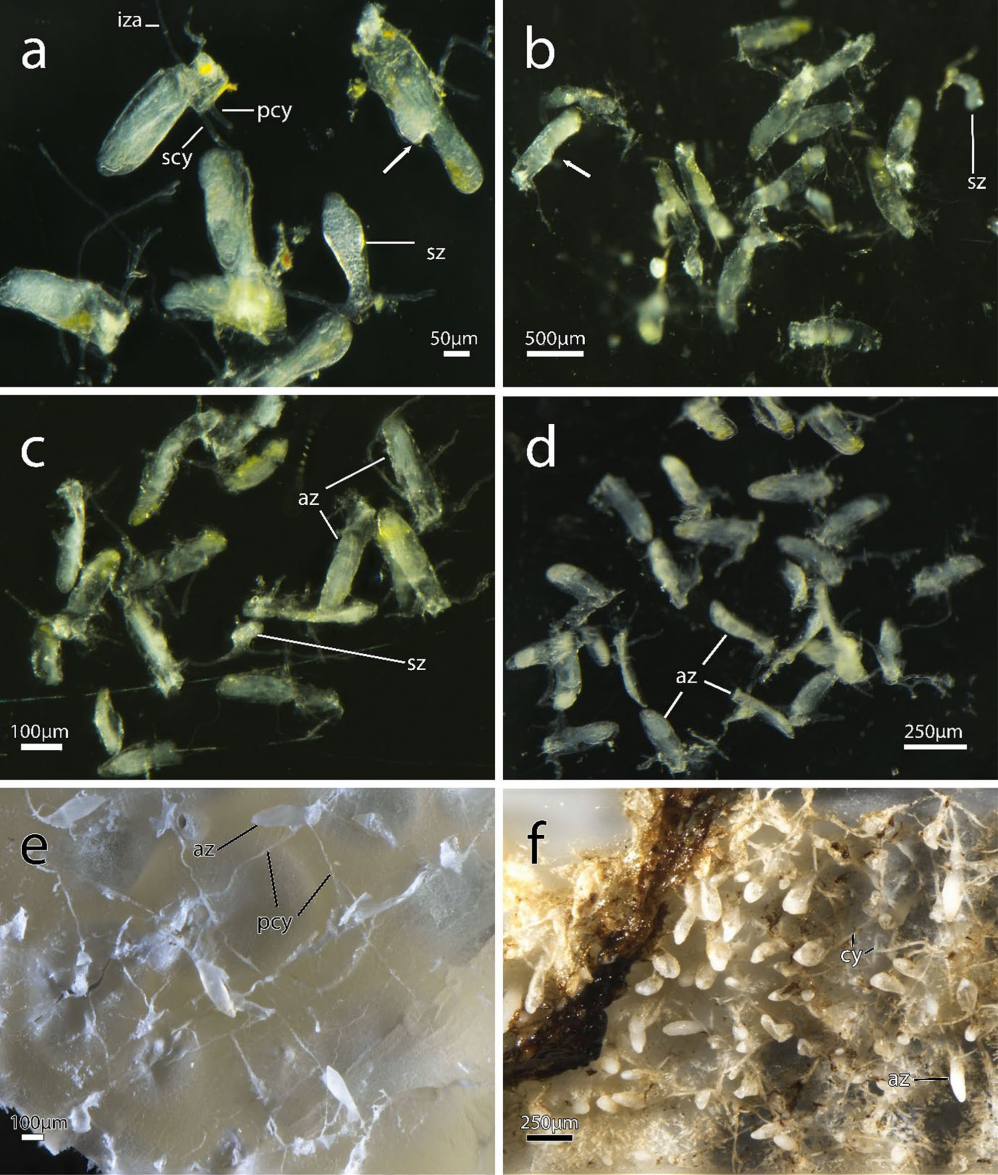
Zooidal plasticity of *Immergentia*. **a–d** Zooids of immergentiids after decalcification of substrate. **a** Autozooids and sac zooid of *Immergentia* cf. *zelandica* (Locality: Inner Otago shelf, New Zealand). Zooid with slightly narrowed mid-zooidal region and elongated rounded basal end (arrow). **b** Autozooids and sac zooid of *Immergentia pohowskii* sp. nov. (Locality: Taiaroa Head, New Zealand). Several zooids with slightly narrowed mid- region (arrow).** c** Autozooids of *Immergentia* cf. *suecica* from France.** d** Zooidal plasticity of *Immergentia stephanieae* sp. nov (Locality: Roscoff, France). **e** Resin casts of zooids of subtidal *Immergentia* cf. *suecica* from Roscoff, France. Primary cystid appendage connect sparely spaced zooids. **f** Resin casts of dense colony of *Immergentia stephanieae* sp. nov. Abbreviations: az – autozooid, iza – interzooidal anastomoses, pcy – primary cystid appendage, scy – secondary cystid appendage, sz – sac zooid

**Fig. 4:**
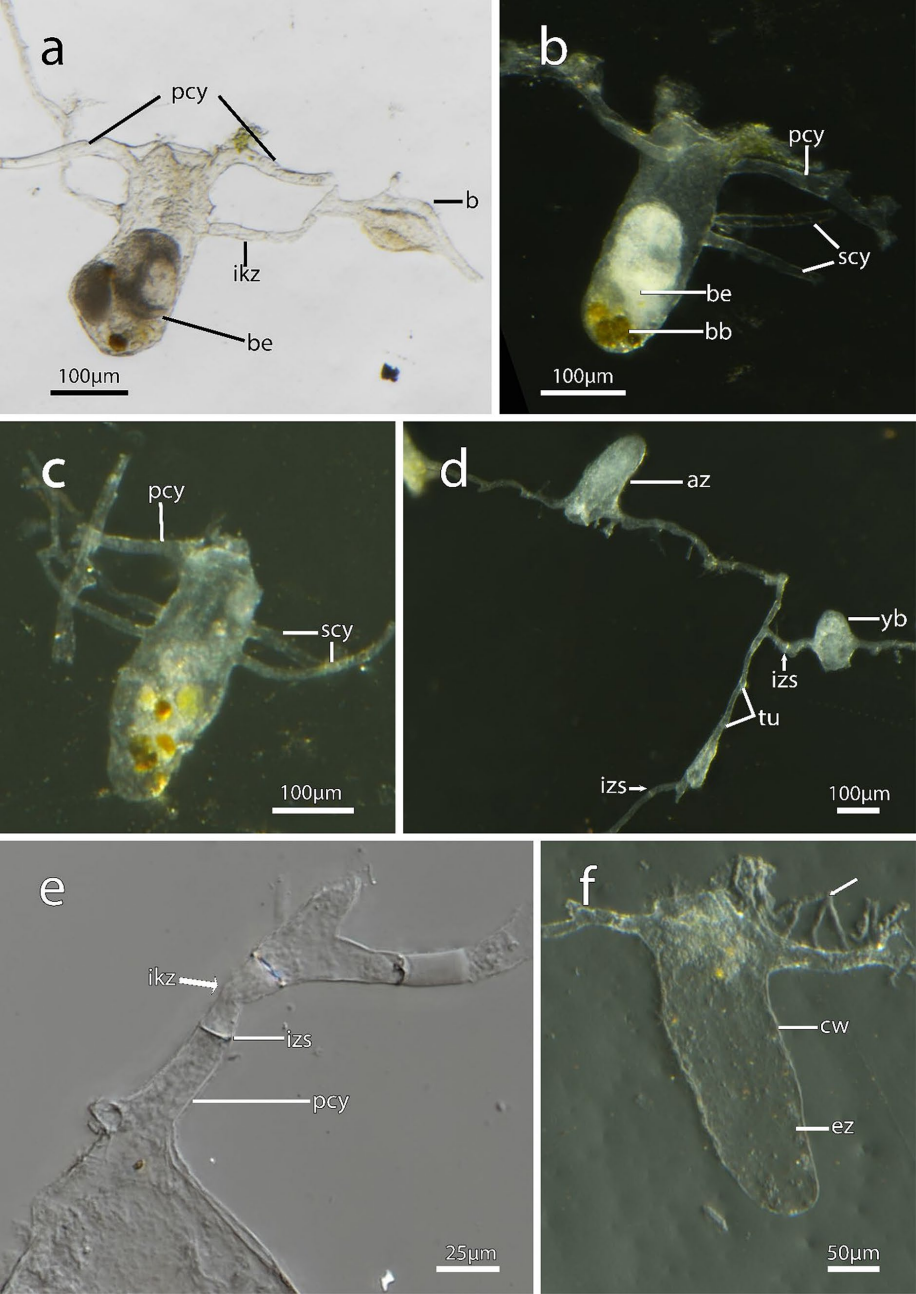
Cystid appendages and intercalary kenozooids in *Immergentia stephanieae* sp. nov (Locality: Roscoff, France). Zooids after shell decalcification. **a** Reproductive zooid connected to cystid appendage of a developing bud by an intercalary kenozooid. Note: thickened cuticle of developing bud’s apertural area. **b** Reproductive zooid. Degenerated zooid with brooded embryo and two secondary cystid appendages emerging from the proximo-distal axis of the zooidal cytid wall. **c** Degenerated zooid with one primary cystid appendage and four secondary cystid appendages. **d** Cystid appendage with tubulets and interzooidal septa, young bud on the right. **e** Intercalary kenozooid wedged between cystid appendages. **f** Empty zooid with thin processes (arrow) emerging from the primary cystid appendage. Abbreviations: az – autozooid, b – bud, be – brooded embryo, bb – brown body, cw – cystid wall, ez – empty zooid, ikz – intercalary kenozooid, izs – interzooidal septum, pcy – primary cystid appendage, scy – secondary cystid appendage, tu – tubulets, yb – young bud

**Fig. 5:**
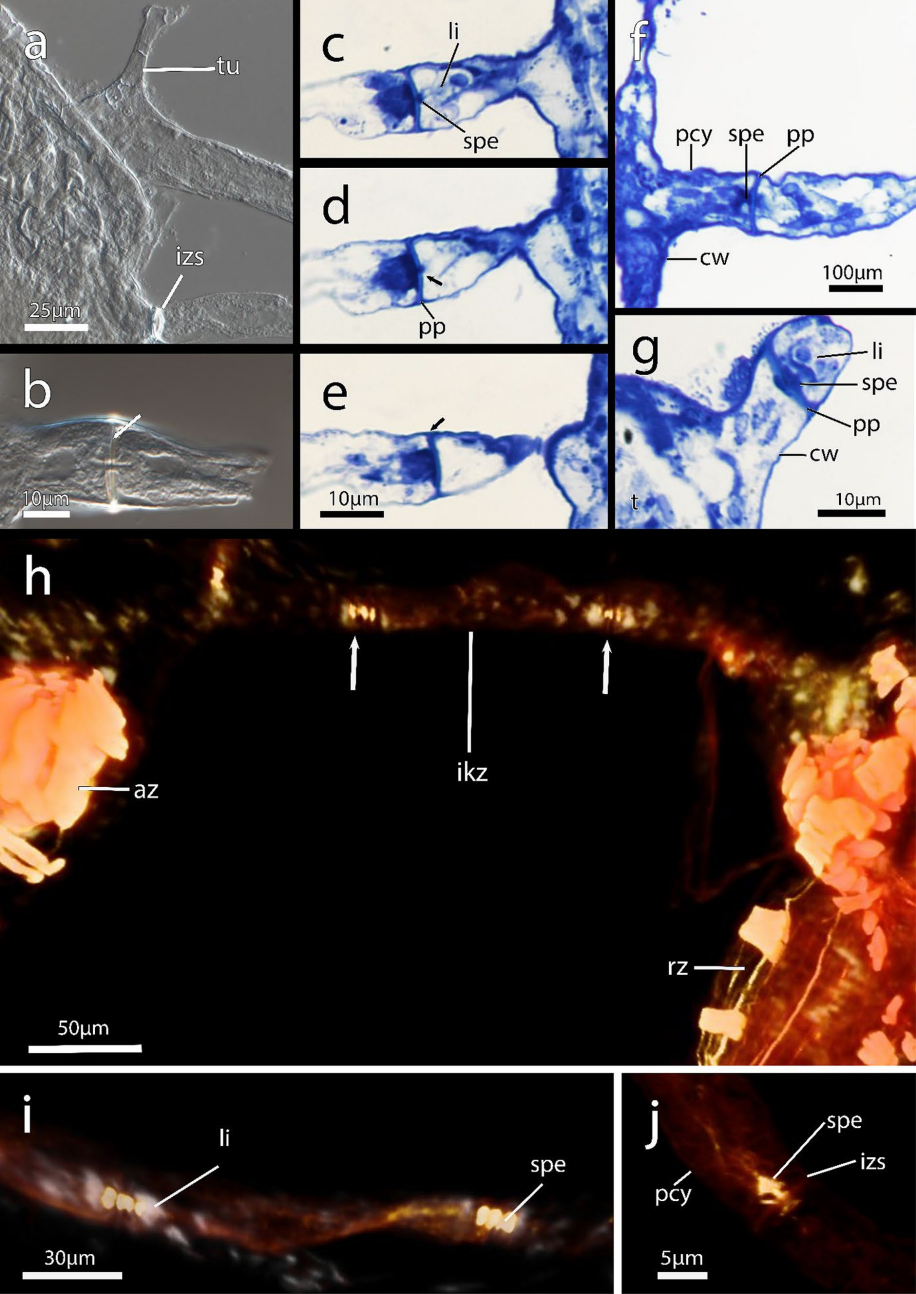
Interzooidal communication pores in *Immergentia stephanieae* sp. nov. (Locality: Roscoff, France) from whole mounts, semi-thin histological sections and volume rendering of confocal scans. **a** Cystid appendage bearing an interzooidal septum and tubulet from whole mount. **b** Interzooidal spetum with single pore. **c**–**e** Series of interzooidal septa on primary cystid appendage.** c** Special cell and limiting cells within the pore plate.** d** Single communication pore (arrow) in pore plate. **e** Interzooidal septum (arrow). **f** Pore plate complex within the primary cystid appendage in the distal apertural area. **g** Pore plate complex in sunken primary cystid appendage located distal of the tentacle sheath. **h** Volume rendering of neighboring zooids separated by a an intercalary kenozooid. Interzooidal septum indicated by arrows. Autozooid on the left and degenerated zooid with brooded embryo on the right. **i** Close up of an intercalary kenozooid between cystid appendages. **j** Interzooidal septum at junction between adjacent primary cystid appendages. Abbreviations: az – autozooid, cw – cystid wall, ikz – intercalary kenozooid, izs – interzooidal septum, li – putative limiting cell, pcy – primary cystid appendage, pp – pore plate, rz – reproductive zooid, spe – special cell

**Fig. 6:**
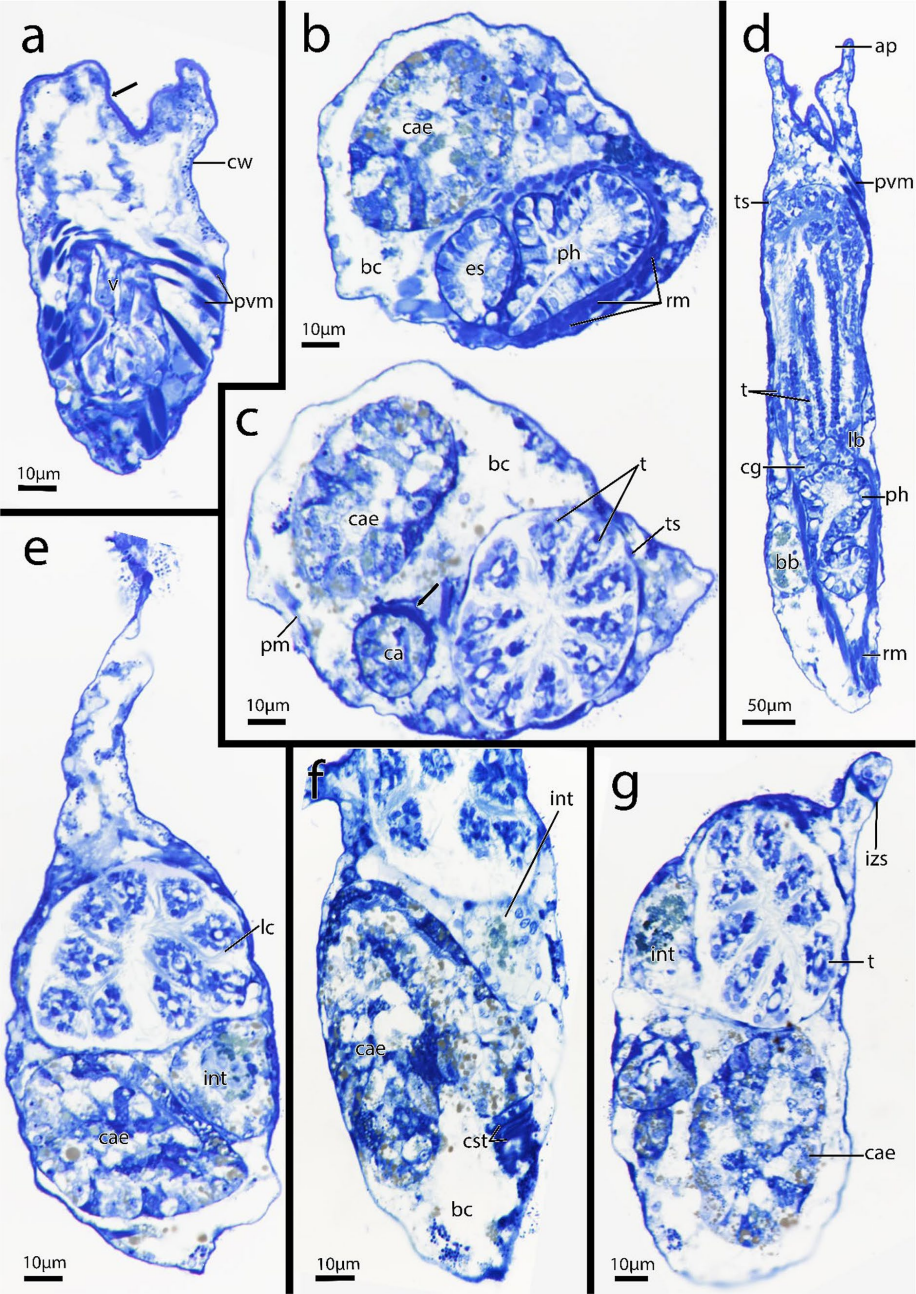
Semi-thin histological serial sections of *Immergentia stephanieae* sp. nov. from Roscoff, France. **a** Apertural area of zooid (arrow) vestibulum and parieto-vestibular muscles. **b** Transition of the pharynx to oesophagus. Retractor muscles and caecum are visible. **c** Autozooids with ten tentacles. Transition into mid-gut. Arrow indicates circular muscles of cardiac constrictor. **d** Longitutinal section of autozooid. Cerebral ganglion at lophophoral base.** e** Same zooid as b & c. Caecum and intestine. Lateral cilia of tentacles.** f** Circular muscles of cardiac constrictor. **g** Autozooid with nine tentacles. Abbreviations: ap – aperture, bb – brown body, bc – body cavity, cae – caecum, cg – cerebral ganglion, cst – cardiac constrictor, cw – cystid wall, es – oesophagus, int – intestine, izs – interzooidal septum, lb- lophophore base, lc – lateral cilia, ph—pharynx, pm – parietal muscles, pvm – parieto-vestibular muscles, rm – retractor muscle, t – tentacles, ts – tentacle sheath, v – vestibulum

**Fig. 7:**
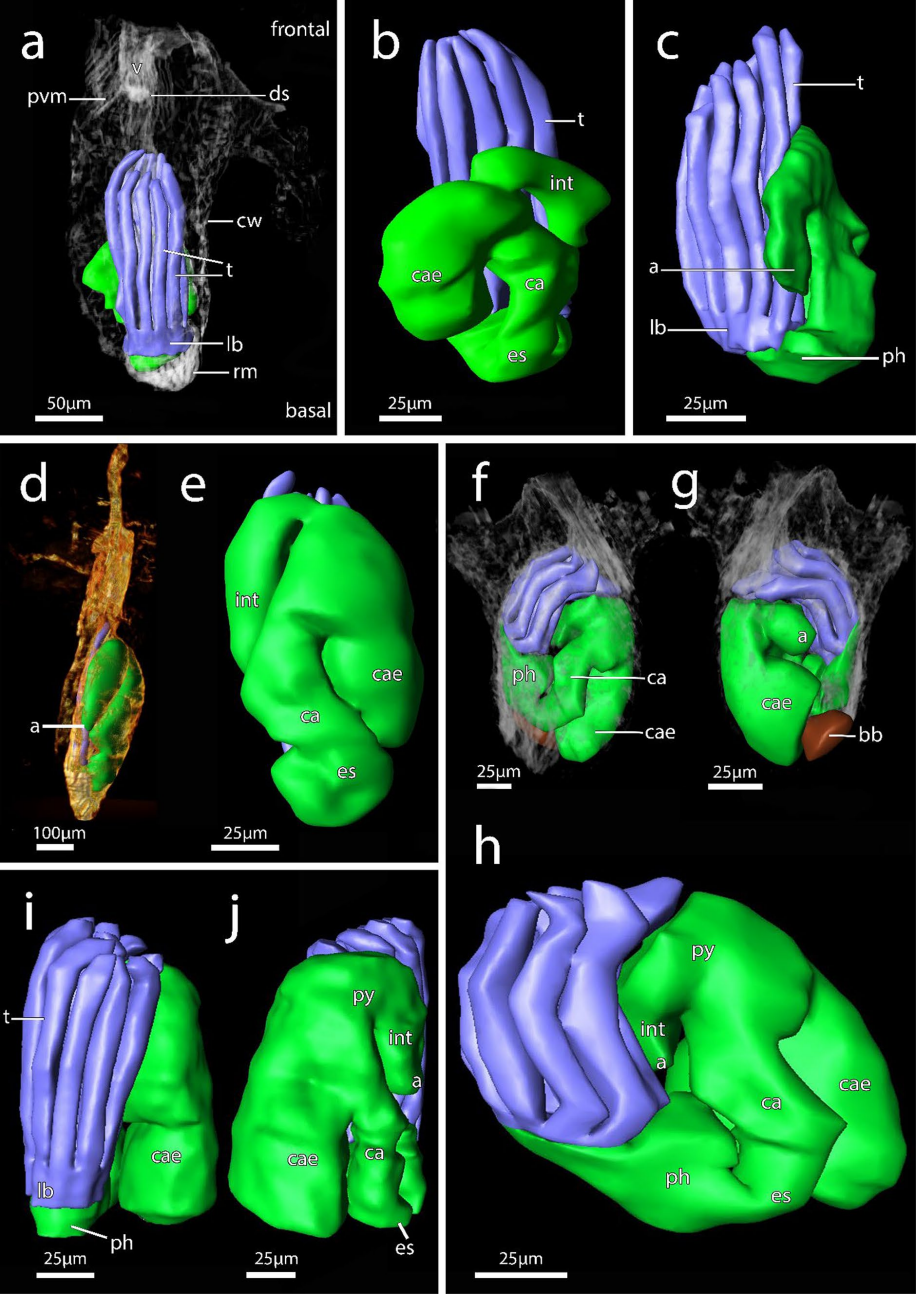
3D reconstruction of immergentiid polypides from histological serial sections. **a–b** Overview of autozooids of *Immergentia patagoniana* (Locality: Burdwood Bank, south-western Atlantic). **a** Oral view of polypide. **b** Close up of typical digestive tract shape.The low positioned lophophoral anus retracted tentacle sheath. **c** Side view of lophophore and digestive tract of *Immergentia* cf. *suecica* (Locality: Roscoff, France). **d** Autozooid of *Immergentia* cf. *suecica* from Trodheim Fjord Norway. **e** Close up of digestive tract of the same autozooid **f**–**h** Overview of autozooid and digestive tract of *Immergentia stephanieae* sp.nov. (Locality: Roscoff, France) with 9 tentacles. **f** Orientation of lophophore and gut in the zooid, oral view. **g** Same zooid, anal view of digestive tract, with brown body proximal to caecum. The anus terminates in tentacle sheath close to lophophoral base. **h** Digestive tract of same zooid. Foregut consist of the mouth opening, pharynx and elongated oesophagus. Midgut comprises the cardia, caecum and pylorus. The hindgut is comprised of the intestine and anus. **i** Oral side of lophophore indicating pharynx, lophophore base and tentacles in *Immergentia stephanieae* sp. nov. with 10 tentacles.** j** Anal view of the same autozooid. Abbreviations: a – anus, bb – brown body, ca – cardia, cae – caecum, cw – cystid wall, ds – diaphragmatic sphincter, es – oesophagus, int – intestine, lb – lophophore base, ph – pharynx, pm – parietal muscles, py – pylorus, rm – retractor muscle, t – tentacles, v – vestibulum

**Fig. 8:**
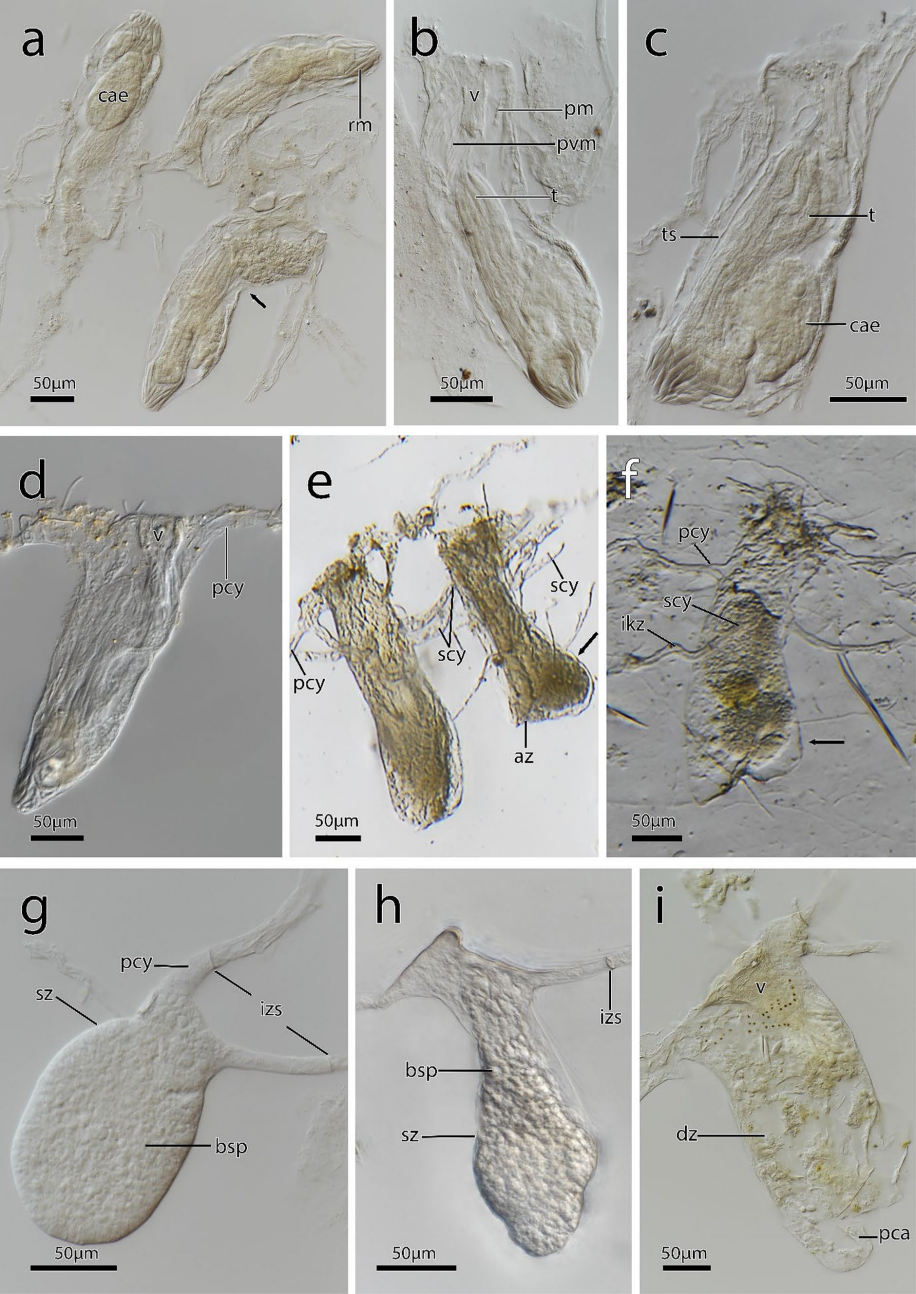
Autozooids and heterozooids in *Immergentia* from whole mounts after shell decalcification. **a** Autozooids of *Immergentia patagoniana*, from Burdwood Bank, south-western Atlantic Ocean, with two zooids distinct curvature in the mid-zooidal region (indicated by arrow). **b** Close up of autozooid from the same colony as in a. Vestibulum and parieto-vestibular muscles with bulge caused by caecum in *Immergentia patagoniana*. **c**. Large caecum in the proximal region of *Immergentia patagoniana*. **d** Autozooid of *Immergentia* cf. *suecica* from Roscoff, France. Tapered basal tip and slanted zooid. **e** Autozooids of *Immergentia* cf. *zelandica* from Inner Otago shelf, New Zealand, with primary and secondary cystid appendages. Bulge caused by digestive tract (arrow).** f** A reproductive zooid of *Immergentia* cf. *zelandica* with primary and secondary cystid appendages and an intercalary kenozooid. Arrow points to bulge of cystid wall. **g** Bulb shaped sac zooid *Immergentia patagoniana* with cystid appandages and interzooidal septa. Birefringent spherules fill the zooid. **h** Vase-shaped sac zooid *Immergentia* cf*. zelandica*. **i** Degenerating zooid of *Immergentia stephanieae* sp. nov with a proximal cyctid appendage from Roscoff, France. Abbreviations: az – autozooid, bsp – birefringent spherules, cae – caecum, dz – degenerated zooid, ikz – intercalary kenozooid, izs – interzooidal septum, pca – proximal cystid appendage, pcy – primary cystid appendage, pm – parietal muscles, pvm – parieto-vestibular muscles, rm – retractor muscle, scy – secondary cystid appendage, sz – sac zooid, t – tentacles, ts – tentacle sheath, v – vestibulum

**Material examined. Holotype**: NORTH-EAST ATLANTIC • zooids from colony in absolute ethanol; France, Roscoff, west of the Marine Research Station; 48° 43.698' N, 03° 59.721' W; depth: intertidal; 20 September 2021; M. Johnson leg.; immergentiid colonies in gastropod *Littorina littorea* Linnaeus, 1758, from tide pools and under rocks, boulders and *Fucus* Linnaeus, 1753, blades; ZooBank: urn:lsid:zoobank.org:act:3842DCC5-9F7A-4FDE-A1AB-99592DBF255F; GenBank: SAMN38786230; MNHN-IB-2017-721.

**Paratypes**: NORTH-EAST ATLANTIC • slide, whole mount; same collection data as for holotype; MNHN-IB-2017-722 • slide, serial sections; same collection data as for holotype; MNHN-IM-2022-13772 • two dried shells of the gastropod *L. littorea* (Table 1) labelled 1 and 2 with borings of *Immergentia stephanieae* sp. nov.; MNHN-IM-2022-13772.

**Table 1:** Substrates of molluscs bearing borings of *Immergentidae* reported from type/paratype material and examined in this study

***Immergentia*** **species**	**Locality**	**Substrate**	**Reference**	**Comments**
*I. patagoniana*	Burdwood Bank	*Argeneuthria cerealis* de Rochebrune & Mabille, 1885	This study	
	Burdwood Bank	*Argeneuthria paessleri* Strebel, 1905	This study	
	Patagonia, Argentina	*Buccinanops cochlidium* Dillwyn, 1817	Pohowsky (1978)*	
	Burdwood Bank	*Pareuthria atrata* Smith, 1881	This study	
	Burdwood Bank	*Pareuthria fuscata* Bruguière, 1789	López-Gappa and Zelaya (2021), this study	*Pareuthria plumbea* (Philippi, 1808) in López-Gappa (1981)
	Burdwood Bank	*Savatiera chordata* Castellanos, Rolán & Bartolotta, 1987	This study	
	Burdwood Bank	*Trophon ohlini* Strebel, 1904	This study	
	Burdwood Bank	*Typhlodaphne* sp.	This study	
*I. pohowskii* sp. nov.	Subtidal Taiaroa Head	Unnamed molluscan shell fragment	This study	Only fragments of molluscan shells, species could not be determined
*I. stephanieae* sp. nov.	Roscoff intertidal zone	*Littorina littorea* Linnaeus, 1758	This study	
	Roscoff intertidal zone	*Nucella lapillus* Linnaeus, 1758	This study	
*I. suecica*	Gullmar Fjord, West Coast of Sweden	*Pseudamussium peslutrae* Linnaeus, 1771	Silén (1947)*	*Pecten septemradiata* in Silén (1947)
*I.* cf. *suecica* France	Roscoff: Stolvezen, Térenéz, Chateaux du Taureau	*Anomia* sp. Linnaeus, 1758	This study	
		*Ensis* sp. Schumacher, 1817	This study	
		*Glycymeris glycymeris* Linnaeus, 1758	This study	
		*Lutraria lutraria* Linnaeus, 1758	This study	
		*Pecten maximus* Linnaeus, 1758	This study	
*I.* cf. *suecica* Norway	Trondheim Fjord	*Buccinum undatum* Linnaeus, 1758	This study	
*I. zelandica*	Slipper Island, New Zealand,	*Buccinulum littorinoides* Reeve, 1846	Silén (1946)*	*Euthria strebeli* in Silén (1946)
*I.* cf*. zelandica*	Otago mid shelf, Otago inner shelf	Buccinoidea Rafinesque, 1815	This study	Fragments of shells, molluscan species could not be determined. One gastropod
*I. zelandica* var* minuta*	Hawaiian Islands	*Conus striatus* Linnaeus, 1758	Soule and Soule (1969)*	
	Hawaiian Islands	*Cypraea* sp.? (Linnaeus, 1758)	Soule and Soule (1969)	
	Zamboanga, Philippine Islands	*Stomatella planulata* Lamarck, 1816		*Gena planulata* in Soule (1950)

Substrate species names according to WoRMS classification. Authority of immergentiid species indicated by (*)

**Additional material**. University of Vienna Biology Building, Vienna, Austria. Same data as holotype - specimen numbers: FR21-A61gas1 (whole mount), FR21GP59_G14 (histological serial sections), FR21-61 (absolute ethanol). Additional specimen collected from Roscoff were examined: • immergentiids in *L. littorea* and *Nucella lapillus *Linnaeus, 1758; France, Roscoff, around the Marine Research Station; depth: intertidal; 16 July 2019; T. Schwaha leg.; FR19-30F, FR19-50F, FR19-30F, FR19-50F• immergentiids in *L. littorea*; France, Roscoff; depth: intertidal; September 2020; T. Schwaha leg.; FR20-59FP, FR20-55A, FR20-56A, • immergentiids in *L. littorea*; France, Roscoff, Santec, 48° 42.787' N 4° 1.315' W; depth: intertidal; 2 September 2021; M. Johnson leg.; FR21–A3, FR21–G3, • immergentiids in *L. littorea* and *N. lapillus*; same location as type; M. Johnson leg.; FR21–A17, FR21GP59, dried shells. FR21-GRYI •immergentiids in *L. littorea*; France, Carantec, Île Callot; depth: intertidal; 3 October 2021; S. Decker leg.; FR21–A20.

**Etymology**: The species is named in honour of Stéphanie Cabioch, captain of the *Neomysis* research vessel of the Station Biologique de Roscoff.

**Diagnosis**: Endolithic bryozoan with borehole apertures densely packed, occurring at regular intervals in straight rows at the angle/direction of growth (Fig. 1c). Opposite/paired branching of lateral intercalary kenozooids/cystid appendages from the primary cystid appendage (pcy) can occur.

**Description**: Colony dense with zooids regularly spaced (Fig. 1a–f) typically in straight rows, average interzooidal aperture intervals 292 ± 83 µm. Borehole apertures oval (Fig. 1b) or spindle shaped (Fig. 1c), sometimes one end with rounded nudge and enantiomorphic with an average width of 66 ± 27 µm. Zooids vase shaped (Fig. 2), length 345 ± 23 µm and width of 129 ± 19 µm, with a rounded or slightly tapered basal tip (Fig. 3d, f), almost vertically placed in substrate with basal end curved toward primary cystid appendage (Figs. 1f and 4). Up to four secondary cystid appendages in mid-region of zooids (Fig. 4c). Neighbouring zooids are connected by an interzooidal septum (Fig. 5). Tubulets may be present or absent from the primary cystid appendage (Figs. 2a, 4d and 5a). Additional thin processes may also extend toward the substrate surface from the primary cystid appendage (Fig. 4f). Polypide with 9 or 10 tentacles (Figs. 1d,e and 6c, g), low positioned lophophoral anus (Fig. 7f–h). A proximal cystid appendage may also occur, though rare (Fig. 8i). Intercalary kenozooids connect primary or secondary cystid appendages. Sac zooids bulb or vase shaped, overall 1/3 to 2/3 of autozooid size. Degenerated autozooid (devoid of tentacles and digestive tract) with single embryo brooded within the tentacle sheath, referred to as reproductive zooid (Figs. 2a, c and 4a, b).

**Remarks**: Based on current information, *I. stephanieae* sp. nov. is the only immergentiid with either 9 or 10 tentacles and equal chances of encountering such polypides in a colony (Fig. 6c, e, g). The lophophoral anus terminates distal of the lophophoral base (Fig. 7f, g, h, j). Some colonies possess borehole apertures that are larger than the average size with a mean width of 104 µm. In such individuals the zooids appear stubby, and the distal end of the lophophore is the same height as the most distal part of the digestive tract (Fig. 7f–h). In the most commonly encountered zooids, typical for the genus, the distal part of the digestive tract only reaches about mid-way of the lophophore in the proximal-distal direction. The colony form in *I. stephanieae* sp. nov*.* is unique and forms a straight line at regular intervals. When there is a change in direction of the primary cystid appendage, a secondary cystid appendage may emerge in the opposite direction (Fig. 1a–c).

**Fig. 9:**
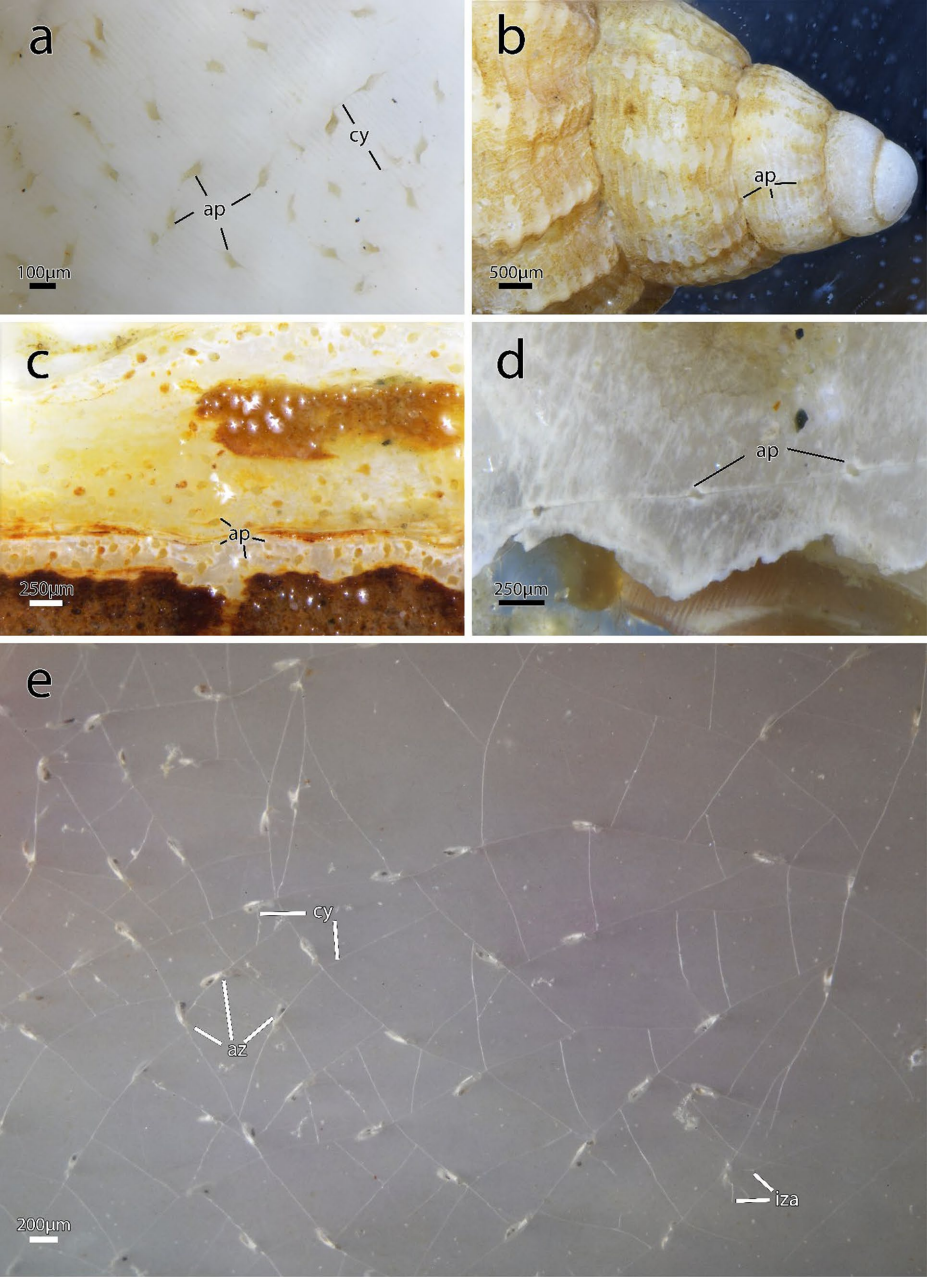
Four *Immergentia* species from five different locations. **a** Strongly enantiomorphic spindle shaped borehole apertures of *Immergentia patagoniana* from Burdwood Bank, south-western Atlantic Ocean). Cystid appendages connecting zooids visible. **b** Branching pattern in colony of *Immergentia* cf. *suecica* from Norway.** c** Oval shaped apertures of *Immergentia* cf. *zelandica* from the Inner Otago shelf, New Zealand. **d** Enantiomorphic spindle shaped apertures of *Immergentia pohowskii* sp. nov. from Taiaroa Head, New Zealand. **e** Branching pattern in dense colony of *Immergentia* cf. *suecica* from Roscoff, France. Apertures, cystid appendages connect neigboring zooids and entire zooids visible in transparent shell. Developing interzooidal anastomoses. Abbreviations: ap – aperture, az – autozooid, cy – cystid appendage, iza – interzooidal anastomoses

The colony morphology of *I. stephanieae* sp. nov. is described as regularly spaced and differs from the type *I. californica* where zooids are arranged in straight rows but opposite branching of lateral intercalary kenozooids/cystid appendages does not occur (see Silén, 1947, p. 41, fig. 58). The later could not be confirmed with confidence based on borings in the type material (Fig. S1). In *I. suecica*, zooids also generally occur in straight rows (see Silén, 1947, p. 41, fig. 60; *I.* cf. *suecica* Fig. 9b); however, zooids and lateral intercalary kenozooids are irregularly and sparsely spaced (*I.* cf. *suecica* Figs. 9e and S1). The interzooidal spaces of *I. stephanieae* sp. nov. are densely packed, only about one-third (in length) compared to *I.* cf. *suecica* from the subtidal zone of France (917 ± 175 µm) and Norway (952 ± 365 µm; see Table S4). Though zooids of *I. patagoniana* are also densely packed, they are easily distinguishable from *I. stephanieae* sp. nov., because their borehole apertures are elongated, strongly enantiomorphic, relatively narrower, and almost half the size (Fig. 9a) while borehole apertures of *I. stephanieae* sp. nov. range from oval to spindle shaped and are only weakly enantiomorphic (Fig. 1a–c).

In addition to the density and the variation of borehole apertures, the zooids of *I. patagoniana* differ from *I. stephanieae* sp. nov. with zooids that may be strongly curved in the mid-zooidal region and pointed toward the pcy. Similarly, zooids of *I. angulata* and *I. subangulata* are strongly curved in the mid-zooidal region and pointed toward the pcy but are widely spaced (Table S5). The borehole apertures of *I. cheongpodensis* (zooidal length150 µm and width 50 µm) are described as teardrop shaped (typical spindle shape as referred to here), along with *I. orbignyana* (zooidal length 90 µm and width 52 µm) these are the smallest reported sizes for immergentiids and therefore easily distinguishable from *I. stephanieae* sp. nov. Intercalary kenozooids can insert between cystid appendages (cystid appendages are equivalent to stolo-like threads by Silén, 1947), connecting them to each other (Fig. 5h, i). The lophophoral base is broader in *I. stephanieae* sp. nov. compared to *I. patagoniana* and *I.* cf. *suecica* (see below).

See Tables S4 and S5 for a summary of zooidal metrics and characteristics of immergentiids used in this study as well as a summary of collection data from literature, including type/paratype specimen numbers, respectively.

**Habitat**: Bryozoan occurs in living and dead shells of gastropods, especially *Littorina littorea* Linnaeus, 1758 and *N. lapillus* Linnaeus, 1758. Gastropods occur in the intertidal zone around Roscoff and commonly found in tide pools during low tide, especially under rocks and boulders. Camouflaged *L. littorea* was commonly found on the blades of algae, *Fucus vesiculosus* and *F. serratus* Linnaeus, 1753.

**Occurrence**: Additional material (other than holotype and paratype) of the immergentiid found in gastropod shells (see above) from the intertidal zone in Roscoff, specifically in the following locations: Santec, Île Callot and around the Roscoff Marine Research Station. Location in relation to the research station: North - area directly behind the station; East - extending toward the area under and beyond the Roscoff bridge (Pont de Roscoff); West - in the channel midway between the research station and Le rocher au visage.

***Immergentia suecica*** Silén, 1947

Figure S1d–f

*Immergentia suecica* Silén, 1947, p. 41, figs. 58, 60, 61

**Material examined**. NORTH SEA • extracted zooids and colony borings in bivalve shell; Skagerrak, Sweden, Gullmar Fjord, North of Flatholmen; depth: 45 m; 16 August 1946; L. Silén leg.; *I. suecica* in shell of *Pseudamussium peslutrae* Linnaeus, 1771; holotype: SMNH-Type-2366.

NORTH-EAST ATLANTIC – **France** • extracted zooids and colony borings in several bivalve shells; Stolvezen; 48º42.847’N 03º53.500’W, 48°40.000' N 3°52.999' W; depth: 15–25 m; 3 September 2021; ‘Neomysis’ cruise; characterized by bivalves, gastropods, algae (brown, red and green) and brown sediment, immergentiid colonies in bivalve shells: *Lutraria lutraria* Linnaeus, 1758, *Ensis* sp. Schumacher, 1817, *Pecten maximus* Linnaeus, 1758, and *Anomia* sp. Linnaeus, 1758; FR21–GP21, FR21–A50, FR21–PF54, FR21–FP28, FR21–GP28, FR21–A25, FR21–GP25, FR21–A22, FR21–GP12, FR22–72A, FR22–74A, FR23-A3, FR21-28; GenBank: SAMN38786229 • extracted zooids and colony borings in bivalve shells; Stolvezen; 48º42.847’N 03º53.500’W, 48°42.846' N 3°53.5' W; depth: 15–25; 5 October 2021; ‘Neomysis’ cruise; FR21–D6/7 • extracted zooids and colony borings in bivalve shells; Térenéz; 48º41.532’N 03º52.075’W; depth: 10 m; 10 September 2021, 14 September 2021, 5 October 2021; ‘Neomysis’ cruise; characterized by rocks, living bivalves, red algae and dark sediment; FR21–A55, dried shells; GenBank: SAMN38786228 • extracted zooids and colony borings in bivalve shells; Chateaux du Taureau, Roscoff; 48°40.2' N 3°53.12' W, 48°40.200' N 3°52.999' W; depth: 10–15 m; September 2020, 4, 5, 8, 12 September 2021, December 2022; ‘Neomysis’ cruise; FR19-6F, FR21-A6, FR22–33A, dried shells – **Norway** • one heavily bored shell; Trondheim Fjord, Norway; 63º51.479’N 11º04.354’E; depth: 12 m; 08 July 2014; P. Kukliński leg.; N19 gastropod 3.

**Description - *I. suecica* (Holotype):** Borehole apertures oval (Fig. S1d) with average width of 55 ± 9 µm. Autozooids typical vase shape, length 250 ± 11 µm and width of 68 ± 5 µm, with rounded or sometimes tapered basal tip, curved toward primary cystid appendage (Fig. S1e). Polypide with 9 tentacles (Fig. S1f) and lophophoral anus. Intercalary kenozooids may connect cystid appendages. Tubulets present. Presumed developing zooid length 131 µm and width 62 µm.

**Fig. 10:**
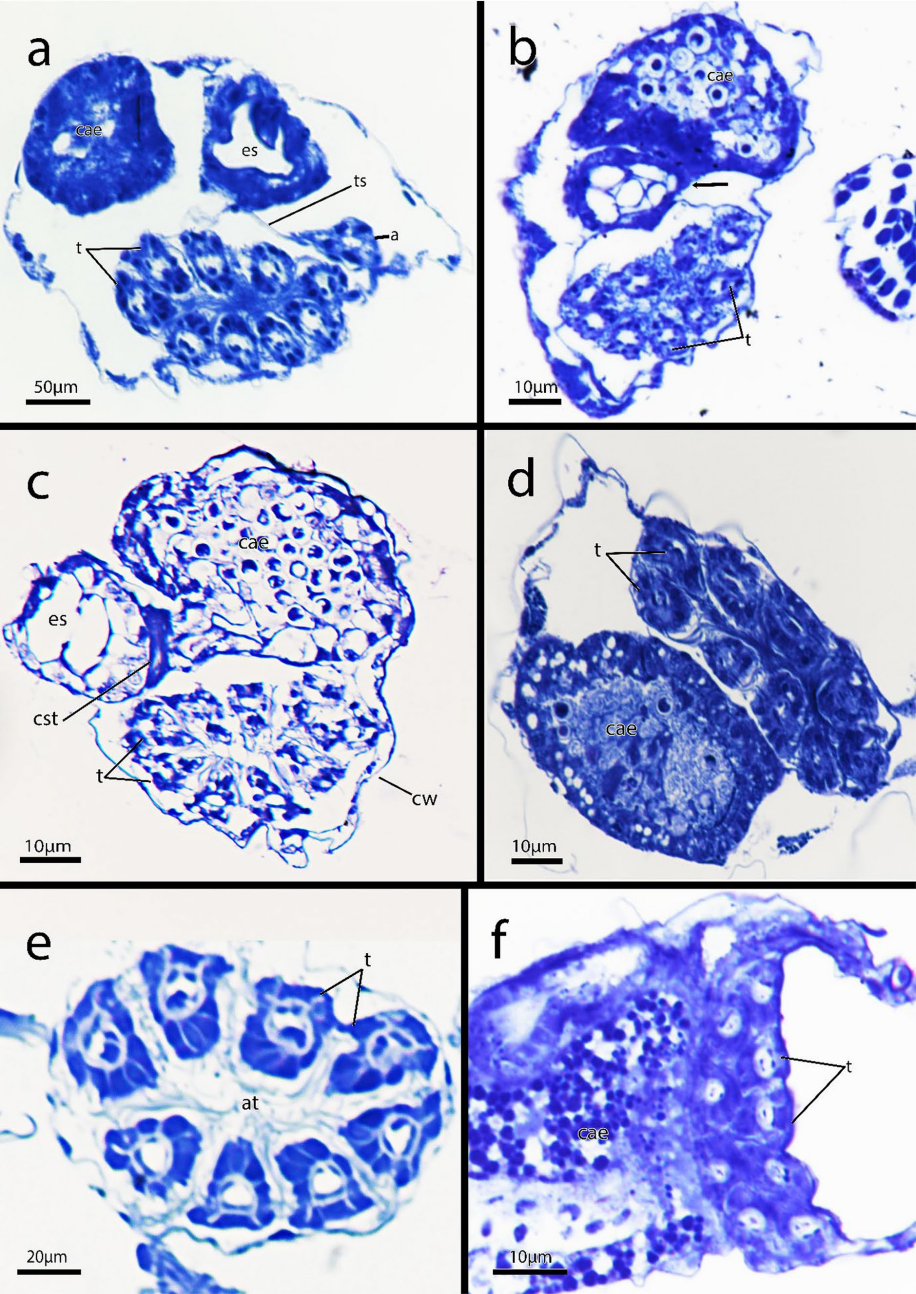
Semi-thin histological serial sections of retracted polypides of different *Immergentia* species. **a** Cross section *Immergentia patagoniana* with 9 tentacles collected from Burdwood Bank in the south-western Atlantic Ocean. Termination of anus in tentacle sheath. **b**. Cross section *Immergentia patagoniana*, transition from fore- to mid-gut (arrow)** c** Cross section of *Immergentia* cf. *suecica* from France with 9 tentacles. Cardiac constrictor at transition from fore- to mid-gut. **d** Cross section of *Immergentia* cf. *suecica* with 9 tentacles collected from Trondheim Fjord, Norway.** e** *Immergentia pohowskii* sp. nov. from Taiaroa Head, New Zealand (NIWA-161223) with 8 tentacles.** f** *Immergentia* cf. *zelandica* from the Inner Otago shelf, New Zealand, with 9 tentacles. Abbreviations: a – anus, at – atrium, cae – caecum, cst- cardiac constrictor, cw – cystid wall, es – oesophagus, t – tentacles, ts – tentacle sheath

**Description – *Immergentia* cf. *suecica* from France** (Figs. 3e, 3c, 7c, 8d, 9e and 10c): Borehole apertures oval or spindle shaped and enantiomorphic (Fig. 9e) with average width of 50 ± 9 µm. Colony not dense (Fig. 9e). Zooids irregularly and broadly spaced (Figs. 3e and 9e) at intervals measuring 917 ± 175 µm. Autozooids typical vase shape, length 262 ± 48 µm and width of 74 ± 10 µm, with tapered or sometimes rounded basal tip (Fig. 3c), curved toward primary cystid appendage (Fig. 8d). Polypide with 9 tentacles (Fig. 10c) and low-positioned lophophoral anus (Fig. 7c). Opposite branching from the primary cystid appendage common (Fig. 9e). Up to three secondary cystid appendages form in middle sections of the zooids. Tubulets may be present or absent. Intercalary kenozooids may connect primary or secondary cystid appendages. Sac zooids typically bulb shaped and overall 1/3 size of autozooid.

**Description – *Immergentia* cf. *suecica* from Norway** (Figs. 7d, 7e, 9b and 10d): Borehole apertures oval shaped and enantiomorphic (Fig. 9b) with average width of 45 ± 9 µm. Zooids regularly and broadly spaced (Fig. 9b) at intervals measuring 952 ± 365 µm. Autozooids typical vase shape, length 303 ± 25 µm and width 100 ± 32 µm, with tapered or sometimes rounded basal tip, curved toward primary cystid appendage. Polypide with 9 tentacles (Fig. 10d) and low-positioned lophophoral anus (Fig. 7d, e). Up to three secondary cystid appendages form in middle sections of the zooids with thinner processes extending from primary appendages. Tubulets may be present or absent. Intercalary kenozooids may connect primary or secondary cystid appendages. Sac zooids typically bulb shaped and overall 1/3 size of autozooid.

**Remarks:** Direct measurements of *I. suecica* was done on 4 zooids, one of these zooids was small compared to the others and is presumably a developing zooid. These were embedded and sectioned for histological analysis. The presence of intercalary zooids is confirmed.

Silén (1947) described the colony morphology of *I. suecica* as an intermediate between that of *I. californica* and *I. zelandica*. Which means that *I. suecica* may have sections where zooids along the primary cystid appendage may occur at regular intervals as seen in *I*. cf. *suecica* from Norway (Fig. 9b) and lateral intercalary kenozooids/cystid appendages arise at irregular intervals in *I*. cf. *suecica* from France (Fig. 9e). Unfortunately, the colony morphology of *I. suecica* could not be determined because the substrate was fragile and eroded. The cystid appendages were also not visible from the shell substrate. DNA sequences were not recovered because material was fixed in Bouin’s and/or Flemmings’ solutions, that disrupt DNA. Therefore, we need to rely on the original descriptions and illustrations. Here, opposite and irregular branching patterns of lateral cystid appendages and/or intercalary kenozooids occur, differing from the type *I. californica*, where branching alternates in either direction (see Silén, 1947, p. 41, fig. 58).

In specimens from both France and Norway the primary cystid appendage may or may not bare tubulets. Similarly, intervals between zooids are up to two or three times greater than those in *I. stephanieae* sp. nov. Scaled drawings of *I. suecica* from Sweden (the type locality) by Silén (1947) permitted comparison of interzooidal intervals and zooid size with specimen examined here. Interzooidal intervals based on scaled drawings of *I. suecica* (five zooids) by Silén (1947, fig. 60) and reproduced by Prenant and Bobin (1956, p. 230, fig. 100, colony III) measured at 931 ± 177 µm, *I.* cf. *suecica* from France, 917 ± 175 µm and *I.* cf. *suecica* from Norway, 952 ± 365 µm.

Zooid size of *I.* cf. *suecica* from France resembles the sizes from the *I. suecica* measured here. However, this value is slightly smaller than those from illustrations of Silén (1947) and Hayward (1985). The autozooid size for *I. suecica* varies greatly between authors. Considering measurements from illustrations in literature, *I.* cf. *suecica* from France had a size range close to that of *I. suecica* by Hayward (1985, p. 105 drawing A) length 200 ± 18 µm and width 97 ± 13 µm but these measurements were based on two zooids. On the other hand, *I.* cf. *suecica* from Norway had a length range of 303 ± 25 µm comparable to the type *I. suecica* 310 – 340 µm illustrated by Silén (1947; see Table S5). According to illustrations from Prenant and Bobin (1956, p. 230) reproduced from Silén (1947, figs. 60 and 61) the measurements were as follows: five autozooids (image III) length 305 ± 43 µm and width 89 ± 13 µm; (image IV) length 444 ± 24 µm and width 141 ± 9. No zooids exceeding 400 µm has been observed here. It is important to consider that size differences can be attributed to improved precision with modern tools or the reproduction of images and scales from Silén’s original work as opposed to measurements of fresh material. In fact, the scale bar for the colony (image IV) varied from our measurements by about 50 µm.

The anus position of *I.* cf. *suecica* differs from that of *I. suecica*, which was reported to have a vestibular anus (see Schwaha, 2020c). It is important to note that the latter was based on the illustrations and descriptions from Silén (1947), which may not be completely accurate, as pointed out by Schwaha (2020c), and a lophophoral anus is confirmed for *I. suecica* in the analysis of the type material.

Reverter et al. (1995) collected immergentiids from Château du Taureau in Roscoff but were not able to assign a species because specimens were dry. In this study, material was collected from the same location and other subtidal locations around Roscoff, confirming the presence of immergentiids. Differences in zooidal size between the specimen from France and Norway can also be attributed to a high sampling of samples from France (the main study site) and only one shell from Norway, which probably influenced variability. Apart from the obvious size differences, other characteristics of specimen from both locations were quite similar.

Since the attempt to redefine all characteristics from the type material and genetic verification of the species were unsuccessful, the placement of *I.* cf. *suecica* from France and Norway remain inconclusive and are therefore tentatively placed as *I. suecica.* Considering the condition of the type material, a neotype needs to be assigned. In addition, more material from Norway would also need to be obtained and analysed.

***Immergentia patagoniana*** Pohowsky, 1978

Figures 8a–c, 9a and 10a, b

*Immergentia patagoniana* Pohowsky, 1978, p. 126, pl. 24, fig. 5

*Immergentia zelandica patagonica* López-Gappa, 1981, p. 24, pl.1, figs. 1–3; new synonym

**Material examined.** SOUTH-WEST ATLANTIC • dense colonies, heavily bored in gastropods, *Savatiera chordata* Castellanos, Rolán & Bartolotta, 1987; Burdwood Bank, near Argentina; 54º25.144’S 59º12.892’W; depth: 120; 30 March 2016; ‘R/V Puerto Deseado’ cruise; ARG–E28–L51 • dense colonies, heavily bored in gastropods, *Argeneuthria cerealis* de Rochebrune & Mabille, 1885; Burdwood Bank; 54º00.240’S 61º04.762’W; depth: 139; 08 May 2017; ‘R/V Puerto Deseado’ cruise; ARG–E28–L290 • dense colonies, heavily bored in gastropods, *Trophon ohlini* Strebel, 1904; Burdwood Bank; 54º30.390’S 59º48.654’W; depth: 105; 10 April 2016; ‘R/V Puerto Deseado’ cruise; ARG–E28–L198 • dense colonies, heavily bored in gastropods, *Pareuthria atrata* E. A. Smith, 1881; Burdwood Bank; 54º31.679’S 61º27.979’W; depth: 137; 31 March 2016; ‘R/V Puerto Deseado’ cruise; ARG–E28–L88 (gastropods 1 & 2).

**Description**: Colony dense with zooids irregularly spaced (Fig. 9a), average interval between neighbouring zooids 268 ± 63 µm. Borehole apertures elongated, resembling a bulged ‘S’ shape and strongly enantiomorphic (Fig. 9a) with average width of 36 ± 8 µm. Typical vase shaped autozooids, length 333 ± 26 µm and width of 124 ± 32 µm, with tapered basal tip (Figs. 7a and 8a–c), acutely positioned in the substrate. Some zooids may curve at acute angle toward primary cystid appendage (Fig. 8a). Short narrow projection present in basal end of some zooids. Polypide with 9 tentacles (Fig. 10a, b) and low-positioned lophophoral anus (Fig. 7b). Opposite branching from the primary cystid appendage can occur (Fig. 9a). Tubulets on the primary cystid appendage may be present or not. Up to three secondary cystid appendages in mid-region of the zooids. Intercalary kenozooids may connect primary or secondary cystid appendages. Sac zooids bulb shaped with distal flat end and 1/3 to 2/3 of autozooid size (Fig. 8g). Basal end of sac zooids typically rounded.

**Remarks**: Pohowsky (1978) described the species based on d’Orbigny’s material collected from Patagonia, Argentina, between 1826 and 1833. Though only micrographs of borehole apertures of the species were presented by Pohowsky (1978), he described deep depressions of the apertures, the strong enantiomorphic borehole aperture, and the strong curvature of some zooids. Consequently, we consider that the present material corresponds to the same species. Therefore, the soft body morphology for *I. patagoniana* is presented here for the first time. The acute curvature of zooids has also been observed in *I. angulata* (Soule & Soule, 1969), which differs in size and tentacle numbers. López-Gappa (1981) described *I. zelandica patagonica* from the same area including additional characteristics such as zooid size (from resin casts), colony structure, tentacle number, position, and size of cystid appendages. All characters mentioned above fit those of samples collected and investigated in this study from the same region in the southwest Atlantic. The curvature and two pairs of secondary cystid appendages are visible in micrographs of casts by López-Gappa (1981, figs. 1 and 2). Furthermore, he also indicates a proximal projection in the tip of a zooid (López-Gappa, 1981; fig. 3). Hence, we suggest that *I. zelandica patagonica* be considered a junior synonym of *I. patagoniana* on the bases that the distribution and collection sites, morphology, and gastropod hosts are similar to those described here and by Pohowsky (1978).

***Immergentia pohowskii*** Johnson & Schwaha sp. nov.

**Fig. 11:**
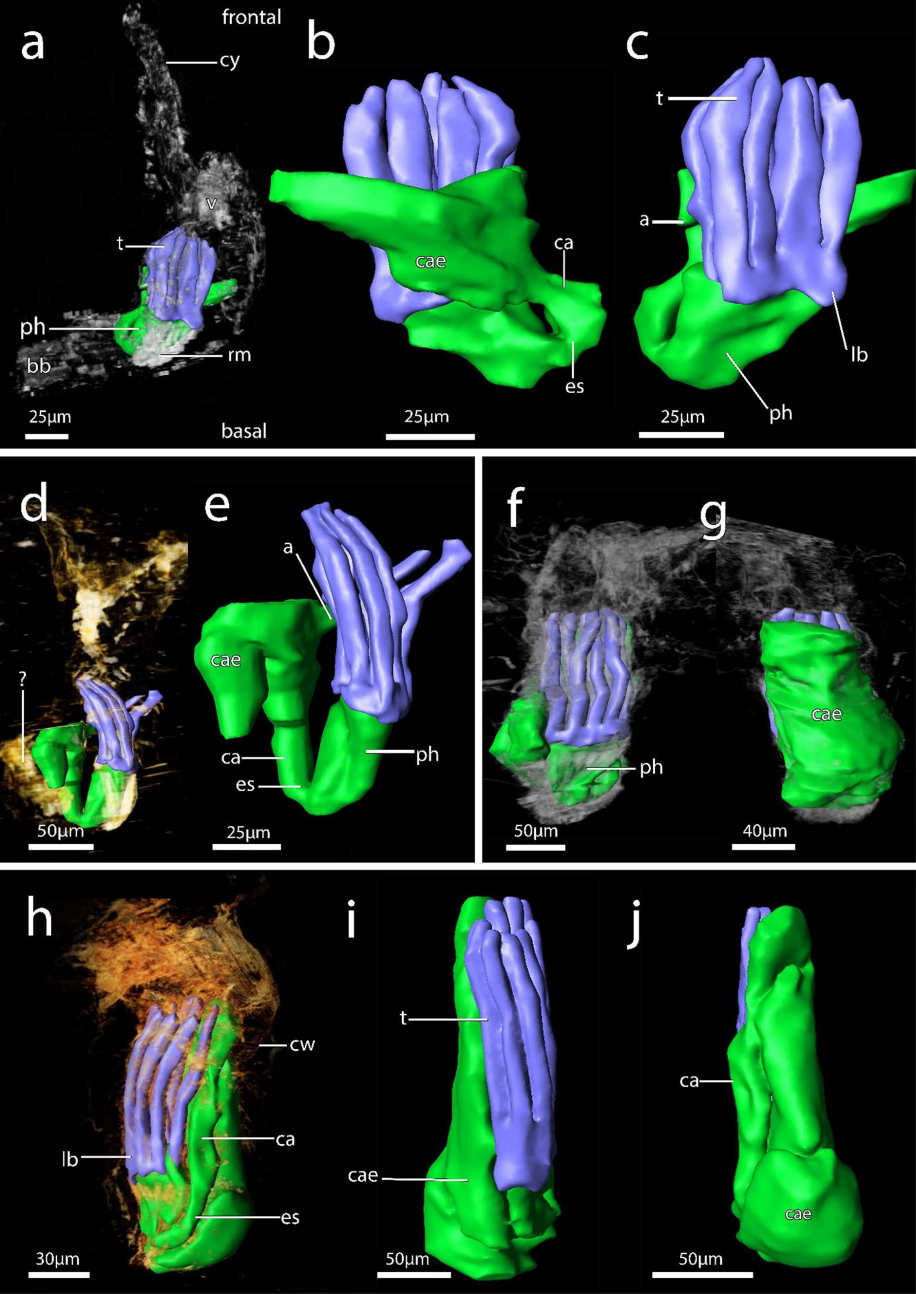
3D reconstruction of immergentiids from New Zealand. **a**–**c** Overview of autozooids of *Immergentia pohowskii* sp. nov. with 8 tentacles (Locality: Taiaroa Head). **a** Oral view of autozooid. **b** Same autozooid. View of digestive tract. Transition from pharynx to oesophagus pinched. **c** Same zooid. The low-positioned lophophoral anus. **d** Autozooid of different individual of *Immergentia pohowskii* sp. nov. Possible brown bodies indicated by? **e** Mid-positioned lophophoral anus. **f** Oral view of autozooid of *Immergentia* cf. *zelandica* (Locality: Inner Otago shelf) with 9 tentacles. **g** Same zooid. Anal view of large caecum. **h** Overview of autozooid of *Immergentia* cf. *zelandica*. **i** Same autozooid. Oral view of polypide. **j** Same autozooid. Anal view of digestive tract. Abbreviations: a – anus, bb – brown body, cae – caecum, cw – cystid wall, cy – cystid appendage, es – oesophagus, lb- lophophore base, ph—pharynx, rm – retractor muscle, t – tentacles, v – vestibulum

Figures 3b, 9d, 10e and 11a–e

**Material examined**. **Holotype**: SOUTH-WEST PACIFIC OCEAN • small colony in molluscan shell fragment; New Zealand, South Island, Taiaroa Head; 45º45.36’S 170º47.36’E; depth: 23 m; 05 November 2021; ‘PB Otago Shelf’ cruise; Taiaroa Head sediment characterized by coarse shells; ZooBank: urn:lsid:zoobank.org:act:B50F54B2-0A06-4AE8-96C3-D38B2AB4F3E7; GenBank: SAMN38786231; NIWA-161223.

**Paratype**: SOUTH-WEST PACIFIC OCEAN • slides, serial sections; same collection data as for holotype; NIWA-161224.

**Etymology**: The species is named in honour of Robert A. Pohowsky for his invaluable contributions to the nomenclature and characterization of boring bryozoans.

**Diagnosis**: Endolithic bryozoan with regularly spaced borehole apertures and 8 tentacles.

**Description:** Borehole apertures oval or spindle shaped, enantiomorphic (Fig. 9d) with average width of 35 ± 9 µm. Average distance between adjacent zooids is 943 ± 118 µm. Autozooid shape cylindrical, length 299 ± SD 30 µm and width of 76 ± 8 µm, slightly narrowed at mid-zooidal region and elongated rounded basal tip (Fig. 3b). Some zooids have tapered basal tip slightly curved toward primary cystid appendage (Figs. 3b and 11a). Polypide with 8 tentacles (Fig. 10e) and low to mid-positioned lophophoral anus (Figs. 11c, e). Opposite branching from the primary cystid appendage observed (Fig. 9d). Tubulets on the primary cystid appendage may be present or not. Up to three secondary cystid appendages form in the middle to frontal sections of zooids. A proximal cystid appendage may also occur. Intercalary kenozooids may connect primary or secondary cystid appendages. Sac zooids typically vase or bulb shaped and overall 1/3 or 2/3 the size of autozooid.

**Remarks**: Two conspicuous differences in the species from New Zealand are that *I. pohowskii* sp. nov. has 8 tentacles and oval to spindle-shaped apertures while *I.* cf. *zelandica* has 9 tentacles and circular to oval shaped apertures. In the shell fragments the borehole apertures of *I. pohowskii* sp. nov. were regularly spaced but dense in others where the interzooidal intervals could not be determined. The zooid size range of *I. pohowskii* sp. nov. sometimes exceeded 100 µm. A proximal cystid appendage may also occur as that observed in *I. stephanieae* sp. nov., albeit rare. Lophophoral anus terminates distal of the lophophoral base, in the mid region of the lophophore, different from that of *I. patagoniana*, *I. stephanieae* sp. nov*.* and *I.* cf. *suecica*. However, in sectioned individuals, the lophophore and digestive system are relatively smaller, not occupying the larger part of the body cavity. Prior to this study, *I. angulata* from the Hawaiian Islands was the only other species known to have 8 tentacles (see Soule & Soule, 1969) but differs from *I. pohowskii* sp. nov. in size, and their zooids are acutely bent in the substrate, similar to those of *I. patagoniana*. In addition, genetic distances based on *cox1* support the notion that *I. pohowskii* sp. nov. and *I.* cf. *zelandica* are distinct (see Phylogenetic analysis).

***Immergentia zelandica*** Silén, 1946

Figure S1a–c

*Immergentia zelandica* Silén, 1946, p. 3, figs. 11, 12

**Material examined**. SOUTH-WEST PACIFIC OCEAN• heavily bored shell of *Buccinulum littorinoides* Reeve, 1846 with immergentiid colony and decalcified zooids in the extra cellular matrix of gastropod; New Zealand; North Island, Slipper Island, depth: intertidal; 20 December 1914; Mortensen’s Pacific exped.; holotype, SMNH-Type-3065.

SOUTH-WEST PACIFIC OCEAN• dense colony in shell belonging to Buccinoidea Rafinesque, 1815; New Zealand; South Island, Otago inner shelf, 45º45.87’S 170º49.50’E; depth: 40 m; 05 November 2021; ‘PB Otago Shelf’ cruise; inner Otago shelf characterized by sticks, mud, molluscs, crabs; NZ21PB10, gastropods 1, 2 & 3; GenBank: SAMN38786232.

**Description – *Immergentia zelandica* (Holotype):** Borehole apertures circular with an average width of 87 ± 14 µm (Fig. S1g). Most zooids with typical vase shape with a length of 249 ± 29 µm and width 59 ± 12 µm, basal end mostly rounded, few tapered, and may curve strongly in the mid-zooidal area toward primary cystid appendage or not at all (Fig. S1h). Few zooids with short projections at the basal end (Fig. S1h). Autozooids with 9 tentacles (Fig. S1i).

**Description – *Immergentia* cf. *zelandica*** (Figs. 3a, 9c, 8e, h and 10f): Borehole apertures circular or oval shape (Fig. 9c) with average width of 52 ± 6 µm. Zooids in the colony densely packed and irregularly placed. Basal end of zooids rounded, few tapered, and slightly curved toward primary cystid appendage or not at all (Figs. 3a and 8e). Few zooids slightly narrowed at mid-zooidal region and elongated rounded basal end (Fig. 3a). Average length of zooids is 284 ± 38 µm and an average width of 85 ± 11 µm. Autozooids with 9 tentacles (Fig. 10f). Sac zooids vase shaped and overall 2/3 size of autozooid (Fig. 8h). Up to four secondary cystid appendages form in the middle to distal sections of zooids with thinner processes extending from primary and secondary appendages (Figs. 3a and 8f). Reproductive zooid with a bulge to one side of the zooid (Fig. 8f).

**Remarks**: Zooids of *I. zelandica* were in a degraded state, and few structures of soft body morphology, such as tentacles, could be distinguished. The borehole apertures (87 ± 14 µm) are comparatively larger than the width of the zooids (59 ± 12 µm) but almost the size of the zooidal width (80 µm) initially reported by Silén (1947). Colony is densely packed and difficult to distinguish cystid appendages and measure interzooidal intervals. The primary cystid appendage may or may not bear tubulets. More than two secondary cystid appendages commonly observed than that of any other species in this study. Thinner processes occur proximally beyond mid-zooidal region, not observed in any other species. Zooids pinched in mid-zooidal region are less common than in *I. pohowskii* sp. nov. Fewer individuals with tapered basal tip compared to *I.* *pohowskii* sp. nov. Position of anus difficult to ascertain (Fig. 11f–j).

The zooid size range of both *I. pohowskii* sp. nov. and *I.* cf. *zelandica* from southern New Zealand sometimes exceeded 100 µm. Here, colony morphology of *I.* cf. *zelandica* closely resembles the description of the type *I. zelandica* characterized as a dense, irregularly placed colony, zooids with 9 tentacles and numerous processes extending from the zooids making it difficult to distinguish primary and secondary cystid appendages (see Silén, 1947). Both *I. zelandica* and *I.* cf. *zelandica* here have a similar size range approx. 249 and 284 µm respectively, while *I. zelandica* var *minuta* was slightly smaller 207–218 µm (Soule, 1950). On the contrary, type of *I. zelandica*, described from the intertidal zone in Slipper Island (north of New Zealand) had a zooid length 310 and width 80 µm. *Immergentia* cf. *zelandica* was collected from the subtidal zone, further south near Otago inner shelf. Pohowsky (1978) reported a minimum length of 210–250 µm for the species (measured from Soule, 1950), taking the length and width of *I. zelandica* var *minuta* into consideration, 207 – 218 and 48 µm respectively (see Soule, 1950). DNA sequences were not recovered from *I. zelandica* and zooids for histology were in a mushy state; therefore, the soft-body components could not be differentiated. Another possible explanation for the size variation could be that the zooids (stored in a decalcified state) shrank but this is purely speculative.

The drawings of *I. zelandica* do not depict the shape of zooid aperture (see Silén, 1947 fig. 62, p. 43), nor is it explicitly mentioned (only the general zooidal aperture shape typical of immergentiids is described). In this study the borehole aperture is oval to spindle shaped (Fig. 9c) similar to illustrations of *I. zelandica* var *minuta* borehole apertures (Soule, 1950 pl. 2 fig. 4, p. 365) from the Philippine Islands. In contrast, that of the examined type material is circular to oval. In *Immergentia pohowskii* sp. nov. and *I. zelandica*, the autozooids may be pinched with an elongated projection extending proximally from the mid-zooidal region. Though there are similarities between the type *I. zelandica* and *I.* cf. *zelandica*, the difference in aperture size, uncertain soft-body morphology of the type material, and difference in location and depth of collection (intertidal vs subtidal) casts doubts on whether these two species are the same.

## General morphological characters of Immergentiidae

### Apertural area

Colonies of *Immergentia* are typically enantiomorphic which means the zooidal apertures are arranged to deflect either to the right or left of the primary cystid appendage (pcy). This deflection is often visible in the borehole apertures (Figs. 1c and 9a, d).

The aperture of zooids is quadrangular, and the apertural area may have three distinguishable shapes, seen in the shell or substrate tracings (Figs. 1a–c and 9). First, borehole apertures are typically spindle shaped ranging from a simple spindle (Fig. 1c) to strongly resembling a bulged ‘S’ (Fig. 9a). Here, cystid appendages extend laterally, from both sides of the zooids, at the pointed edges of this spindle slightly below the shell surface (Fig. 9e). A vane in the angle between the primary cystid appendage and zooid’s distal cystid wall may also be present. Second, borehole apertures have an oval (Fig. 9b and c) or lastly, circular shape (Fig. 9c). In these instances, where the ends of the zooidal apertures are not attenuated, tubular primary cystid appendages extend well below the zooidal apertural area. The musculature of the zooidal apertural region is described in detail below. The vestibular wall is funnel-shaped, with the broader end located distally toward the zooid aperture and gradually narrowing basally (Fig. 2).

**Fig. 12:**
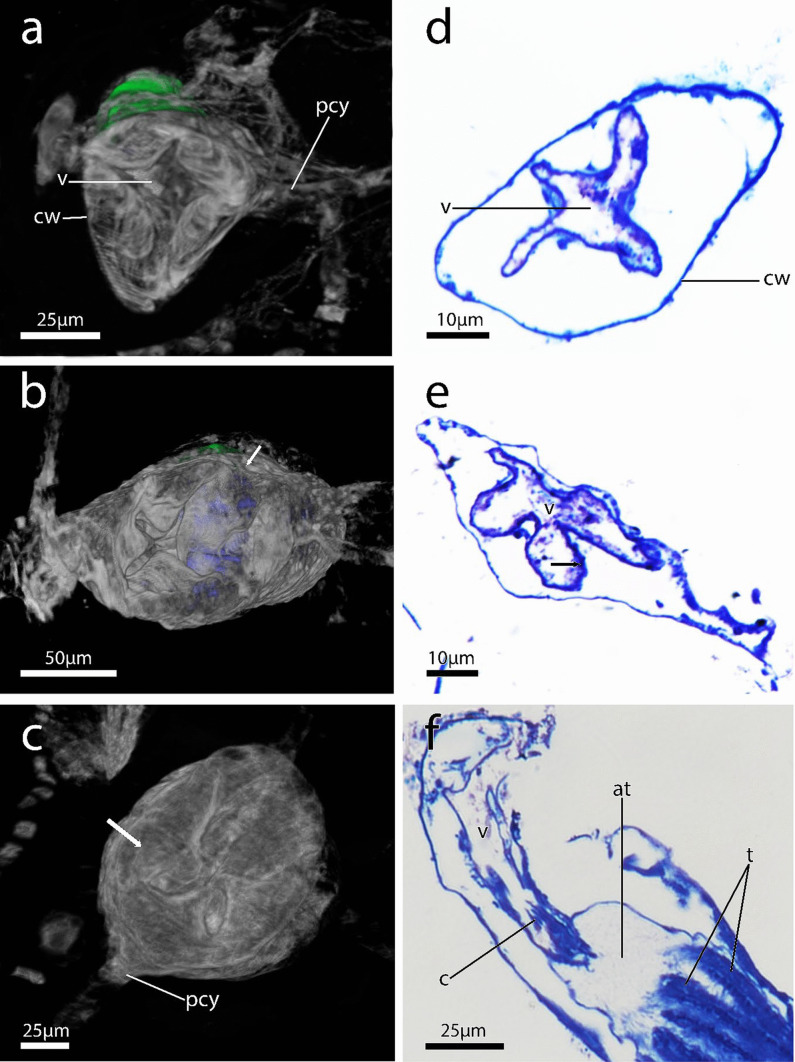
3D reconstructions with volume rendering and semi-thin histological sections of the zooidal apertural area of immergentiids. **a** Frontal view of quadrangular aperture and oval shaped apertural area of *Immergentia* cf. *suecica* from Roscoff, France. Cystid terminates vestibulum area. **b** Spindle shaped apertural area of *Immergentia stephanieae* sp. nov. from Roscoff, France. Zooidal aperture (arrow).** c** Quadrangular oval shaped aperture *Immergentia stephanieae* sp. nov. Zooidal aperture (arrow). **d** Vestibulum and thickened vestibular wall of *Immergentia* cf. *suecica* from France. **e** Apertural area of *Immergentia patagoniana* from Burdwood Bank, south-western Atlantic Ocean.Vestibulum and thickened vestibular wall (arrow).** f** Longitutinal section of *Immergentia* cf. *suecica* from France. Collar at proximal end of vestibulum. Abbreviations: at – atrium, c – collar, cw – cystid wall, pcy – primary cystid appendage, t – tentacle, v – vestibulum

In feeding autozooids, the vestibular wall typically appears shorter than in reproductive zooids, proportionally measuring about 18% ± SD 0.0031 and 29,3% ± SD 0.087 to the length of the zooid, respectively (Fig. 2a). A diaphragmatic sphincter in the proximal part of vestibular wall separates the vestibulum from the atrium (Musculature system, below). A collar consisting of cuticular folds borders the proximal vestibular wall at the diaphragm (Fig. 12f).

### Cuticle and interzooidal pores

The cuticle is unmineralized and forms the protective outer cystid wall of a zooid. In the frontal zooidal apertural region, the cuticle appears thicker than the rest of the zooid, specifically the folds extending from the quadrangular aperture (frontal view) (Fig. 12a–c). Below the substrate surface, the pcy extends laterally from two of the corners or the attenuated ends of the aperture and connects to the pcy of a neighbouring zooid (Fig. 4). A septum separates connecting zooids (Figs. 2a, d and 5). Representatives of all investigated species may possess none or up to three (*I. patagoniana*, *I*. cf. *suecica*, and *I. pohowskii* sp. nov.) or four (*I. stephanieae* sp. nov. and *I.* cf. *zelandica*) scy (Figs. 3 and 4). Tubulets arising from the pcy are common in individuals of all examined species. These tubulets are thick tubular processes that extend frontally from the pcy toward an opening in the substrate (Figs. 2a and 5a), never from any other part of the zooid. Similarly, additional thinner processes (≤ 5 µm) may also arise from the cystid appendage (Fig. 4f) but this condition is seldom observed. An appendage derived from the cystid wall may occur at the basal end of the cystid wall (Fig. 8i), as observed in *I. stephanieae* sp. nov. and *I.* cf. *zelandica*. Intercalary kenozooids can attach to either a primary or secondary appendage (Figs. 4a and e) and may bear tubulets (when present in the former) or thinner processes (in both positions).

In zooids where the aperture is oval or spindle-shaped the interzooidal septum is located further away from the frontal apertural ends (laterally), where the cystid wall tapers off into a thinner tubule i.e. the primary cystid appendage (Fig. 12a–e). In individuals with a rounded apertural area and/or significantly reduced vane, the primary cystid appendage is cylindrical from the onset, and the septum can occur closer to the zooidal cystid wall (Fig. 12c). In such individuals, the primary cystid appendage appears deeper below the surface of the substrate.

A septum forms at the junction of the cuticles of neighbouring zooids. The septum is embedded with a cuticular pore plate displaying the uniporate condition (Fig. 5b–j). The special cell plugs the pore and is dumb-bell shaped, with its nucleus only displayed on one end (of the dumb-bell) (Fig. 5g and j). Additional putative ‘limiting’ cells encircle the special cell (Fig. 5c and g).

### Zooidal polymorphs

#### Kenozooids (heterozooids)

Kenozooids are specialized non-feeding zooids with modified cystids, devoid of a polypide or any remnants thereof. Two types of kenozooids are present in *Immergentia*, intercalary kenozooid and sac zooids. Intercalary kenozooids are tubular structures derived from the cystid wall and devoid of any specific cells except those associated with the cystid wall and pore plate (Figs. 4a, e and 5h, i). Special cells interconnect zooids or zooids to other intercalary kenozooids and eventually to a zooid (Fig. 5c–g and j).

Sac zooids are characterised by their large basal extension and can have a variable shape in *Immergentia* ranging from bulb to vase shapes (Figs. 3a, b and 8g, h). Their size typically does not exceed that of an autozooid. At the frontal end, these zooids lack a distinct aperture or orifice. They have cystid appendages that extend from the cystid wall and connect to an intercalary kenozooids or cystid appendage of a neighbouring zooid along the pcy (Fig. 8g and h). Sac zooids are filled with birefringent spherules or granules. The basal tip of sac zooids may or may not be tilted in the direction of the pcy (Fig. 3a, b).

#### Autozooidal characteristics

Autozooids are zooids capable of feeding and possess a functional polypide. Generally, zooids are vase-shaped but shapes can vary from bulbous to cylindrical (Figs. 2, 3 and 8a–e). Zooids are not necessarily vertical in the substrate but rather slightly tilted, with the basal end curved in the direction of the primary cystid appendage (Figs. 1f, 2a and 9e). The basal end may be tapered or rounded (Figs. 2a–c, 3 and 8a–e). In some instances, zooids display extreme curvature in the mid-zooidal region, commonly at the distal region of the lophophore and the proximal vestibular wall area (Fig. 8a). Severe bulges from the mid-zooidal to basal tip usually a result of the size and position of the caecum (Fig. 8b, c and e) or a brooded embryo (Figs. 4a and 8f), or accumulation of brown bodies, can distort the typical vase or cylindrical shape of a zooid. In *Immergentia* cf. *zelandica* contorted zooids were frequently encountered (Fig. 3a).

Larvae are brooded in the tentacle sheath of an autozooid where the polypide has degenerated (Figs. 2a, c–d, 4a–b, and 8f). No specialized zooids (gonozooids) for reproduction were observed in the examined material.

### Lophophore and digestive tract

**Fig. 13:**
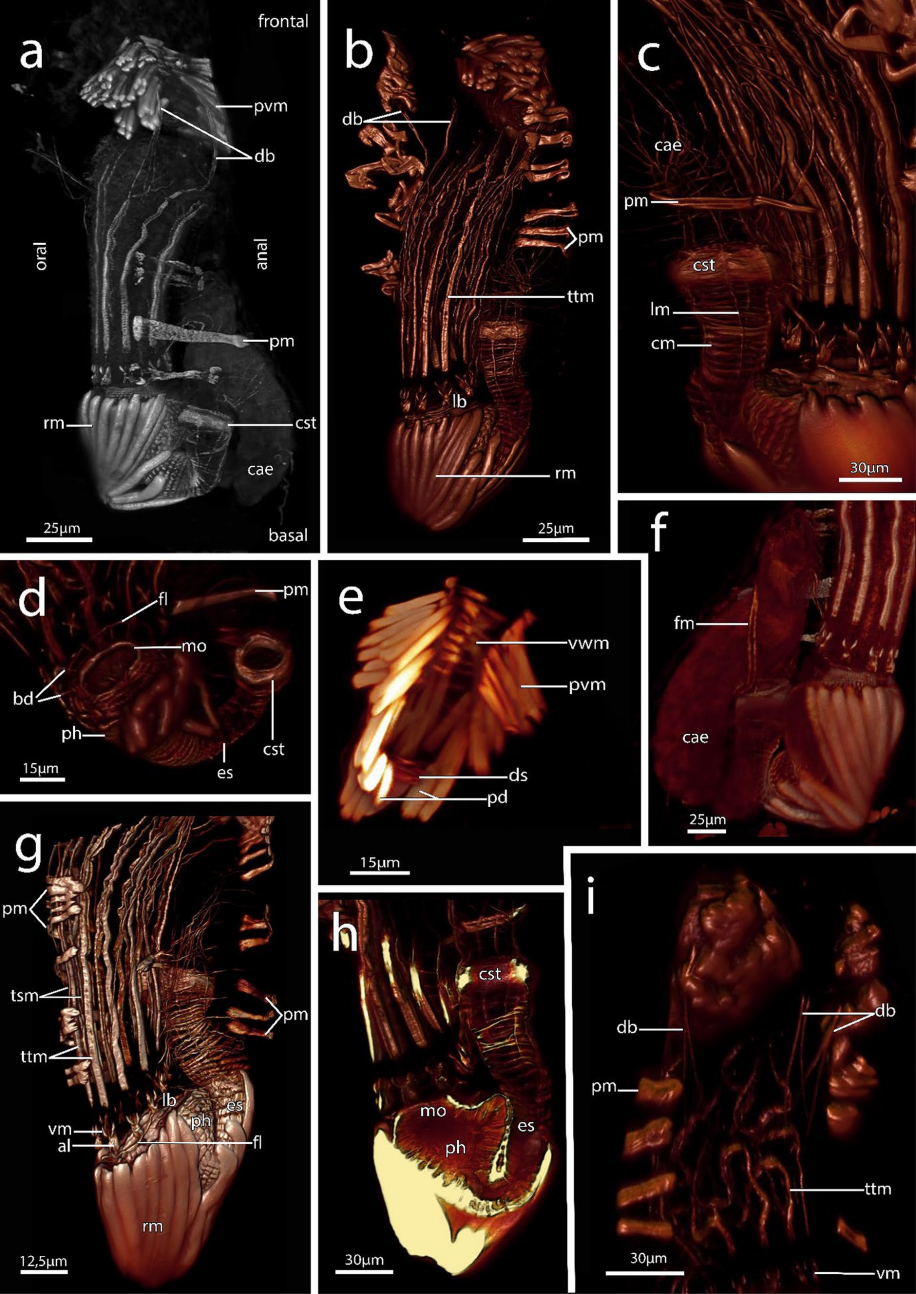
Muscular system of *Immergentia* stained for f-actin, CLSM datasets. **a** Overview of *Immergentia stephanieae* sp. nov. autozooid from Roscoff, France. General arrangement of autozooid. Muscles in apertural region are parieto-vestibular muscle and duplicature bands. Smooth retractor muscles from the proximal cystid wall terminate at the lophophoral base. Duplicature bands visible. **b** Overview of autozooid musculature. Four sets of duplicature bands extend distally into the origin area of the parieto-vestibular muscles at the body wall. The parietal muscles extend from the oral to the anal side. Striated longitudinal tentacle muscles extend distally from the lophophoral base. **c** Detail of midgut. Arrangement of circular and longitudinal muscles including cardiac constrictor consisting of concentrated circular muscles. **d** Close up of foregut. Buccal dilatators extend from the pharynx and to the frontal lophophoral base muscle. **e** Detail of apertural muscles showing four bundles of thick parieto-vestibular muscles and circular vestibular wall muscles. Diaphragmatic sphincter muscle at the transition from vestibular wall to tentacle sheath.** f** Close up of gut. Funicular muscle extending over the caecum area, attaching it to the body wall. **g** Details of foregut. ‘V’ shaped lophophoral base muscles located distally to the abfrontal lophophoral base muscle. Circular frontal lophophoral base muscle. Striated muscles of the pharynx and oesophagus** h** Foregut. Transitions from mouth opening to midgut with cardiac constrictor highlighted.** i** Apertural muscles. Sets of duplicature bands. Parietal muscles of the body wall. Abbreviations: al – abfrontal lophophoral base muscle, bd – buccal dilatators, cae – caecum, cm – circular muscle, cst – cardiac constrictor, db – duplicature bands, ds – diaphragmatic sphincter, es – oesophagus, fl – frontal lophophoral base muscle, fm – funicular muscle, lb – lophophoral base, lm – longitudinal muscle, mo – mouth opening, pd – parieto-diaphragmatic muscle, ph – pharynx, pm – parietal muscle, pvm – parieto-vestibular muscle, rm – retractor muscle, ttm – tentacle muscle, tsm – tentacle sheath muscle, vm – ‘v’ shaped lophophoral base muscles, vwm – vestibular wall muscle

The lophophoral base is circular, and individuals have between 8 and 10 tentacles, depending on the species (Figs. 6c, e, g, and 10). The cerebral ganglion is located at the lophophoral base (Fig. 6d). The U-shaped digestive tract is generally divided into three sections starting with the mouth opening in the lophophore base and terminating at the anus. The foregut is comprised of the mouth opening, pharynx, oesophagus (Fig. 13h). The midgut consists of the cardia, caecum, and pylorus (Figs. 7 and 11). The intestine and anus make up the hindgut (Fig. 7f–h). The mouth opening extends into a triradial-shaped pharynx characterised by myoepithelial cells and follows into an elongated oesophagus (Fig. 6b). The proximal end of the elongated tubular cardiac region contains a cardiac constrictor (see Digestive tract muscles), and a large bulbous caecum follows generally bending in either direction depending on the orientation of the zooid (Figs. 7 and 11). Funicular muscles attach the caecum to the body wall. The pylorus forms the last part of the midgut and enters the intestine, which terminates with a lophophoral anus on the tentacle sheath (Figs. 7h and 10a). Termination of the anus may be near the lophophoral base or mid-positioned on the tentacle sheath between the lophophoral base and vestibular wall (Figs. 7c, d, g, h and 11c, e).

## Muscular system

### Body wall muscles

Transverse parietal muscles, lateral to the polypide, traverse the body cavity of each zooid in oral-anal direction. These muscle bundles are thick, and the number of muscles and the positions along the length of the polypide are highly variable (Fig. 13b, g, and i). They may occur on the oral side lateral to the tentacle sheath or on the sides (oral to anal direction of the body wall), or shifted to one side depending on the angle of the zooid. Muscular structures are also observed in the special cells embedded in pore plates (Fig. 5h–j).

### Apertural and tentacle sheath muscles

These muscles are associated with the opening and closing of the aperture of a zooid. Vestibular wall muscles consist of circular muscles used for contracting and enclosing the vestibular wall after polypide retraction. At the frontal end of each zooid four bundles of thick parieto-vestibular muscles originate from the disto-lateral body wall and attach at several areas of the vestibular walls (Fig. 13a). Proximally of the latter, two pairs of parieto-diaphragmatic muscles originate from a similar area and attach at the diaphragm, which carries a diaphragmatic sphincter separating the vestibulum from the atrium, the cavity enclosed by the retracted tentacle sheath (Fig. 13e). The latter is also supplied by simple longitudinal fibres (Fig. 13b and g). Four sets of duplicature bands, peritoneal muscular bands, originate from the vestibular area of the tentacle sheath and extend distally in retracted zooids to attach approximately at the level of the other prominent apertural muscles (Fig. 13a, b, and i).

### Digestive tract muscles

Prominent striated and circular muscles are characteristic in the foregut, with a characteristic myoepithelial pharynx (Fig. 13h). The oesophagus is elongated and characterized by a less dense net of circular muscles embedded with sparse smooth longitudinal muscles (Fig. 13c). All studied immergentiids possess a cardiac constrictor, a thick band of densely packed circular muscles, at the transition from the oesophagus to the cardia (Fig. 13c, d, h). The muscle net at the caecum and the intestine consists of loosely arranged muscles with either circular or longitudinal muscle fibres dominating in these areas, respectively. One or more funicular muscles attach the caecum to the body wall (Fig. 13f).

### Lophophoral muscles

Lophophoral muscles include tentacular muscles and lophophoral base muscles. Each tentacle has a frontal and abfrontal muscle consisting of longitudinal and striated fibres. There are four muscle sets at the lophophoral base: Abfrontally, most proximal lies the abfrontal lophophoral base muscle consisting of short longitudinal fibres extending in proximo-distal direction (Fig. 13g). Distally of the latter are ‘V’-shaped lophophoral base muscles which were traditionally termed v-shaped but in *Immergentia* are present as small muscular, mostly oblique short fibres (Fig. 13c, f and g). On the frontal side, a circular base muscle is located at the proximal border, the frontal lophophoral base muscles (Fig. 13g). Buccal dilatators are smooth muscle fibres that emerge from the pharynx towards the lophophoral base, terminating or overlapping at the frontal lophophoral muscle. Each dilatator corresponds to the base of a single tentacle (Fig. 13d).

### Retractor muscles

Smooth retractor muscles originate from the proximal tip of the cystid wall and attach at the lophophoral base and foregut areas (Fig. 13a, b and g). The arrangement consists of numerous thick longitudinal muscle fibres which may vary between individuals.

## Phylogenetic analysis

**Fig. 14:**
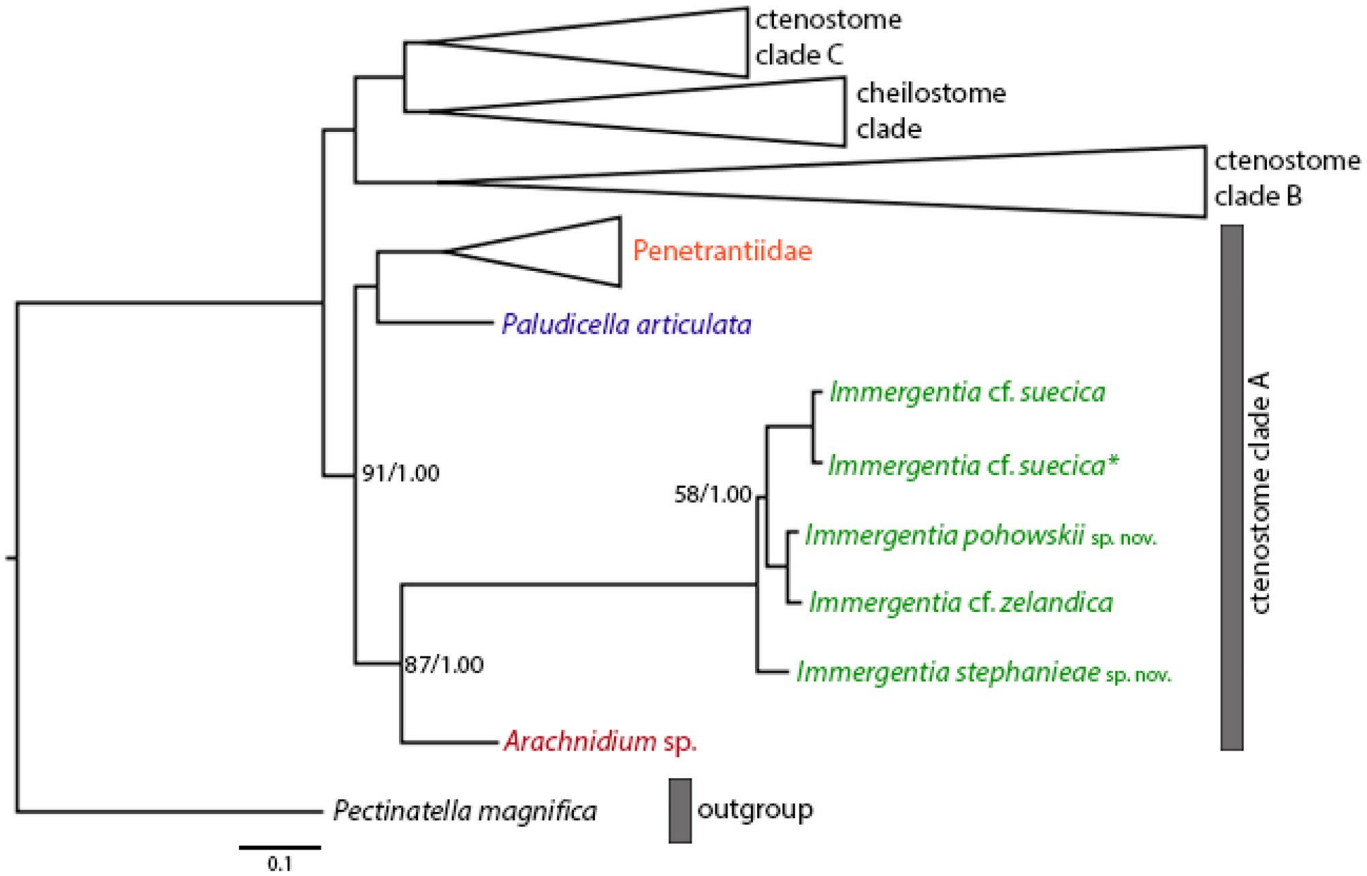
The inferred phylogeny of Immergentiidae based on a concatenated data matrix of 16 genes (12 PCGs, two ribosomal RNA genes (12S, 16S) and 2 nuclear ribosomal RNA genes (18S, 28S)). Maximum Likelihood (ML) phylogeny of ctenostomes incorporating nine cheilostomes (Orr et al., 2021), twenty-seven ctenostomes (Decker et al., 2024), as well as the freshwater *Pectinatella magnifica* Leidy, 1851 (Gim et al., 2018) used as an outgroup. Values on nodes represent branch support (ML bootstrap support (1000 replicates) and Bayesian posterior probabilities (based on last 75% of trees), respectively). Bootstrap support and Bayesian posterior probabilities are only shown for nodes that are not fully supported by all analyses. The scale bar represents 1 substitutional change per 100 characters

We sequenced and assembled the mitogenomes and two nuclear ribosomal genes (18S and 28S) of five immergentiids and incorporated them in a pre-existing broad phylogeny of ctenostomes (Decker et al., 2024). Our concatenated data matrix included 16 genes (12 PCGs and four ribosomal genes) totalling 9721 characters (2841 amino acids and 6880 nucleotide sites). The atp8 gene was excluded from our data matrix as it is missing in several samples. Both of our phylogenetic analyses (BI and ML) based on the concatenated dataset resulted in similar tree topology (ML, Fig. 14 and BI, see Data availability and materials section). Immergentiidae is fully supported as monophyletic group (100 bootstrap (BS) and 1.00 Posterior Probability (PP)). Within Immergentiidae, *I. stephanieae* sp. nov. was recovered as a sister taxon to all other immergentiids in the tree. Likewise, *I. pohowskii* sp. nov. and *I*.cf. *zelandica* were fully supported as sister taxa, collected from different locations and depths in the subtidal zone off the New Zealand coast. Similarly, both samples of *I.* cf. *suecica* from the subtidal zone off the North Eastern Atlantic Ocean along the English Channel were fully supported as a sister taxon.

*Arachnidium* sp. represented the sister-group to Immergentiidae. Both of these taxa have a sister group relationship with a clade comprising *Paludicella articulata* and Penetrantiidae, another boring bryozoan family. Regarding the genetic distance analysis, *I. stephanieae* sp. nov. has the greatest genetic distance to *I.* cf. *suecica*, then *I.* cf. *zelandica* and *I. pohowskii* sp. nov., at 27.8%, 19.3%, and 19.4% respectively (Table 2).

**Table 2:** Genetic distances of *cox1* (raw nucleotides) from MITOS annotation. Estimates of evolutionary divergence between Immergentiidae sequences. The number of base substitutions per site from between sequences is shown

***Immergentia* species**	*I.* cf.* suecica*	*I. stephanieae* sp. nov.	*I. cf. zelandica*	*Immergentia* sp.
*I.* cf*. suecica*	-			
*I. stephanieae* sp. nov.	0.278			
*I. cf. zelandica*	0.246	0.193		
*I. pohowskii* sp. nov.	0.249	0.194	0.108	-

## Discussion

### General morphology and colony form of immergentiids

The primary cystid appendage (associated with the apertural region) is the main connection that extends laterally from the ancestrula (initial growth direction), with zooids developing almost vertically at either regular intervals as seen in *I. stephanieae* sp. nov*.* or irregularly as in *I.* cf. *suecica*. Secondary cystid appendages or intercalary kenozooids interconnect with other cystid appendages to create box-like geometric shapes in all species. The colony morphology of *I.* cf. *suecica* from Norway and France resembles that of *I. suecica* from Sweden first described by Silén (1947). However, we could not verify this pattern from the type material because the substrate was fragile, eroded, and not easy to distinguish interconnections from the shell surface. Zooids of *I. pohowskii* sp. nov. were regularly spaced in some fragments but also overgrown in other colonies and therefore challenging to determine. According to Silén (1947) and Soule (1950), the zooids of *I. zelandica* are compactly and irregularly placed, and in dense colonies such as *I.* cf. *zelandica*, it is difficult to establish the pattern in relation to the primary cystid appendage (Fig. 9c). Establishing the colony form would require examination of colonies in early astogeny.

The morphology of the borehole apertures, typical spindle shape and enantiomorphy, is essential to distinguish *Immergentia* species from other boring ctenostomes. Determining the interzooidal intervals is also a valuable diagnostic characteristic. For example, scaled illustrations of the type *I. suecica* by Silén (1947) revealed that interzooidal intervals and zooid sizes were comparable to *I.* cf. *suecica* from France and Norway. Interzooidal intervals were similar in *I. pohowskii* sp. nov. from New Zealand. The interzooidal intervals of *I. stephanieae* sp. nov. are about one-third of those in *I.* cf. *suecica*; hence, the colony is considered to be dense. In this study, *I. patagoniana* is the only subtidal species exhibiting dense colonies with intervals of approximately 268 µm for the former. Colony form and interzooidal intervals could not be determined for *I.* cf. *zelandica* because zooids were positioned compactly, and consecutive cystid appendages were not apparent.

### Apertural area

The borehole apertures of immergentiids can be either circular, oval, or spindle shaped and display enantiomorphism or not, particularly when spindle shaped. The borehole apertures of *I. patagoniana* is one extreme with a strongly enantiomorphic spindle shaped borehole aperture, more prominent than all studied material and those from previous studies (see Pohowsky, 1978, pl. 22–24; this study Fig. 9a).* Immergentia stephanieae* sp. nov. displays the typical spindle shaped or sometimes oval borehole aperture and is weakly enantiomorphic, in the former. This is not easily distinguished in species with oval or circular borehole apertures like *I.* cf. *zelandica* and *I. zelandica*. The width of the borehole aperture was determined, but length was not considered because of inconsistencies due to difficulty in establishing the greatest length due in part to shell erosion, thus exposing the cystid appendages, risking measuring beyond the zooidal apertural area, and differing shapes of apertures. This problem was also highlighted by Pohowsky (1978).

There seems to be a slight difference in the length of the vestibulum (proportionally to the overall zooid length) depending on the state of the zooid. In autozooids the vestibular wall appears shorter approximately 18% of the total autozooid length, while in reproductive zooids it is 29%. Perhaps this distinction exists to facilitate eventual larval release in reproductive zooids. Vestibulum length can be difficult to determine, especially in zooids where the vestibular muscles obscure the vestibulum. An inconspicuous collar is observed in serial sections of some immergentiids but presumed to be present in all. The difficulty in observing this is attributed to the thin cuticular folds of the collar which is difficult to stain and dependent on the angle and thickness of serial sections.

### Cuticle and interzooidal pores

Arachnidioidea, a superfamily comprised of families such as Nolellidae, Arachnidiidae and Immergentiidae, are unified by representative species that possess anastomosing cystid appendages (Jebram, 1973; Schwaha, 2020a). In immergentiids, the primary cystid appendages often extend laterally from the zooidal apertural area and seldom on the proximo-distal axis, thus far only seen in few zooids of* I. stephanieae* sp. nov. This differs from the condition in terebriporids where the zooids lay parallel on the primary stolon with tubulets extending frontally from the zooids (see Pohowsky, 1978). Depending on the position of zooids within the colony and/or how are interconnected, a single primary cystid appendage may be present supported by secondary cystid appendages in immergentiids. Furthermore, if two of the latter emerge laterally from opposite sides of the zooid, they often occur in the same levelled plane, also depicted in *I. zelandica* by Silén (1947, fig. 64, p.43). A secondary cystid appendage arising from mid length of one zooid may directly attach to the primary cystid appendage of a neighbouring zooid. Secondary cystid appendages function as additional pathways for the exchange of dissolved substances and connections between zooids (Pohowsky, 1978). It should be noted that Pohowsky’s description of ‘paired lateral stolons’ in *I. patagoniana*, are associated with the primary cystid appendage (see Pohowsky, 1978).

The size and number of cystid appendages and additional thin processes in the studied samples do not appear to be species specific but rather random, offering support and stability to the colony. Thus, the characteristics of a substrate may influence these features. Two primary cystid appendages are common in immergentiids but a single one is also possible depending on the connections and position in the colony. More than two secondary cystid appendages were commonly observed in *I. stephanieae* sp. nov. and *I. pohowskii* sp. nov. but not exceeding four. In contrast, up to 9 cystid appendages had been described for *Arachnidium fibrosum* (see Marcus, 1938; Schwaha & De Blauwe, 2020). It is difficult to ascertain whether the presence or absence of tubulets is a valuable diagnostic characteristic for immergentiids. Thus far, they have been observed in all examined material. Their function is to exchange dissolved substances between the cystid appendages/stolons and the surrounding water environment (Marcus, 1938; Silén, 1947). In immergentiids, tubulets do not have septa as in Decker et al. (2023); however, in-depth examinations are required to be absolutely certain.

This study confirms that Silén’s (1947) assessment of ‘threads’ extending from the zooids are indeed cystid appendages and a ‘transverse wall’ separate neighbouring zooids. The latter refers to an interzooidal septum which forms at the junction of the cuticles of neighbouring zooids. Here, we report the cuticle, at the proximal end of a primary cystid appendage of the adjacent zooid, is separated by a pore plate displaying an uniporate condition. The report of the absence of a pore plate by Schwaha (2020a) is erroneous. Typically, the interzooidal pore complexes show the typical arrangement of special cells, cincture cells, and limiting cells (Bobin, 1977; Mukai et al., 1997; Schwaha, 2020b) but these were not distinguishable in this study. Here, the special cell, pore opening, and possibly limiting cells are observed but not cincture cells. This situation is not uncommon, as only special cells and pore opening could be determined in penetrantiids (see Decker et al., 2023).

## Zooidal polymorphs

### Intercalary kenozooids

Although cystid appendages connect zooids directly to each other, an intercalary kenozooid can also insert in the primary and secondary cystid appendage positions, a character not previously reported for immergentiids. Apart from the musculature associated with the septum, intercalary kenozooids do not contain any musculature. Granules, resembling those in sac zooids, stained positively for f-actin elements within the stolonal network of penetrantiids (Decker et al., 2023); however, such granules were not observed in the intercalary kenozooids or cystid appendages of immergentiids in this study.

### Sac zooids

Pohowsky (1978) used the term “sac zooids” to refer to “cénozoécie à sphérules” described for *Spathipora comma* by Bobin and Prenant (1954). These structures were observed in recent and fossil boring species (see Pohowsky, 1978; Schwaha, 2020a; Decker et al., 2023) but also in two non-boring ctenostomes (see Pohowsky, 1978). In *Immergentia*, sac zooids are true non-pedunculate polymorphs with a septum separating their cystid appendages from that of a neighbouring zooid or intercalary kenozooid. On the contrary, sac zooids in *Penetrantia* form part of the stolon and are not separated by septa (see Decker et al., 2023). Though the function of sac zooids remains unresolved (Bobin & Prenant, 1954; Pohowsky, 1978), we postulate that the granules possibly contain ‘reserve material’ for the colony or itself can become an autozooid under certain conditions. Sac zooids seem to be randomly dispersed throughout the colony to a lesser degree than autozooids. They also have varying shapes. In *I. patagoniana*, sac zooids are more rounded in shape (bulb shaped) while they vary (having both bulb and/or vase shapes) in colonies of all other immergentiids in this study.

### Autozooids and reproductive zooids

Generally, immergentiid autozooids are cylindrical/vase shaped with some variation in form. Autozooids of *I.* cf. *zelandica* closely resemble the description of *I. zelandica* (see Silén, 1947). The only noteworthy attribute reported for *I. zelandica* was that the abanal part of the basal end is drawn out into a narrow projection referred to as a finger-shaped projection by Silén (1947, fig. 63) and Soule (1950, mentioned but not illustrated). López-Gappa (1981, fig. 3) depicts a short projection at the basal tip of *I. zelandica patagonica* also observed in type material of *I. zelandica* in this study. This short projection differs from the few zooids of *I. pohowskii* sp. nov. and *I.* cf. *zelandica* that narrow to one side (pinch around the mid region) and elongate toward the basal end similar to the “finger-like projections” described and illustrated for the gonozooids of *Penetrantia densa* (see Silén, 1947). Finger-like projections are also described for *Sundanella* species (Schwaha et al., 2022). However, using the term “finger-like projections” for immergentiids is misleading therefore, this term is omitted as part of the description for immergentiid zooids.

Whether the pinch in the mid-zooidal area (seen in *I.* cf. *zelandica* and *I. pohowskii* sp. nov.) is a phenotypic characteristic of individuals in these colonies or a consequence of changes during dissolution of the shell/substrate (borehole formation) is debatable. It should however be noted that this attribute has not been observed in other immergentiids examined in this study. Another peculiarity is the acute curvature displayed in some zooids of *I. patagoniana* but a diagnostic character in *I. angulata* and *I. subangulata*. We observe that the placement of structures such as embryo in reproductive zooids, accumulation of brown bodies, and extent of the caecum or reproductive tract can slightly alter the shape of the zooid, giving the appearance of bulges. These scenarios were accounted for by examining several individuals in a colony, thus defining the typical zooidal shape.

To date, free-swimming larvae have not been described for immergentiids. Any attempts to retrieve already rarely seen larvae proved futile. Nonetheless, some valuable information was obtained from the material in this study. In these immergentiids, the embryo is brooded within the tentacle sheath (see Soule & Soule, 1969; Silén, 1947; Schwaha, 2020a) subsequently developing into a lecithotrophic coronate larva. Larvae are released from the tentacle sheath via the vestibulum and through the orifice in the apertural region. In this study, the term reproductive zooid is used to refer to the degenerated autozooid where the embryos are brooded; these are not gonozooids, i.e. a specialized zooid for reproduction. With the exception of *I. cheongpodensis* (see Seo et al., 2018) gonozooids in immergentiids have not been reported. Noteworthy is that *I. cheongpodensis* essentially represents an ichnospecies as the species description was based solely on the morphology of borehole apertures and resin casts of zooids (see Pohowsky, 1974; Bertling et al., 2006). Consequently, the absence of soft body morphology makes it challenging to ascertain the gonozooids of *I. cheongpodensis* to that of other recent boring ctenostomes namely *Penetrantia* (see Decker et al., 2023), *Spathipora elegans* and the fossil *S. cheethami* (see Pohowsky, 1978).

### Lophophore and digestive tract

The lophophore of all immergentiids is circular with varying numbers of tentacles. *Immergentia pohowskii* sp. nov. has 8 tentacles, the smallest number in this study. Prior to this study only *I. angulata* (Soule, 1950) from the Hawaiian Islands had 8 tentacles. However, a diagnostic feature of this species is that their zooids are acutely bent towards the primary cystid appendage, a feature not observed in *I. pohowskii* sp. nov. *Immergentia patagoniana*, *I.* cf. *suecica* and *I.* cf. *zelandica* typically have 9 tentacles.* Immergentia stephanieae* sp. nov. is the only immergentiid with autozooids that have either 9 or 10 tentacles within the same colony. Varying number of tentacles within the same colony is common in other bryozoans (see Thorpe et al., 1985; Porter et al., 2000; Decker et al., 2021; Schwaha et al., 2022). The lophophoral base is broader in *I. stephanieae* sp. nov. compared to *I. patagoniana* and *I.* cf. *suecica.* The same holds true for *I. pohowskii* sp. nov., but in this species, the proportion of lophophore and digestive tract to the zooidal body cavity is relatively small. Thus, the lophophore and digestive tract are smaller compared to other immergentiids in this study. Another distinction is the transition between the pharynx and oesophagus, which is severely pinched in *I. pohowskii* sp. nov.

At the transition of the foregut and midgut lies a cardiac valve, a characteristic of all bryozoans (Schwaha et al., 2020). Immergentiids do not possess a distinct proventriculus as in *Hislopia* (see Schwaha et al., 2011) or penetrantiids (Decker et al., 2023). All immergentiids in this study possess a prominent band of tightly packed circular muscles called a cardiac constrictor in the cardiac region. It is located at the transition from the oesophagus to the cardia (see Digestive tract musculature section), resembling those of nolelliids (Schwaha & Wanninger, 2018). The caecum is typically bulbous, tilted to one side (of the cardia), and the pylorus and anus on the opposite side. Funicular muscles attach the caecum to the body wall.

According to Schwaha (2020c) the position where the anus terminates into the tentacle sheath is variable in ctenostomes with either a vestibular anus (close to the vestibular wall) or lophophoral anus (close to the lophophore base). With the exception of *I. pohowskii* sp. nov. where the lophophoral anus is located in the mid-lophophoral position, the anus typically terminates near the lophophoral base in all other immergentiids in this study (low-positioned lophophoral anus). This distinction is made because the anus does not terminate directly at the lophophoral base as in the *Aethozooides uraniae* condition (see Schwaha et al., 2019) but distal to the lophophoral base. Schwaha (2020c) reported that the immergentiid depicted by Prenant and Bobin (1956) has a vestibular anus, while *I. californic*a by Soule (1950) possesses a mid-positioned lophophoral anus. However, this is a contradiction because Prenant and Bobin (1956) show a redrawing of *I. californica* by Soule (1950) with the anus in the mid-lophophoral position, similar to *I. californica* described by Silén (1947). The vestibular anus position of *I. suecica* is inaccurate (also see Schwaha, 2020c) and has a lophophoral anus. *Immergentia* cf. *suecica* from France and Norway both have low-positioned anuses. The location on the anus of *I.* cf. *zelandica* in this study is uncertain because it was difficult to discern in histological sections, but *I. zelandica* is reported to be mid-lophophoral (see Schwaha, 2020c) based on Soule’s (1950) illustration. Investigation of soft body morphology of the types *I. californica* and *I. zelandica* was mostly unsuccessful because zooids were in such a bad state that it was difficult to obtain consistent serial sections and to discern structures apart from the tentacles. Therefore, the position of the anus could not be confirmed in the aforementioned specimen.

## Musculature

Descriptions of the muscular structure of immergentiids is lacking compared to that of penetrantiids. Silén (1946, 1947) identified three sets of muscles consisting of four groups of parieto-vestibular (parieto-vaginales) and parieto-diaphragmatic muscles in the apertural area, and retractor muscles. However, the myoanatomy of ctenostomes by Schwaha and Wanninger (2018) encapsulates the condition in immergentiids which is detailed here.

### Body wall muscles

The transverse parietal muscles that cross the body cavity are characteristic of all gymnolaemates with the function of protruding the retracted polypide. In immergentiids, these consist of thick muscle bundles along the body wall with variable arrangements ranging from serial or concentrated bundles, also described by Schwaha and Wanninger (2018) and Schwaha (2020b). The septa at the cystid appendage or intercalary kenozooid junction with that of a neighbouring zooid constitute special cells that are stained for f-actin.

### Apertural and tentacle sheath muscles

In the apertural region, circular vestibular wall muscles enable contracting and enclosing of the vestibular wall after retraction of the polypide. Silén (1947) expressed difficulty in distinguishing the four groups of parieto-vestibular and parieto-diaphragmatic muscles, which is valid considering that both muscle sets originate from the lateral body wall and can appear clumped depending on the zooidal condition (Fig. 13i). However, four bundles of thick parieto-vestibular muscles attach at the distal vestibular wall while two pairs of parieto-diaphragmatic attach at the diaphragm, resembling the condition in *Paludicella* (see Schwaha et al., 2011) and *Nolella* (Schwaha & Wanninger, 2018). A diaphragmatic sphincter separates the vestibulum from the atrium and could only be observed laterally with *Ortho Slice* projections in Amira. Longitudinal muscle fibres that cover the tentacle sheath extend distally and attach to the area of the distal apertural muscles. Similar to immergentiids, four bundled sets of duplicature bands (previously referred to as parieto-vaginal bands) were also reported for penetrantiids (Decker et al., 2023). However, in immergentiids, a set can consist of at least two or three duplicature bands seen in *I. stephanieae* sp. nov. and *I.* cf. *suecica* from France.

### Digestive tract muscles

The foregut musculature of immergentiids is characterized by circular and longitudinal muscles. Prior to observations by Schwaha (2020a) and confirmation thereof here, the presence of a cardiac constrictor had not been reported in immergentiids. While the cardiac constrictor is simply a compact band of circular muscles in immergentiids, these muscles are more conspicuous, bulbous, and voluminous in other ctenostomes with a proventriculus or gizzard (see Schwaha & Wanninger, 2018). For example, cardiac constrictors are present in representatives of Arachnidioidea such as *Cryptoarachnidium argilla* (see Banta, 1967), *Arachnoidella evelinae* (see Marcus, 1937), *Arachnidium fibrosum*, *Nolella dilatata* and *Nolella*-like representatives (see Schwaha & Wanninger, 2018). The caecum is characterized by sparse circular muscle fibres and the intestine by longitudinal muscle fibres. Up to two funicular muscles have been observed in immergentiids thus far, and they may be more or less conspicuous.

### Lophophoral muscles

In immergentiids the tentacle muscles are similar to that of other bryozoans, each consisting of a frontal and abfrontal muscle band, which can be longitudinal or striated fibres (Schwaha, 2020b). The lophophoral base is comprised of four muscle sets as reported. Distal to the lophophoral base muscles are the equivalent of v-shaped muscles in *Hislopia malayensis* (Schwaha et al., 2011), but in immergentiids, these are short muscles fibres similar to that reported for penetrantiids (see Decker et al., 2023). Furthermore, in immergentiids, the muscle fibres have a similar structure as demonstrated in *Paludicella articulata* consisting of a median proximal element with ‘horns’ extending distally from either side (Schwaha & Wanninger, 2018). A circular base muscle is located above the mouth opening at the frontal proximal border of the frontal lophophoral base muscles. In immergentiids, the buccal dilatators are smooth muscle fibres, whereas they are mostly striated in myolaemates (Schwaha, 2020b) and extend from the pharynx towards the lophophoral base, intersecting the frontal lophophoral muscle (Gordon, 1974; Schwaha & Wanninger, 2018). A single dilatator corresponds to the base of a single tentacle and facilitates feeding by expanding the pharyngeal volume of the myoepithelial pharynx (Schwaha et al., 2020).

### Retractor muscles

Similar to other ctenostomes, the retractor muscles originate from the proximal end of the body wall and attach at the lophophoral base (see Silén, 1947; Schwaha & Wanninger, 2018) and near the foregut in immergentiids. These muscle fibres are smooth and prominent in immergentiids, as in most ctenostomes, though striated fibres can also occur in some groups (Schwaha & Wanninger, 2018).

## Phylogenetic analysis

A novel phylogenetic placement for *Arachnidium* sp. as the sister taxon to Immergentiidae is recovered. This relationship based on molecular data supports early morphology-based phylogenetic inferences (see Jebram, 1973; Soule & Soule, 1975; Schwaha, 2020a; Schwaha & De Blauwe, 2020) between the two groups. Silén indicated that *Immergentia* is possibly closely related to *Nolella*, based primarily on the position of secondary cystid appendages in *N. annecten*s (see Silén, 1942, 1947); this feature however was not observed by Schwaha (2020a). The inclusion of more representatives of the aforementioned superfamily would improve phylogenetic inferences of this group.

Immergentiidae is fully supported as a monophyletic group in the ctenostome clade A by the BI, though not significantly supported by the ML. *Immergentia stephanieae* sp. nov. is recovered as a sister taxon to all other immergentiids, in the first divergence in the Immergentiidae. This species represents the only intertidal and morphologically distinct species to all immergentiids in this study. The genetic distance between the aforementioned species and *I.* cf. *suecica* is 27.8%, well above the 3% threshold suggested for delineation of species (see Baptista et al., 2022; Meyer & Paulay, 2005); however, more sampling of species from different geographic regions is required to establish a robust threshold for immergentiids. Two species from New Zealand *I.* cf. *zelandica* (inner Otago shelf) and *I. pohowskii* sp. nov. (Taiaroa Head) are considered to be distinct with a genetic distance of 10.8%, supported by morphological differences. They were collected from locations measuring approximately 3 km apart (diagonally) but varying in depth and habitat. Unfortunately, the genetic distance of the second *I.* cf. suecica* from France could not be determined because CO1 was not recovered in this taxon. These immergentiids were collected from different locations in the subtidal zones, Térenéz and Stolvezen near Roscoff, France (see Table S1).

The clade comprised of Immergentiidae and *Arachnidium* sp. form a sister group relationship with a clade consisting of *P. articulata* and Penetrantiidae. Silén enclosed *Immergentia* in Paludicelloidea stating that zooidal structure was similar to those of *Paludicella* (see Silén, 1942, 1947) and indeed shares similar features with other ctenostomes (see Schwaha, 2020a). However, *Paludicella* is distinctly different from *Immergentia* in that it is non-boring, one of the few ctenostomes with peritoneal cilia (Meyer, 1927; Mukai et al., 1997; Weber et al., 2014), lumen within the cerebral ganglion and two funiculi that connect the caecum (Schwaha & Wanninger, 2018; Weber et al., 2014). In addition, it is a freshwater species. It should be noted that at the time of Silén’s examinations, Paludicelloidea comprised the genera *Sundanella*, *Arachnoidea*, *Nolella*, and *Victorella* (see Silén, 1942), which has been revised (see Jebram, 1973; Schwaha, 2020a). Thus, a possible explanation for why he related *Immergentia* to both *Paludicella and Nolella.* Though Silén was correct in deducing that they probably had a common ancestor, *Paludicella* is closely related to and forms a sister taxon with *Penetrantia* (Decker et al., 2024); however, describing this relationship is beyond the scope of this manuscript.

## Substrate diversity

Immergentiids were abundant in molluscan shells (Tables 1 and S5). Substrates from locations of opportunity were limited, but from Burdwood Bank, about 30 bored gastropod shells half contained *Immergentia*, from Norway 1 shell out of 4, from New Zealand 11 of 18 fragments contained *Immergentia*. The number of samples from France outnumbered those from other locations since thousands of shells were examined. In shells that were bored, immergentiids were present in about 80%.

Colonies of *I. patagoniana* from the Burdwood Bank were found in dead gastropod shells occupied by the hermit crab *Pagurus comptus* White, 1847 collected from the subtidal zone at depths between 105 and 139 m (Table S1), while *I. stephanieae* sp. nov. was abundant in living gastropods, particularly *Littorina littorea* Linnaeus, 1758 in the intertidal zone in Roscoff, France. Furthermore, *I.* cf. *suecica* occupied shells of dead bivalves including *L. lutraria* and *Ensis* sp. sampled from the subtidal zone of Roscoff at approximate depths of 10 to 25 m. On the other hand, *I.* cf. *suecica* from Norway was only found in one shell of the gastropod *B. undatum* housing a hermit crab. The substrate fragments from New Zealand containing *I. pohowskii* sp. nov. (depth 23 m) and *I.* cf. *zelandica* (depth 40) could not be identified, however the latter was also obtained from a gastropod belonging to Buccinoidea Rafinesque, 1815.

Boring bryozoans are able to colonize calcium carbonate substrates by chemical dissolution. The suggestion that this is achieved by phosphoric acid secretion has been cast in doubt by Silén (1947) and Pohowsky (1978). *Immergentia stephanieae* sp. nov. was abundant in the shells of living gastropods *L. littorea* and, to a lesser extent *Nucella lapillus* Linnaeus, 1758 with colonies able to cover the entire substrate when heavily bored. Colonies can easily be seen if located in the apertural area (especially the inner lip) of the gastropod. There seems to be a preference for *L. littorea* because no colonies were found in other *Littorina* species commonly encountered in the intertidal zone around Roscoff such as *L. fabalis* W. Turton, 1825, *L. obtusata* Linnaeus, 1758, *L. saxatilis* Olivi, 1792, *L. compressa* Jeffreys, 1865 as well as *Gibbula* sp. Risso, 1826, whether alive or not. It should be noted that after the aforementioned assessment, collection bias was skewed based on the probability of finding living colonies on *L. littorea*. This gastropod may be a desirable substrate owing to its larger size (in comparison to the other *Littorina* species mentioned above), providing a larger surface area for immergentiid colony expansion. The composition of the substrate and ease/effort of dissolution may also play a role. In addition, *L. littorea* was commonly found in tide pools or under rocks or within *Fucus* leaves, thus minimizing desiccation exposure and ensuring nutrient availability for the immergentiids (in the tide pool habitat).

Comparatively, previous studies of immergentiids from the intertidal zones or tide pools were found in different species of living and/or dead gastropods (see Table S5). For example, the type of *I. californica* was present in living *Tegula brunnea* R. A. Philippi, 1849 (see Silén, 1947) and dead *L. scutulata* Gould, 1849 (see Soule, 1950), *I. zelandica* in living *Buccinulum littorinoides* Reeve, 1846 (see Silén, 1946) and *I. zelandica minuta* in living *Stomatella planulata* Lamarck, 1816 (see Soule, 1950). Though *I. cheongpodensis* was collected from the intertidal zone, the substrate fragments could not be identified (see Seo et al., 2018).

In previous studies immergentiids were extracted from living gastropods in the subtidal zone, for example*, I. patagoniana* in *Pareuthria fuscata* Bruguière, 1789 (see López-Gappa, 1981). In contrast, all the material of *I. patagoniana* from the Burdwood Bank occurred in hermited gastropod shells (see López-Gappa & Zelaya, 2021 and Table 1). Interestingly, a syntype of *Terebripora orbignyana* included an unidentified bivalve bearing a recent *I. orbignyana* from the south-west coast Arcachon, France (see Fischer, 1866; Pohowsky, 1978). However, further details of collection and characteristics of morphology are absent. In this study, immergentiids were not collected from living molluscs from the subtidal zone in Roscoff, France. Instead, borings were commonly found in slightly or completely eroded shells of dead bivalves, which are present in this habitat, such as but not limited to *Ensis* sp., *L. lutraria*, *P. maximus*, and *Anomia* sp.

## Challenges and general remarks

### Sampling and fixation

Compared to other boring bryozoans, *Immergentia* have fewer characteristics available for species determination. Silén (1947) expressed a lack of “remarkable features” in immergentiids with the exception of tentacle numbers. Similarly, Soule and Soule (1969) reported that *I. angulata* for example did not “possess many characteristics to distinguish it from other immergentiids or even terebriporids”. The absence of soft-body morphology makes this task even more challenging. Owing to the difficulty of identifying immergentiids from borings and colony morphology alone, specimens from other locations where representatives have been reported, requires confirmation of species.

Though shells from Sweden (type locality for *I. suecica*) were examined in this study, no *Immergentia* borings were found but those of *Penetrantia* were abundant. Silén reported the difficulty of finding *Immergentia* compared to *Penetrantia concharum,* which was abundant in shells of the pectinid *Pseudamussium peslutrae* Linnaeus, 1771. He only managed to extract one large and two small zooids successfully for examination. Silén’s descriptions and drawings were valuable in assessing the species; we could measure zooid size, interzooidal intervals, and colony form from scaled drawings. Therefore, we could tentatively place *Immergentia* from France and Norway, though there were limitations as well. This study presents a better idea of variation in zooid size with more zooids measured than in previous studies. In each of the aforementioned instance, zooid sizes were determined from less than five zooids thus information on variation in zooid sizes were limited.

The fixative used to preserve a specimen influences subsequent analysis. For example, samples from the Burdwood Bank and Norway were initially fixed in formalin, which allowed for histology but not DNA extraction. Similarly, *I. suecica* was fixed in Bouin’s fluid (consists of acetic acid, picric acid, and formaldehyde) and variations of Flemming’s strong and weak solution (contains acetic acid, chromium trioxide, and osmium tetroxide) which work well for histology but disrupts DNA. In addition, Silén (1947) dissolved the substrates with either acetic acid or nitric acid (concentrations not known) which compromised the integrity of the substrate and possibly the condition of the *Pecten* shell fragments with a *I. suecica* colony. Furthermore, the storage solutions may also affect the conditions of the zooids, especially those that are stored in a decalcified state over time.

The condition of the zooids also determines the kind of data that can be extracted, as sometimes staining does not work or issues are encountered during histological analysis. If zooids are in a deteriorated or degenerated state, the structures cannot be assessed. Hence, a high volume of material needs to be examined and analysed to obtain data. In addition, access to and condition of type material is an additional barrier to determining species and their characteristics.

This study presents a new standard to identify immergentiid species; therefore, there is a need to re-examine type material, in particular, especially in the species where there are no data of soft-body morphology.

### The position of *Immergentia philippinensis*

In his 1978 monograph, Pohowsky already raised concerns about the placement of *I. philippinensis*, a notion supported in this study. Soule’s species description of the zooid orientation, growth pattern, and colony form in plate 2, Fig. 3 (1950) resemble that of *Terebripora.* The statement that ‘zooids lay in a horizontal plane, parallel to the stolon directly above it’ (Soule, 1950) is particularly striking. In fact, the descriptions differ from those of *I. zelandica* var *minuta* in the same study. Whether or not the soft body morphology in plate 1 (Fig. 5) is based on the same colony (which seems to be the case here), the description of the species is not that of an immergentiid. Instead, the soft body morphology of zooids from this *Terebripora* species probably resembles that of *Immergentia*, the author provides drawings of zooids from the respective colonies (see Soule, 1950 plate 1, images 5 and 6). This notion was also expressed by Markham and Ryland (1987). After assessing the aforementioned figures, it seems that the digestive tract differs from the material in this study and the histological drawing of *I. californica* by Silén in 1947. Beyond the cardia, the elongated shape and orientation of the caecum (labelled as stomach in Soule, 1950) in relation to the pylorus (rectum, Soule, 1950) and anus are in the same plane. One could argue that this could have been done to easily depict all the structures. Needless to say, further investigations are required to confirm the affinities of *I. philippinensis* and/or the soft body morphology of *Terebripora*.

## Conclusions

This study presents valuable details about the soft body morphology of immergentiids. For the first time, it is shown that *Immergentia* possess both cystid appendages, intercalary kenozooids as well as a cardiac constrictor, prompting the amendment of the family characteristics. Delving into the morphology has allowed comparisons and differences to be drawn with other ctenostomes. The successful extraction of DNA enabled the determination of the mitogenome of immergentiids, relating the phylogenetic relationship with other ctenostomes. The phylogenetic affinity of Immergentiidae to arachnidiids is supported by this work. We have established a valuable tool and standard for future species identification by combining the descriptions of borehole apertures, interzooidal spaces, zooid and colony dimensions and morphology, and merging illustrations from early research with histology, confocal scans, and 3D reconstructions of zooids. However, broader sampling and further analysis are still required to determine the true diversity of immergentiids.

### Supplementary Information

Below is the link to the electronic supplementary material.

Supplementary file1 (DOCX 5930 KB)

## Data Availability

The data analysed during this study are available from the corresponding author upon reasonable request. Sequences generated in our study are available from NCBI GenBank BioProject PRJNA1051543. Sequences produced by other authors were sourced from publicly available data on the GenBank database: https://www.ncbi.nlm.nih.gov/genbank/. This article is registered in ZooBank under: urn:lsid:zoobank.org:pub:B7E5F369-F8EA-47F9-AC8A-3C2C0B8C8E23.

## References

[CR1] Allman, G. J. (1856). *A monograph of the fresh-water polyzoa, including all the known species, both British and Foreign*. The Ray Society.

[CR2] Altschul SF, Gish W, Miller W, Myers EW, Lipman DJ (1990). Basic local alignment search tool. Journal of Molecular Biology.

[CR3] Bankevich A, Nurk S, Antipov D, Gurevich AA, Dvorkin M, Kulikov AS (2012). SPAdes: A new genome assembly algorithm and its applications to single-cell sequencing. Journal of Computational Biology.

[CR4] Banta WC (1967). A new species of Victorella from southern California (Bryozoa: Ctenostomata). Proceedings of the U.S. National Museum.

[CR5] Baptista L, Berning B, Curto M, Waeschenbach A, Meimberg H, Santos AM, Ávila SP (2022). Morphospecies and molecular diversity of ‘lace corals’: The genus *Reteporella* (Bryozoa: Cheilostomatida) in the central North Atlantic Azores Archipelago. BMC Ecology and Evolution.

[CR6] Bertling M, Braddy SJ, Bromley RG, Demathieu GR, Genise J, Mikuláš R, Nielsen JK, Nielsen KSS, Rindsberg AK, Schlirf M, Uchman A (2006). Names for trace fossils: A uniform approach. Lethaia.

[CR7] Bobin G, Woollacott RM, Zimmer RL (1977). Interzooecial communications and the funicular system. Biology of Bryozoans.

[CR8] Bobin G, Prenant M (1954). Sur un bryozaire perforant (*Terebripora comma* Soule), trouvé en Méditerranée. Archives De Zoologie Expérimentale Et Générale.

[CR9] Borowiec ML (2016). AMAS: A fast tool for alignment manipulation and computing of summary statistics. PeerJ.

[CR10] Bruguière, J. G. (1789). *Encyclopédie méthodique ou par ordre de matières: Histoire naturelle des vers* (Vol. 1). Pancoucke. 10.5962/bhl.title.49857 (Original work published 1789–1792).

[CR11] Busk G, MacGillivray J (1852). An account of the Polyzoa, and sertularian zoophytes, collected in the Voyage of the Rattlesnake, on the coasts of Australia and the Louisiade Archipelago. Narrative of the Voyage of the H.M.S. Rattlesnake.

[CR12] Capella-Gutiérrez S, Silla-Martínez JM, Gabaldón T (2009). trimAl: A tool for automated alignment trimming in large-scale phylogenetic analyses. Bioinformatics.

[CR13] Castellanos Z, Rolán E, Bartolotta S (1987). Nuevos micromoluscos de la plataforma inferior Argentina y talud superior (Moll. Gastropoda). Revista del Museo de La Plata Sección Zoología.

[CR14] Cocito S (2004). Bioconstruction and biodiversity: Their mutual influence. Scientia Marina.

[CR15] Criscuolo A, Gribaldo S (2010). BMGE (Block Mapping and Gathering with Entropy): A new software for selection of phylogenetic informative regions from multiple sequence alignments. BMC Evolutionary Biology.

[CR16] Cuffey RJ (1977). Bryozoan contribution to reefs and bioherms through geologic time. American Association of Petroleum Geologists Studies in Geology.

[CR17] Cuffey RJ (2006). Bryozoan-built reef mounds: The overview from integrating recent studies with previous investigations. CFS Courier Forschungsinstitut Senckenberg.

[CR18] Darriba D, Posada D, Kozlov AM, Stamatakis A, Morel B, Flouri T (2020). ModelTest-NG: A new and scalable tool for the selection of DNA and protein evolutionary models. Molecular Biology and Evolution.

[CR19] De Blauwe H (2009). Mosdiertjes van de Zuidelijke bocht van de Noordzee: Determinatiewerk voor België en Nederland.

[CR20] Decker SH, Gordon DP, Spencer Jones ME, Schwaha T (2021). A revision of the ctenostome bryozoan family Pherusellidae, with description of two new species. Journal of Zoological Systematics and Evolutionary Research.

[CR21] Decker SH, Hirose M, Lemer S, Kuklinski P, Spencer HG, Smith AM, Schwaha T (2023). Boring bryozoans: An investigation into the endolithic bryozoan family Penetrantiidae. Organisms Diversity & Evolution.

[CR22] Decker SH, Saadi A, Baranyi C (2024). Boring systematics: A genome skimmed phylogeny of ctenostome bryozoans and their endolithic family Penetrantiidae with the description of one new species. Ecology and Evolution.

[CR23] de Rochebrune AT, Mabille J (1885). Diagnoses de mollusques nouveaux, recueillis par les membres de la mission du Cap Horn et M. Lebrun, Préparateur au Muséum, chargé d'une mission à Santa-Cruz de Patagonie. Bulletin de la Société Philomathique de Paris.

[CR24] Dillwyn LW (1817). A descriptive catalogue of Recent shells, arranged according to the Linnaean method; with particular attention to the synonymy.

[CR25] d'Hondt JL (1983). Tabular keys for the identification of the Recent Ctenostomatous Bryozoa. Mémoires De L'institut Océanographique.

[CR26] Donath A, Jühling F, Al-Arab M, Bernhart SH, Reinhardt F, Stadler PF (2019). Improved annotation of protein-coding genes boundaries in metazoan mitochondrial genomes. Nucleic Acids Research.

[CR27] d’Orbigny A (1847). Voyage Dans L’amerique Meridionale. Zoophytes.

[CR28] Dutka TL, Fejer AJ, Williams T, Donnelly DM, Flynn AJ (2022). Extent and characteristics of a newly discovered unique Bryozoan biogenic reef complex. Frontiers in Marine Science.

[CR29] Fischer P (1866). Étude sur les Bryozoaires perforants de la famille des Térébriporides. Nouvelles Archives Du Museum D’histoire Naturelle Lyon.

[CR30] Gim J, Ko E, Kim H, Kim Y, Hong S, Kim H, Gim J, Joo G, Jo H (2018). Complete mitochondrial genome of the freshwater bryozoan *Pectinatella magnifica* (Phylactolaemata: Plumatellida) assembled from next-generation sequencing data. Mitochondrial DNA Part b, Resources.

[CR31] Gordon DP (1974). Microarchitecture and function of the lophophore in the bryozoan *Cryptosula pallasiana*. Marine Biology.

[CR32] Gould AA (1849). Descriptions of new species of shells, brought home by the U.S. Exploring Expedition. Proceedings of the Boston Society of Natural History.

[CR33] Harmer SF (1915). The polyzoa of the siboga expedition. Part 1. Entoprocta. Ctenostomata and Cyclostomata. Siboga Expedition Reports.

[CR34] Hayward PJ, Kermack DM, Barnes RSK (1985). Ctenostome Bryozoans: Keys and notes for the identification of the species. Synopses of the British Fauna.

[CR35] Hincks T (1880). A history of the British Marine Polyzoa.

[CR36] Huelsenbeck JP, Ronquist F (2001). MRBAYES: Bayesian inference of phylogenetic trees. Bioinformatics.

[CR37] Jebram D (1973). Stolonen-Entwicklung und Systematik bei den Bryozoa Ctenostomata. Journal of Zoological Systematics and Evolutionary Research.

[CR38] Jeffreys, J. G. (1865). *British conchology, or, An account of the Mollusca which now inhabit the British Isles and the surrounding seas* (Vol. 3). John Van Voorst. 10.5962/bhl.title.16342 (Original work published 1862–1869).

[CR39] Katoh K, Standley DM (2013). MAFFT Multiple Sequence Alignment Software Version 7: Improvements in performance and usability. Molecular Biology and Evolution.

[CR40] Kozlov AM, Darriba D, Flouri T, Morel B, Stamatakis A (2019). RAxML-NG: A fast, scalable and user-friendly tool for maximum likelihood phylogenetic inference. Bioinformatics.

[CR41] Kumar S, Nei M, Dudley J, Tamura K (2008). MEGA: A biologist-centric software for evolutionary analysis of DNA and protein sequences. Briefings in Bioinformatics.

[CR42] Lagesen K, Hallin P, Rødland EA, Staerfeldt HH, Rognes T, Ussery DW (2007). RNAmmer: Consistent and rapid annotation of ribosomal RNA genes. Nucleic Acids Research.

[CR43] Lamarck J. B. P. A. (1816). Liste des objets représentés dans les planches de cette livraison. In J.G Bruguière, B. de Saint.Vincent & O. T. Müller (Eds.), *Tableau encyclopédique et méthodique des trois règnes de la nature: Vers, coquilles, mollusques et polypiers* (Vol. 1). Agasse. (Original work published 1791–1827). 10.5962/bhl.title.63299

[CR44] Leidy J (1851). On *Cristatella magnifica* n. sp. Proceedings of the Academy of Natural Sciences of Philadelphia.

[CR45] Linnaeus, C. (1753). *Species plantarum :exhibentes plantas rite cognitas, ad genera relatas, cum differentiis specificis, nominibus trivialibus, synonymis selectis, locis natalibus, secundum systema sexuale digestas* (Tomus I). Holmiae: Impensis Direct, Laurentii Salvii. 10.5962/bhl.title.669

[CR46] Linnaeus, C. (1758). *Systema Naturae per regna tria naturae, secundum classes, ordines, genera, species, cum characteribus, differentiis, synonymis, locis* (10th revised edition, Vol. 1). Holmiae: Impensis Direct, Laurentii Salvii. 10.5962/bhl.title.542

[CR47] Linnaeus C. (1771). *Mantissa plantarum: Generum editionis VI. et specierum editionis II* (Mantissa 2). Holmiae: Impensis Direct, Laurentii Salvii. 10.5962/bhl.title.69083

[CR48] López-Gappa, J. J. (1981). Briozoos marinos de la Ria Deseado (Santa Cruz, Argentina). I. *Physis (Buenos Aires), (A)*, *39*, 23–32. http://www.marinespecies.org/aphia.php?p=sourcedetails&id=371018

[CR49] López-Gappa J, Zelaya DG (2021). Bryozoan assemblages on gastropod shells occupied by the hermit crab *Pagurus comptus*. Polar Biology.

[CR50] Marcus E (1937). Briozoarios marinhos brasileiros I. Boletim Da Faculdade De Filosofia, Ciências e Letras, Universidade De São Paulo, Zoologia.

[CR51] Marcus E (1938). Bryozoarios marinhos brasileiros. II. Boletim Da Faculdade De Filosofia, Ciências e Letras, Universidade Di Sao Paolo, Zoologia.

[CR52] Markham JB, Ryland JS (1987). Function of the gizzard in Bryozoa. Journal of Experimental Marine Biology and Ecology.

[CR53] Mayoral E (1991). Actividad bioerosiva de briozoos ctenostomados en el Ordovícico Superior de la Zona Cantábrica del Macizo Hespérico (Cabo Vidrias, Oviedo). Revista Española De Paleontología.

[CR54] Meyer A (1927). Über Cölombewimperung und cölomatische Kreislaufsysteme bei Wirbellosen. Ein Beitrag zur Histophysiologie der secundären Leibeshöhle und ökologischen Bedeutung der Flimmerbewegung. Zeitschrift Für Wissenschaftliche Zoologie.

[CR55] Meyer CP, Paulay G (2005). DNA Barcoding: Error rates based on comprehensive sampling. PLOS Biology.

[CR56] Mukai H, Terakado K, Reed CG, Harrison FW, Woollacott RM (1997). Bryozoa. Microscopic anatomy of invertebrates.

[CR57] Nascimento FF, Reis MD, Yang Z (2017). A biologist's guide to Bayesian phylogenetic analysis. Nature Ecology and Evolution.

[CR58] Olivi, G. (1792). *Zoologia Adriatica, ossia catalogo ragionato degli animali del golfo e della lagune di Venezia*. Bassano. 10.5962/bhl.title.60887

[CR59] Orr RJS, Sannum MM, Boessenkool S (2021). A molecular phylogeny of historical and contemporary specimens of an under-studied micro-invertebrate group. Ecology and Evolution.

[CR60] Philippi RA (1808). Abbildungen und beschreibungen neuer oder wenig gekannter conchylien, unter mithülfe mehrer deutscher conchyliologen.

[CR61] Philippi, R. A. (1849). Centuria tertia testaceorum novorum. *Zeitschrift für Malakozoologie, 5*(12), 186–192. (Original work published 1844–1854).

[CR62] Pohowsky RA (1974). Notes on the study and nomenclature of boring bryozoa. Journal of Paleontology.

[CR63] Pohowsky, R. A. (1975). Boring Bryozoa. In S. Pouyet (Ed.), *Bryozoa 1974*: *Documents des laboratoires de géologie de la Faculté des sciences de Lyon* (Hors série *3*(1), pp. 255–256)*.* Université Claude Bernard.

[CR64] Pohowsky, R. A. (1978). The boring Ctenostomate Bryozoa: Taxonomy and Paleobiology based on cavities in calcareous substrata. *Bulletins of American Paleontology*, *73*(301).

[CR65] Porter. J. S., Bloor, P., Stokell, B. L., & Ryland, J. S. (2000). Intra- and inter-specific variation in tentacle number in the genus *Alcyonidium* (Bryozoa, Ctenostomatida). In A. Herrera Cubilla, & J. B. C. Jackson (Eds.), *Proceedings of the 11*^*th*^* International Bryozoology Association Conference: Smithsonian Tropical Research Institute, Republic of Panama, January 26*–*31* (pp. 321–328). Smithsonian Tropical Research Institute.

[CR66] Prenant, M., & Bobin, G. (1956). Faune de France 60: Bryozoaires première partie entoproctes, phylactolèmes, cténostomes. In Lechevalier (Ed.), *Faune de France* (Vol. 60, pp. 398). Fédération Française des sociétés de sciences naturelles.

[CR67] Rafinesque. (1815). Buccinoidea. In MolluscaBase (Eds.) *Buccinoidea Rafinesque, 1815*. Retrieved July 31, 2023, from https://www.marinespecies.org/aphia.php?p=taxdetails&id=382214

[CR68] Reeve, L. A. (1846). Monograph of the genus *Buccinum*. In: *Conchologia Iconica, or, illustrations of the shells of molluscous animals* (Vol. 3, pl 1–12). Lovell, Reeve. 10.5962/bhl.title.8129. (Original work published 1845–1847).

[CR69] Reverter O, d’Hondt JL, Fernandez E (1995). Mise à jour de l’inventaire des Bryozoaires de Roscoff publié par Echalier et Prenant (1951). Cahiers De Biologie Marine.

[CR70] Reverter-Gil O., Souto J. & Fernández-Pulpeiro E. (2016). *Bryozoa I. Ctenostomata*. Fauna Ibérica (Vol. 43, pp. 1–305). Museo Nacional de Ciencias Naturales.

[CR71] Risso, A. (1826). *Histoire naturelle des principales productions de l'Europe Méridionale et particulièrement de celles des environs de Nice et des Alpes Maritimes* (Vol. 1–5). F.G. Levrault. 10.5962/bhl.title.58984 (Original work published 1826–1827).

[CR72] Rosso A (2003). Bryozoan diversity in the Mediterranean Sea. The Journal of Integrative Biogeography.

[CR73] Rosso A, Di Martino E (2016). Bryozoan diversity in the Mediterranean Sea: An update. Mediterranean Marine Science.

[CR74] Ruthensteiner B (2008). Soft Part 3D visualization by serial sectioning and computer reconstruction. Zoosymposia.

[CR75] Schindelin J, Arganda-Carreras I, Frise E, Kaynig V, Longair M, Pietzsch T, Preibisch S, Rueden C, Saalfeld S, Schmid B, Tinevez J-Y, White DJ, Hartenstein V, Eliceiri K, Tomancak P, Cardona A (2012). Fiji: An open-source platform for biological-image analysis. Nature Methods.

[CR76] Schumacher CF (1817). Essai d'un nouveau système des habitations des vers testacés: Avec XXII planches. Schultz.

[CR77] Schwaha T, Schwaha T (2020). Ctenostomata. *Handbook of Zoology: Phylum Bryozoa*.

[CR78] Schwaha T, Schwaha T (2020). Morphology of bryozoans. *Handbook of Zoology: Phylum Bryozoa*.

[CR79] Schwaha T (2020). O anus, where art thou? An investigation of ctenostome bryozoans. Journal of Morphology.

[CR80] Schwaha T, Bernhard JM, Edgcomb VP, Todaro MA (2019). *Aethozooides uraniae*, a new deep-sea genus and species of solitary bryozoan from the Mediterranean Sea, with a revision of the Aethozoidae. Marine Biodiversity.

[CR81] Schwaha T, De Blauwe H (2020). Morphology of ctenostome bryozoans: 1. *Arachnidium fibrosum*. Journal of Morphology.

[CR82] Schwaha TF, Ostrovsky AN, Wanninger A (2020). Key novelties in the evolution of the aquatic colonial phylum Bryozoa: Evidence from soft body morphology. Biological Reviews.

[CR83] Schwaha TF, Wanninger A (2018). Unity in diversity: A survey of muscular systems of ctenostome Gymnolaemata (Lophotrochozoa, Bryozoa). Frontiers in Zoology.

[CR84] Schwaha T, Winston JE, Gordon DP (2022). Morphology of ctenostome bryozoans: 5. Sundanella, with description of a new species from the Western Atlantic and the Multiporata concept. Journal of Morphology.

[CR85] Schwaha T, Wood TS, Wanninger A (2011). Myoanatomy and serotonergic nervous system of the ctenostome *Hislopia malayensis*: Evolutionary trends in bodyplan patterning of Ectoprocta. Frontiers in Zoology.

[CR86] Senowbari-Daryan B, Zuehlke R, Bechstädt T, Flügel E (1993). Anisian (Middle Triassic) buildups of the Northern Dolomites (Italy): The recovery of reef communities after the Permian/Triassic crisis. Facies.

[CR87] Seo JE, Chae HS, Winston JE, Zágoršek K, Gordon DP (2018). Korean ctenostome bryozoans-observations on living colonies, new records, five new species, and an updated checklist. Zootaxa.

[CR88] Sharples A, Huuse M, Hollis C, Totterdell J, Taylor P (2014). Giant middle Eocene bryozoan reef mounds in the Great Australian Bight. Geology.

[CR89] Silén L (1942). Origin and development of the Cheilo-Ctenostomatous stem of Bryozoa. Zoologiska Bidrag Från Uppsala.

[CR90] Silén L (1946). On two new groups of Bryozoa living in the shells of molluscs. Arkiv För Zoologi.

[CR91] Silén L (1947). On the anatomy and biology of Penetrantiidae and Immergentiidae (Bryozoa). Arkiv För Zoologi.

[CR92] Slater GS, Birney E (2005). Automated generation of heuristics for biological sequence comparison. BMC Bioinformatics.

[CR93] Smith, E. A. (1881). Account of the zoological collections made during the survey of the H.M.S. 'Alert' in the Straits of Magellan and on the coast of Patagonia. IV. Mollusca and Molluscoidea. *Proceedings of the Zoological Society of London, 1881* (pp. 22–44, plates 3–5). London Academic Press.

[CR94] Smith AM, Achilleos K, Gordon DP, Key MM, Porter JS, Wyse Jackson PN (2023). Distribution patterns of shelf bryozoans around southern Aotearoa New Zealand. Bryozoan Studies 2022: Proceedings of the 19th International Bryozoology Association Conference, Dublin, Ireland, 22 - 26 August 2022.

[CR95] Soule JD (1950). Penetrantiidae and Immergentiidae from the Pacific (Bryozoa: Ctenostomata). Transactions of the American Microscopical Society.

[CR96] Soule JD, Soule DF (1969). Systematics and Biogeography of Burrowing Bryozoans. American Zoologist.

[CR97] Soule JD, Soule DF, Pouyet S (1975). Spathipora, its anatomy and phylogenetic affinities. Bryozoa 1974: Documents des laboratoires de géologie de la Faculté des sciences de Lyon.

[CR98] Strebel H (1904). Beiträge zur Kenntnis der Molluskenfauna der Magelhaen-Provinz. Zoologische Jahrbücher, Abteilung für Systematik, Geographie und Biologie der Tiere.

[CR99] Strebel H (1905). Beiträge zur Kenntnis der Molluskenfauna der Magelhaen-Provinz, No. 3.Z oologische Jahrbücher. Abteilung für Systematik, Geographie und Biologie der Tiere.

[CR100] Tamura K, Nei M, Kumar S (2004). Prospects for inferring very large phylogenies by using the neighbor-joining method. Proceedings of the National Academy of Sciences USA.

[CR101] Tamura K, Stecher G, Kumar S (2021). MEGA11: Molecular evolutionary genetics analysis version 11. Molecular Biology and Evolution.

[CR102] Taylor PD, Ernst A, Webby BD, Paris F, Droser ML, Percival IG (2004). Bryozoans. The Great Ordovician Biodiversification Event.

[CR103] Taylor PD, Rozhnov S (1996). A new early cyclostome bryozoan from the Lower Ordovician (Volkhov Stage) of Russia. Paläontologische Zeitschrift.

[CR104] Taylor PD, Waeschenbach A (2015). Phylogeny and diversification of bryozoans. Palaeontology.

[CR105] Taylor, P., & Larwood, G. P. (Ed.). (1990). *Major Evolutionary Radiations* (The Systematics Association Special Vol. 42). Systematics Association.

[CR106] Thorpe JP, Clarke DRK, Best MA, Nielsen C, Larwood GP (1985). Natural variation in tentacle number in marine bryozoans and the possible effects of intraspecific and interspecific ecological competition for food. Bryozoa: Ordovician to Recent.

[CR107] Turton W (1825). Description of some new British shells. Zoological Journal.

[CR108] Weber AV, Wanninger A, Schwaha TF (2014). The nervous system of *Paludicella articulata* - first evidence of a neuroepithelium in a ctenostome ectoproct. Frontiers in Zoology.

[CR109] White A (1847). Descriptions of new or little-known Crustacea in the collection of the British Museum. Proceedings of the Zoological Society of London.

[CR110] Wood ACL, Probert PK, Rowden AA, Smith AM (2012). Complex habitat generated by marine bryozoans: A review of its distribution, structure, diversity, threats and conservation. Aquatic Conservation: Marine and Freshwater Ecosystems.

