# Supplementary material for: Digging into boring bryozoans: new characters and new species of Immergentiidae
Source: Org Divers Evol. 2024 Jun 27;24(2):217–56. doi: 10.1007/s13127-024-00645-y (PMC11258195; doi:10.1007/s13127-024-00645-y)
Supplement: Supplementary file 1 — Supplementary file1 (DOCX 5930 KB) [file 13127_2024_645_MOESM1_ESM.docx]

**Supplementary Information**

**Digging into boring bryozoans: new characters and new species of Immergentiidae**

Mildred J. Johnson^1$^, Ahmed J. Saadi^1^, Piotr Kuklinski^2^, Abigail M. Smith^3^, Juan López-Gappa^4,5^, Thomas Schwaha^1^

^1^ University of Vienna, Department of Evolutionary Biology, Djerassiplatz 1, 1030 Vienna, Austria

^2^ Institute of Oceanology, Polish Academy of Sciences, Sopot, Poland

^3^ University of Otago, Dept. Marine Sciences, New Zealand

^4^ Consejo Nacional de Investigaciones Científicas y Técnicas, (CONICET), Buenos Aires, Argentina

^5^ Museo Argentino de Ciencias Naturales, C1405DJR Ciudad Autónoma de Buenos Aires, Argentina

^$^ corresponding author: mildred.johnson@univie.ac.at

ORCID:

MJJ: 0000-0003-0987-1374

AJS: 0000-0002-5113-0441

PK: 0000-0002-1507-215X

AMS: 0000-0001-6468-9124

JLG: 0000-0002-9567-9401

TS: 0000-0003-0526-6791

Supplementary Information

**Table S1.** Locations where samples were collected and analyzed specimen numbers

| **Region** | **Sample** | **Cruise** | **Location** | **Date** | **Latitude** | **Longitude** | **Depth (m)** | **Specimen numbers** |
| --- | --- | --- | --- | --- | --- | --- | --- | --- |
|  | *I. patagoniana* | PD | Burdwood Bank: off Argentine Tierra del Fuego and south of the Malvinas/Falkland Islands | 30 Mar 2016 | 54°25.144' S | 59°12.892' W | 120 | ARG-E28-L51 |
|  |  |  |  | 08 May 2017 | 54°00.240' S | 61°04.762' W | 139 | ARG-E28-L290 |
|  |  |  |  | 10 Apr 2016 | 54°30.390' S | 59°48.654' W | 105 | ARG-E31-L198 |
|  |  |  |  | 31 Mar 2016 | 54°31.679' S | 61°27.979' W | 137 | ARG-E35-L88 (gastropods 1 & 2) |
|  |  |  |  |  |  |  |  |  |
| New Zealand | *I.* cf. *zelandica* | PB-10 | Otago inner shelf | 5 Nov 2021 | 45° 45.87' S | 170° 49.50' E | 40 | NZ21PB10 (gastropods 1, 2 & 3) |
|  |  |  |  |  |  |  |  |  |
|  | *I. pohowskii* sp. nov. | PB-11 | Subtidal Taiaroa Head | 5 Nov 2021 | 45° 45.36' S | 170° 47.36' E | 23 | NZ21PB11Ct1, NZ21PB11Ct2 |
|  |  |  |  |  |  |  |  |  |
| France | *I.* cf. *suecica* | *Neomysis* | Stolvezen | 3 Sept 2021 | 48°42.847' N  48°42.846' N  48°40.000' N | 3°53.500' W  3°53.5' W  3°52.999' W | 15 – 25 | FR21–GP21, FR21–A50, FR21–PF54, FR21–FP28, FR21–A28, FR21–GP28, FR21–A25, FR21–GP25, FR21–A22, FR21–GP12, FR22–72A, FR22–74A, FR23-A3 |
|  |  |  |  | 5 Oct 2021 |  |  |  | FR21–D6/7, Dried shells |
|  |  |  | Térenéz | 10 Sept 2021 | 48°41.532' N | 03°52.075' W | ≈10 | FR21–A55, Dried shells |
|  |  |  |  | 14 Sept 2021 |  |  |  |  |
|  |  |  |  | 5 Oct 2021 |  |  |  |  |
|  |  |  | Chateaux du Taureau | Sept 2020  4,5, 8 & 12 Sept 2021  Dec 2022 | 48°40.2' N  48°40.200' N | 3°53.12' W  3°52.999' W | 10 – 15 | FR19-6F, FR21-A6, FR22–33A, Dried shells |
|  |  |  |  |  |  |  |  |  |
|  | *I. stephanieae* sp. nov. |  | Intertidal zone Roscoff  Santec  Ill Callot | 20 Sept 2021  2 Sept 2021  3 Oct 2021 | 48°43.698'N  48°42.787' N  - | 3°59.721'W  4°1.315' W  - | Intertidal | FR19-30F, FR19-50F, FR20-59FP, FR21GP59, FR20-55A, FR20-56A, FR21-A61-gastropod1 (holotype + paratypes), FR21–A3, FR21–G3, FR21–A17, FR21–A20, FR21-GRYI, Dried shells |
|  |  |  |  |  |  |  |  |  |
| Norway | *I.* cf. *suecica* |  | Trondheim Fjord | 08 Jul 2014 | 63°51.479'N | 11°04.354'E | 12 | N19 gastropod 3 |

* All material located at the University of Vienna Biology Building (UBB), Vienna, Austria

**Table S2:** Primers used in this study to amplify the standard mitochondrial gene *cox1.*

| **Primer name** | **F – forward, R – reverse**  **F** | **Sequence (5’–3’)** | **Reference** |
| --- | --- | --- | --- |
| LCO1490 | Forward primer | GGTCAACAAATCATAAAGATATTGG | Folmer et al. 1994 |
| HCO2198 | Reverse primer | TAAACTTCAGGGTGACCAAAAAATCA | Folmer et al. 1994 |
| boring_coF | Forward primer | 5’-TCA ACT AAC CAT AAA GAC ATT GG-3’ | Decker et al. (2023b). This study |
| immer_coF | Forward primer | 5’-ATA TCA ACT AAC CAT AAG GAC AAT GG-3’ | This study |
| immer_coR | Reverse primer | 5’-TAT ACT TCT GGA TGC CCA AAA AAT CA-3’ | This study |
| penet_coF | Forward primer | 5’-ATG TCA ACT AAC CAT AAA GAC ATT GGC A-3’ | Decker et al. (2023b). This study |
| penet_coR | Reverse primer | 5’-TAG ACT TCT GGG TGT CCG AAG AAT CA-3’ | Decker et al. (2023b). This study |

**Table S3**. Terminology for cystid appendages of recent immergentiids

| **Terms used in this study** | **Terms in literature** | **Reference** |
| --- | --- | --- |
| Primary cystid appendage |  |  |
|  | anastomosing stolon | Soule & Soule 1969 |
|  | cystid appendages | Schwaha, 2021a |
|  | interzoid stolon-like connections | Soule 1950 |
|  | lateral stolon | Pohowsky, 1978 |
|  | parent stolon | Pohowsky, 1978 |
|  | primary stolon | López-Gappa 1981 |
|  | principal stolon | Pohowsky, 1978 |
|  | thread-like processes | Silén 1946 |
|  | threads | Silén, 1947 |
|  | stolon | Soule, 1950; Soule & Soule 1969; Pohowsky 1978; Seo et al. 2018 |
|  | stolonal path | Soule, 1950 |
|  | stoloniform processes | López-Gappa 1981 |
|  | stolo-like threads | Silén, 1947 |
| Secondary cystid appendage |  |  |
|  | additional processes | Schwaha, 2021a |
|  | adventitious stolon | Pohowsky, 1978 |
|  | lateral branches | Silén, 1947 |
|  | lateral stolons | Pohowsky, 1978 |
|  | secondary processes | Silén, 1946; López-Gappa, 1981 |

**Table S4**. Summary of zooidal metrics and characteristics of recent immergentiids in this study.

| ***Immergentia***  **Species** | **Tentacle no.** | **Mean zooid length (µm)** | | **Mean zooid width (µm)** | **Mean aperture width (µm)** | **Distance between zooids (µm)** | **Borehole aperture shape** | **Zooidal characteristics** |
| --- | --- | --- | --- | --- | --- | --- | --- | --- |
| *I. californica* | 10 | 319 ± SD 21  Min - max: 284 – 357 | | 90 ± SD 17  Min - max: 63 – 124 | - | - | Oval to spindle shaped | Zooids with rounded or tapered basal tip.  Zooids vertical in substrate or curved slightly toward direction of primary cystid appendage. |
|  |  | n = 7 | | | **-** | **-** |  |  |
| *I. stephanieae* sp. nov. | 9 or 10 | 345 ± SD 23  Min - max: 313 – 381 | | 129 ± SD 19  Min - max: 103 – 165 | 66 ± SD 27  Min - max: 35 – 117 | 292 ± SD 83  Min - max: 125 –429 | Oval to spindle shaped. Weakly enantiomorphic.  One end of the spindle may have a small rounded nudge before extending into primary cystid appendage (distal). | Zooids regularly spaced.  Low positioned-lophophoral anus.  Some individuals with wider apertures than other individuals but comparable length.  Vase shaped, rounded or sometimes tapered proximal tip slightly  curved toward cystid appendage.  Proximal cystid appendage present.  Embryo develops in degenerated zooid.  Maximum secondary cystid appendages = 4  Intercalary kenozooids |
|  |  | n = 8 | | | n = 46 | n = 10 |  |  |
| *I.* *suecica* | 9 | 250 ± SD 11  Min - max: 240 – 262  Small zooid 131µm | | 68 ± SD 5  Min - max: 64 – 74  Small zooid  62 | 55 ± SD 9  Min - max: 44  – 67 | - | Oval | Lophophoral anus.  Vase shaped  autozooid, typical of *Immergentia*. Zooids slightly tapered or rounded proximal  Intercalary kenozooids |
|  |  | n = 3 | | | n = 7 |  |  |  |
| *I.* cf. *suecica* France | 9 | 262 ± SD 48  Min - max: 119 – 362 | | 74 ± SD 10  Min - max: 59 – 98 | 50 ± SD 9  Min - max:  34 – 79 | 917 ± SD 175  Min - max: 624 – 1258 | Enantiomorphic + Oval  Zooids irregularly spaced and not in a straight line. | Low positioned-lophophoral anus.  Vase shaped  autozooid, typical of *Immergentia*. Some zooids slightly tapered proximal end but most with rounded end. May lay parallel to primary cystid appendage or slightly  curved toward cystid appendage.  Embryo develops in degenerated zooid.  Maximum secondary cystid appendages = 3  Intercalary kenozooids |
|  |  | n = 26 | | n = 26 | n = 59 | n = 10 |  |  |
| *I*. cf. *suecica* Norway | 9 | 303 ± SD 25  Min - max: 268 – 342 | | 100 ± SD 32  Min - max: 67 – 106 | 45 ± SD 9  Min - max:  35 – 56 | 952 ± SD 365  Min - max: 578 – 1094 | Aperture oval shaped. Weakly enantiomorphic.  Zooids occur at regular intervals, widely spaced. | Vase shaped  autozooid, typical of *Immergentia*. Some zooids with rounded tapered proximal end slightly  curved toward cystid appendage or rounded tip.  Transition from end of midgut to hindgut severely pinched.  Mid-lophophoral anus.  Embryo develops in degenerated zooid.  Maximum secondary cystid appendages = 3  Intercalary kenozooids |
|  |  | n = 4 | | | n = 10 | n = 9 |  |  |
| *I. patagoniana* | 9 | 333 ± SD 26  Min - max: 302 – 352 | | 124 ± SD 32  Min - max:  102 – 180 | 36 ± SD 8  Min - max: 18 – 65 | 268 ± SD 63  Min - max: 177 – 325 | Spindle, bulged S, strongly enantiomorphic.  Zooids densely packed | Zooids vase shaped.  Sometimes zooids tapered toward proximal tip.  Some zooids may slightly curve at 45-90° angle toward the primary cystid appendage.  Few zooids with short narrow projection at basal end.  Low-positioned lophophoral anus.  Embryo develops in degenerated zooid  Maximum secondary cystid appendages = 3  Intercalary kenozooids |
|  |  | n = 5 | | | n = 71 | n = 5 |  |  |
| *Immergentia* *pohowskii* sp. nov. | 8 | 299 ± SD 30  Min - max: 237 – 345 | | 76 ± SD 8  Min - max: 59 – 85 | 35 ± SD 9  Min - max:  22 – 53 | 943 ± SD 118  Min - max: 540 – 1016 | Oval to spindle shaped. Enantiomorphic.  Zooids broadly spaced. | Shape of zooids cylindrical or typical vase shaped. Tapered or rounded proximal tip.  Mouth, pharynx area broader.  Proportion of gut and tentacles to rest of cystid wall relatively small than in any other species.  Low or mid positioned-lophophoral anus.  Cystid appendage at proximal tip  Embryo develops in degenerated zooid.  Zooids may have elongated projection extending proximally from the mid-zooidal region.  Maximum secondary cystid appendages = 3 maybe more  Intercalary kenozooids |
|  |  | n = 20 | | | n = 16 | n = 4 |  |  |
| *I. zelandica* | 9 | 249 ± SD 29  Min - max: 201 – 304 | 59 ± SD 12  Min - max: 92 – 92 | | 87 ± SD 14  Min - max:  63 – 124 | - | Circular | Zooids with rounded or tapered basal tip.  Zooids vertical in substrate or curved strongly toward direction of primary cystid appendage.  Few zooids with short narrow projection at basal end. |
|  |  | n = 10 | | | n = 20 | - |  |  |
| *I.* cf. *zelandica* | 9 | 284 ± SD 38  Min - max: 233 – 362 | | 85 ± SD 11  Min - max: 67 – 100 | 52 ± SD 6  Min - max:  43 – 65 | - | Oval to spindle shaped. Enantiomorphic.  Zooids irregularly and densely packed | Zooids with rounded basal tip.  Distorted zooids common.  Thinner processes occur on primary and secondary cystid appendages.  Few zooids may have elongated projection extending proximally from the mid-zooidal region.  Maximum secondary cystid appendages = 4  Intercalary kenozooids |
|  |  | n = 20 | | | n = 10 | n = 2 |  |  |

**Table S5.** Zooidal metrics and characteristics of recent and fossil immergentiids from literature. Fossil species highlighted in grey.

| ***Immergentia* species** | **Mean zooid length (µm)** | **Mean zooid width (µm)** | **tentacles** | **Borehole aperture shape** | **Location (type)** | **authority** | **Type Specimen** | **Substrate**  **(accepted name WoRMS)** | **Substrate condition** | **Description -**  **on which type is based** | **Zooidal characteristics** | **Depth of collection** | **Comments** |
| --- | --- | --- | --- | --- | --- | --- | --- | --- | --- | --- | --- | --- | --- |
| *angulata* | 180 – 200 | 50 – 55 | 8 | Ovoid, narrowing toward the thread-like trace made by the stolon. Well-spaced. | 1  Hawaiian Islands: Keaukaha, Hilo, USA | Soule & Soule, 1969 | Holotype, Allan Hancock Foundation bryozoan number 152.  Paratype number 152.1. | *Pisania tritonoides* (Reeve, 1846) | Possibly living and/or dead gastropods |  | Zooids bent sideways (acutely curved at the distal end below the aperture) and pointed  proximally | Collected 15 July 1967, depth 45  feet, water temperature 81 °F | Substrate condition not clearly stated. |
| *atypica* | – | – | NR | Nearly circular, slightly oval, 100µm wide | Waipara Gorge, New Zealand | Pohowsky, 1978 | Holotype BMNH D.5238S | *Maoricrypta* sp. (Finlay, 1926) |  | Dry specimen | Zooids orientated vertically in the substratum.  No apertural enantiomorphism | NR |  |
| *boydekina* | 300 – 320 | 43 | NR | Widely spaced, nearly ovoid, enantio-  morphic | Hamilton, Victoria, Australia | Pohowsky, 1978 | Holotype BMNH D.52381 and paratype BMNH G.39306-10 | *Conus* sp. (Linnaeus, 1758),  *status uncertain.  -Possibly *Amalda (Ancilla / Baryspira) glandiformis* (Lamarck, 1810)? |  | Cast (6 zooids) | Zooids sharply pointed proximally, laterally compressed, and narrowed distally, between well-developed vanes.  Tubulets absent. | NR | Unaccepted substrate name in citation: *Ancillaria inflata** (Deshayes, 1835) |
| *californica* | 350 | 100 | 10 | Regularly quadrangular | 2  Pacific Grove, California, USA | Silén, 1946 | SMNH-Type-2356 | *Tegula brunnea* (R. A. Philippi, 1849) | Alive |  | Zooid wide with rounded proximal portion end (evenly pointed) not lying parallel to stolonal path. Opposite branching of lateral “stolons” does not occur. | Tide pools | Collection of T. Skoosberg 1921 |
|  | 325 – 330 | 95 | 10 |  | California, USA |  |  | *Tegula ligulata*  (Menke, 1850), *Haliotis cracherodii* (Leach,1814), *Littorina scutulata* (Gould,1849),  *Tectarius striatus* (P. P. King, 1832) and *Acanthinucella spirata* (Blainville, 1832) | Living and dead |  | Description as type. | Tide pools  Whites Point, San Pedro,  January 27, 1949;  Portuguese Bend, March 11, 1949 | Reference Soule (1950). Description of species as by Silen (1946,1947)  Material (*T. ligulata*) also examined by Pohowsky.  Unaccepted substrate name in regference:  *Littorina planaxis* Nuttall*( G. B. Sowerby I, 1844),  *Acanthina spirata* (Blainville, 1832)*. From type location. |
| *cheongpodensis* | 150 | 50 | NR | Elongate  (teardrop shape to sausage shaped opening) | 3  Cheongpodae Beach, Yellow Sea, Korean West Coast | Seo et al., 2018 | Holotype MBRMKH7 and paratype MBRBKP7 (same data as holotype) | Mollusk shells, particularly oysters and clams | Dead | Shell boring shape, SEM of colony (Rensin casts) | Zooid teardrop shaped. Zooid widest at orificial end, non-pedunculate, with narrow, round-tipped basal end. Zooid excavation orientated at oblique angle, some even curving upward toward shell surface | 36.6334° N, 126.2997° E, intertidal, 16 June 2017. From low-intertidal zone. Cheongpodae, Taean Coast National Park. |  |
| *cruciata* | NR | NR |  |  |  | Magdefrau, 1937 |  |  |  |  |  |  | Species previously known as *Chaetophorites cruciatus* |
| *lanceolata* | 300 – 320 | 65 |  | Widely spaced, nearly ovoid, enantio-  morphic and sometimes shifted slightly toward one side of stolon (larger than *I. boydekina*) | Hamilton, Victoria, Australia | Pohowsky, 1978 | Holotype BMNH D.52383, paratype BMNH G.39704-9, shell B | *Polinices* sp. (Montfort, 1810) | Dry specimen | Cast (7 zooids) | Same length and general form as in *I. boydekina*  Tubulets absent |  |  |
| *losangelina* | 200 | 50 | NR | Elongated enantiomorphic apertures | Los Angeles, California | Pohowsky, 1978 | Holotype BMNH D.52386 | *Callianax biplicata* (G. B. Sowerby I, 1825) |  | Casts (3 zooids) | Strongly oval in cross section, with rounded proximal end.  Tubulets probably absent |  | Unaccepted substrate name in citation: *Olivella biplicata* *Swainson |
| *orbignyana* | 90 | 52 | NR | Oval or conical depression | 4 Arcachon, Gironde, France | Fischer, 1866 | Syntypes examined by Pohowsky: MNHN Di-0207-b | Bivalve | Dry specimen | *Immergentia* borings recognised and placement by Pohowsky (1978) | Species irregular, tightly packed, opposite branches |  | Description of species and drawings (by Fischer (Plate 11, Figures 2 and 2a; colony and zooids illustrations) are clearly immergentiids.  Several syntypes bear *Terebripora* and/or *Immergentia* |
| *patagoniana* | NR | NR | NR | Elongated, strongly enantiomorphic apertures | 5  Patagonia, Argentina | Pohowsky, 1978 | Holotype: BMNH D.52388 | *Buccinanops cochlidium* (Dillwyn, 1817) | Dry specimen |  | Location of zooids and stolons at the bottom of relatively broad depressions in the surface of the substratum. Often strongly curved zooids.  Tubulets present | NR | Preserved in two gastropods from the collection of d'Orbigny Unaccepted authority *Buccinanops*  *cochlidium* Kiener* |
| *zelandica patagonica* | 300 | 85 | 9 | Irregularly shaped holes, arranged very densely | 6  Santa Cruz, Argentina | López Gappa, 1981 | Holotype: CIBIMA N° 162a  Paratype: CIBIMA N° 162b | *Pareuthria fuscata* (Bruguière, 1789) | Alive | Rensin casts | Zooids arranged irregularly, connected to each other by processes that arise from their distal extremities. Secondary processes present. Zooids form an angle of 45 ° to 90 ° with the surface |  | Synonym for *I. patagoniana*, this study  Unaccepted substrate name in citation: *Pareuthria plumbea* (R. A. Philippi, 1844).  Description as type. |
| *philippinensis* | 310 | 57 | 10 | NR | Zamboanga, Philippine Islands | Soule, 1950 | Holotype: AHF number 51. | *Stomatella planulata* (Lamarck, 1816) | Alive |  | Zooid narrower with tapered pointed proximal end, lying horizontally directly beneath and parallel to the stolonal path  Tubulets absent | Tide pools. November 1947 | Dispute: Diagnosis anatomy and generic affinities of species are uncertain description based on *Terebripora*  Unaccepted substrate name in citation: *Gena planulata* Lamarck |
| *subangulata* | 200 – 240 | NR | NR | Widely spaced, nearly ovoid, enantio-  morphic and sometimes shifted slightly toward one side of the stolon (like *boydekina*) | 7  Bay of Santos, Brazil | Pohowsky, 1978 | Holotype: BMNH D.52384 | *Crepidula* sp. (Lamarck, 1799) | Dead | Casts (5 zooids) | Zooids gently flexed proximally (relative to the stolon), slightly distal to mid-length. Oval in cross section.  Tubulets absent | NR | Collection of Prof. Dr. E. Marcus gifted by Mrs E. Marcus |
| *suecica* | 310 – 340 | 80 | 9 |  | 8  Gullmar Fjord, West Coast of Sweden | Silén, 1947 | SMNH-Type-2366 | *Pseudamussium peslutrae* (Linnaeus, 1771) | Alive and fixed old material | Description based on 3 zooids (2 small, 1 large) | Distal area of zooid more circular.  Zooid slightly curved, cross section circular, slightly tapered toward rounded proximal end.  Zooids generally occur in straight rows. Adventitious stolons sometimes arise from zooids; tubulets sometimes present on stolons. | 45m, Gullmar Fiord, north of Flatholmen | Unaccepted species name in citation: *Pecten septemradiatus* (O. F. Müller, 1776) |
| *zelandica* | 210 – 310 | 80 | 9 | Regularly quadrangular (general zooidal apertual shape not borehole) | 9  Slipper Island, New Zealand | Silén, 1946 | SMNH-Type-3065 | *Buccinulum littorinoides* (Reeve, 1846) | Alive | Living gastropod shell | Abanal part of proximal end forms distinct finger-shaped short projection where proximal end of retractor muscle is fixed. | Near shore at low tide | Unaccepted species name in citation. *Euthria strebeli* (Suter, 1908) |
| *zelandica* var. *minuta* | 207 – 218 | 48 | 9 | NR | 10  Zamboanga, Philippine Islands | Soule, 1950 | AHF number 52 | *Stomatella planulata* (Lamarck, 1816) | Alive |  | Zooids very short, proximal ends of the zoids with a short projection | Tide pools | Description as type but smaller. Unaccepted species name in citation*. Gena planulata*  (Soule 1950) |
|  | 210 – 250 | NR | 9 | NR | From Hawaiian Islands | Soule & Soule 1969 |  | *Conus striatus* (Linnaeus, 1758)  *Cypraea* sp.? (Linnaeus, 1758) | NR |  |  | Haena Bay, Kauai; collected 1 July 1967, depth 20 feet,  water temperature 80°F. | Description as above. Not type. |

NR = Not Reported


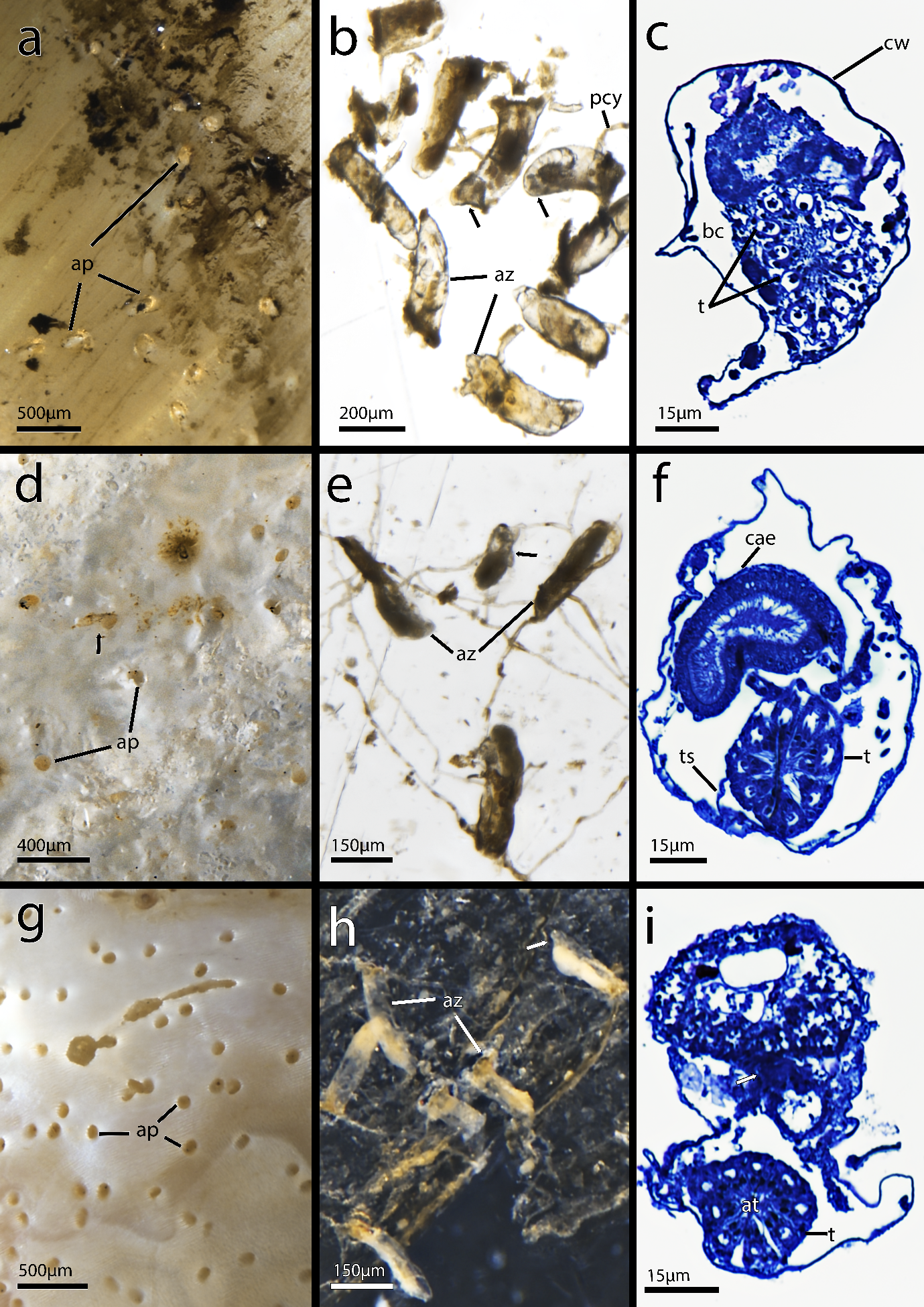


**Fig. S1. Holotype material. a-c *Immergentia californica*** (Locality: Pacific Grove, California, USA; SMNH-Type-2356). **a** Borehole apertues of *Immergentia californica*, oval to spindle shaped. **b** Zooids of *Immergentia californica*, in a degraded condition. Basal end curved toward direction of primary cystid appendage (arrow). **c** Semi-thin histological serial section of retracted *Immergentia californica* polypide with 9 tentacles. **d-f *Immergentia suecica*** (Locality: Fjord, West Coast of Sweden; SMNH-Type-2366 ). **d** Borehole apertures of *Immergentia* *suecica*, oval shaped, in eroded shell. Part of zooid protruding from borehole aperture (arrow)  **e** Zooids of *Immergentia suecica*. Smaller of the three zooids presumed developing zooid (arrow). **f** Cross section of retracted *Immergentia suecica* polypide with 9 tentacles and caecum. **g-i *Immergentia zelandica*** (Locality: Slipper island, New Zealand; SMNH-Type-3065). **g** Oval shaped borehole apertures of *Immergentia zelandica*. **h** Autozooids of *Immergentia zelandica*. Zooid with short narrowed projection at basal tip (arrow). **i** Cross section of *Immergentia zelandica* polypide with 9 tentacles. Transition from fore- to mid-gut (arrow).

Abbreviations: ap – aperture, at – atrium, az – autozooid, bc – body cavity, cae – caecum, cw – cystid wall, pcy – primary cystid appendage, t – tentacles, ts – tentacle sheath
